# Oxonitrogenated Derivatives of Eremophilans and Eudesmans: Antiproliferative and Anti-*Trypanosoma cruzi* Activity

**DOI:** 10.3390/molecules27103067

**Published:** 2022-05-10

**Authors:** María F. Beer, Guillermo F. Reta, Adrián Puerta, Augusto E. Bivona, Andrés Sánchez Alberti, Natacha Cerny, Emilio L. Malchiodi, Carlos E. Tonn, José M. Padrón, Valeria P. Sülsen, Osvaldo J. Donadel

**Affiliations:** 1 INTEQUI-CONICET, Facultad de Química, Bioquímica y Farmacia, Universidad Nacional de San Luis, San Luis 1445, Argentina; mflorenciabeer@gmail.com (M.F.B.); gfreta@gmail.com (G.F.R.); carlos.tonn@gmail.com (C.E.T.); 2 Instituto de Química y Metabolismo del Fármaco (IQUIMEFA), CONICET–Universidad de Buenos Aires, Buenos Aires 1113, Argentina; 3 BioLab, Instituto Universitario de Bio-Orgánica Antonio González (IUBO-AG), Universidad de La Laguna, Avda, Astrofísico Francisco Sánchez 2, 38206 La Laguna, Spain; apuertaa@ull.es (A.P.); jmpadron@ull.es (J.M.P.); 4 Cátedra de Inmunología, Facultad de Farmacia y Bioquímica, Universidad de Buenos Aires, Buenos Aires 1113, Argentina; augustobivona@gmail.com (A.E.B.); andres.sanchez.alberti@gmail.com (A.S.A.); emalchio@ffyb.uba.ar (E.L.M.); 5 Instituto de Estudios de la Inmunidad Humoral (IDEHU), CONICET–Universidad de Buenos Aires, Buenos Aires 1113, Argentina; natachacerny@gmail.com; 6 Instituto de Microbiología y Parasitología Médica (IMPaM), CONICET–Universidad de Buenos Aires, Buenos Aires 2155, Argentina; 7 Cátedra de Farmacognosia, Facultad de Farmacia y Bioquímica, Universidad de Buenos Aires, Buenos Aires 1113, Argentina

**Keywords:** sesquiterpenes, antiproliferative activity, Asteraceae, tessaric acid, ilicic acid, ilicic alcohol, *Trypanosoma cruzi*

## Abstract

Cancer is one of the most important causes of death worldwide. Solid tumors represent the vast majority of cancers (>90%), and the chemotherapeutic agents used for their treatment are still characterized by variable efficacy and toxicity. Sesquiterpenes are a group of natural compounds that have shown a wide range of biological activities, including cytotoxic and antiparasitic activity, among others. The antiproliferative activity of natural sesquiterpenes, tessaric acid, ilicic acid, and ilicic alcohol and their semisynthetic derivatives against HeLa, T-47D, WiDr, A549, HBL-100, and SW1573 cell lines were evaluated. The effect of the compounds on *Trypanosoma cruzi* epimastigotes was also assessed. The selectivity index was calculated using murine splenocytes. Derivatives **13** and **15** were the most antiproliferative compounds, with GI_50_ values ranging between 5.3 (±0.32) and 14 (±0.90) μM, in all cell lines tested. The presence of 1,2,3-triazole groups in derivatives 15–19 led to improvements in activity compared to those corresponding to the starting natural product (**3**), with GI_50_ values ranging between 12 (±1.5) and 17 (±1.1) μM and **16** being the most active compound. In relation to the anti-*T. cruzi* activity, derivatives **7** and **16** obtained from tessaric acid and ilicic acid were among the most active and selective compounds with IC_50_ values of 9.3 and 8.8 µM (SI = 8.0 and 9.4), respectively.

## 1. Introduction

Nature constitutes an important resource for the discovery of new bioactive compounds of interest. Plants are constituted by about 250,000 species, of which only about 6% have been studied for their biological activities, and only about 15% have been studied phytochemically [1].

It can be said that the plant kingdom is the largest source of chemical diversity, constituting a reservoir of chemical structures difficult to find in another environment.

**Figure 1:**
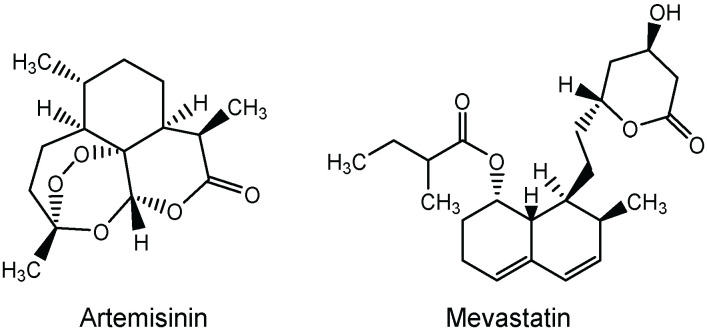
Natural compounds used in antimalarial therapy, artemisinin and mevastatin.

Since ancient times, medicinal plants have been of great importance as the only resource to treat a large number of diseases: *Papaver somniferum* (Papaveraceae), used as an analgesic and antitussive (morphine and codeine); *Digitalis lanata* (Plantaginaceae), source of digoxin (treatment of heart problems); taxol (paclitaxel), isolated from *Taxus brevifolia* (Taxaceae) and used for the treatment of different types of cancer. Among the natural compounds currently used in antimalarial therapy, we can mention artemisinin (isolated from *Artemisia annua* L., Figure 1) and quinine or cinchona (produced by some species of the genus *Cinchona* [2]).

This situation determined that the interest in natural products increased oriented to the search for chemical diversity and the obtention of new “leaders” [3]. Currently, natural products (NP) constitute a strategic starting point for the discovery of new drugs, since they exhibit a wide range of pharmacophores and a large number of pre-established chiral centers, and they allow interaction with a wide variety of proteins and biological targets. An example is rosuvastatin, which is widely used to reduce high cholesterol levels and whose structure is based on the pharmacophore of the natural product mevastatin (Figure 1) produced by the fungus *Penicillium citrinum* [4].

According to a study carried out by Newman and Cragg between 1981 and 2019 [5], 60% of the new chemical entities identified are natural products, semi-synthetic analogs, or synthesized compounds based on their pharmacophores.

The Asteraceae family, also known as Compositae, includes a large number of plants comprising approximately 1700 genera and 24,000 species [6]. It is the largest plant family in the Argentine Republic, with 227 genera (five of which are endemic) and about 1400 species [7]. This family includes food, medicinal, ornamental and industrial plants, weeds, and toxic plants.

Many species belonging to this family are known for their economic importance, either for their use in human nutrition (such as seeds and vegetables), ornamental interest, and for their pharmaceutical or industrial use, due to the presence of a large number of secondary metabolites [8].

The species *Tessaria absinthioides* Hook et Arm DC and *Flourensia oolepis* Blake, belonging to the Asteraceae family, are characterized by secondary metabolites of a terpene nature, mainly sesquiterpenes with eremophilan and eudesman nuclei. In previous works, we have reported improvements in the activity of sesquiterpene lactones isolated from *Ambrosia*, *Gaillardia*, *Parthenium*, and oxygenated and oxo-nitrogenated derivatives obtained from NPs [9,10].

In this work, we report 16 new compounds derived from natural sesquitepenes isolated from *T. absinthioides* and *F. oolepis* and their antiproliferative and trypanocidal activity.

## 2. Results and Discussion

### 2.1. Chemistry

**Figure 2:**
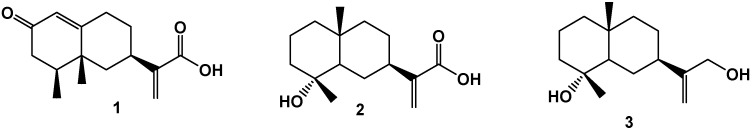
Structures of natural sesquiterpene tessaric acid (**1**), ilicic acid (**2**), and ilicic alcohol (**3**).

The eremophilan tessaric acid (**1**) and the eudesmans ilicic acid (**2**) and ilicic alcohol (**3**) were isolated from *Tessaria absinthioides* and *Flourensia oolepis*, respectively (Figure 2).

**Figure 3:**
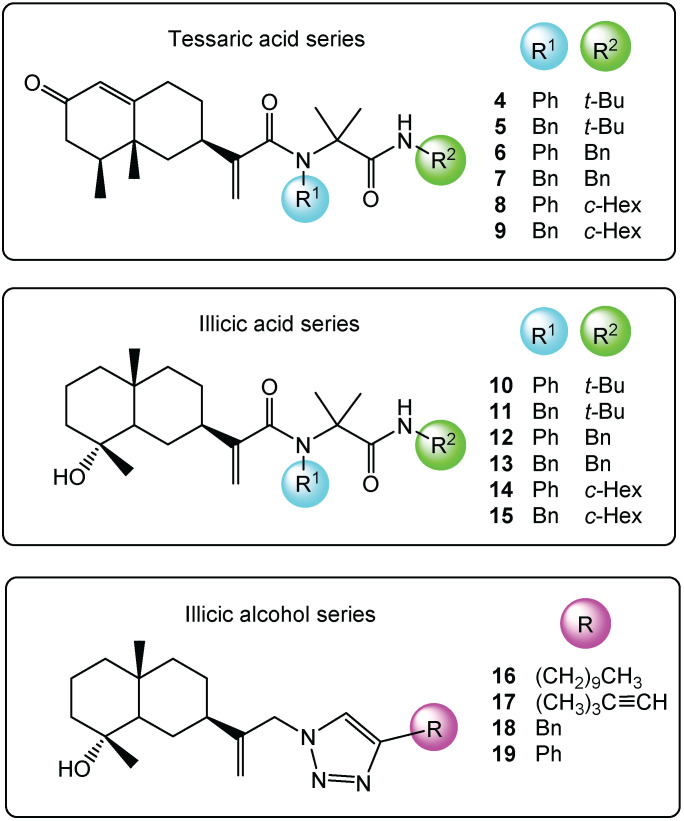
Structures of sesquiterpene derivative obtained from tessaric acid (**1**), ilicic acid (**2**), and ilicic alcohol (**3**).

In preliminary tests of bioactivity, these natural sesquiterpenes did not show cytotoxic activity but oxygenated derivatives did [11]. Therefore, these NPs have been used as starting materials for the preparation of a series of oxo-nitrogenated products. These modifications led to derivatives with an improvement in the activity. Derivatives **4**–**19** were prepared in this way (Figure 3).

Multicomponent and cycloaddition reactions are convergent procedures of high synthetic utility that combine chemical and atomic efficiency. Within the second type, the azidealkyne Huisgen cycloaddition is a powerful tool to prepare 1,2,3-triazoles in a simple way [12]. Multicomponent reactions (MCR) are convergent reactions in which three or more starting materials react to form a product where essentially all or most of the atoms contribute to the newly formed product. In MCRs, a product is assembled according to a cascade of elementary chemical reactions. Therefore, there is an equilibrium network of reaction that eventually leads to an irreversible step that produces the product. In this work, a series of oxy-nitrogenated derivatives were prepared, with compounds **4**–**15** by the Ugi reaction and **16**–**19** by Huisgen cycloadditions. It should be noted that in all the proposals the stereochemistry of the natural product remained intact.

**Scheme 1:**
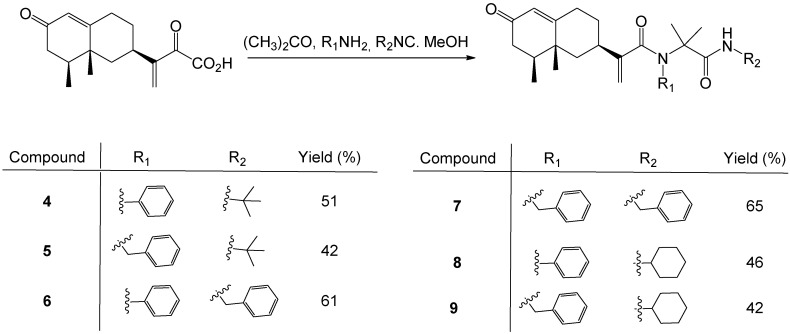
Synthesis of compounds **4**–**9**.

Derivatives **4**–**9** were obtained by the Ugi reaction using **1** as carboxylic acid and acetone as a source of carbonyl group. Aniline (compounds **4**, **6**, and **8**) and benzylamine (compounds **5**, **7**, and **9**) were used as amines varying the corresponding isocyanides (Scheme 1).

**Scheme 2:**
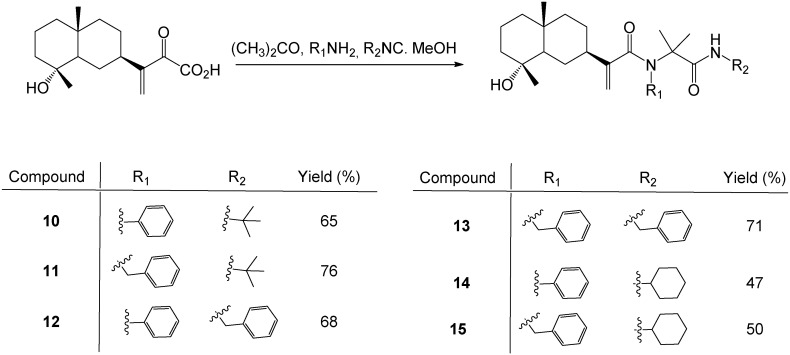
Synthesis of compounds **10**–**15**.

Derivatives **10**–**15** were obtained by the Ugi reaction using **2** as carboxylic acid and acetone as a source of the carbonyl group. Aniline (compounds **10**, **12**, and **14**) and benzylamine (compounds **11**, **13**, and **15**) were used as amines varying the corresponding isocyanides (Scheme 2).

**Scheme 3:**
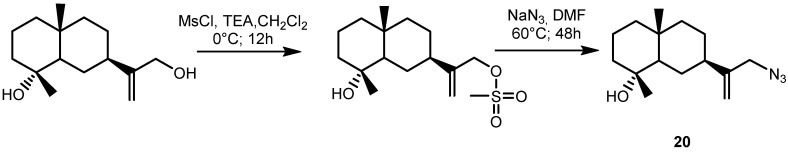
Synthesis of azide **20**.

Synthetic organic chemistry has aroused great interest in 1,2,3-triazoles in the development of new biologically active molecules [12]. The triazole moiety is not found in nature, but 1,2,3-triazole nuclei can form the basis of small molecule pharmaceuticals. Molecules containing this heterocyclic core have been reported to have anti-HIV, antimicrobial, antiallergic, antifungal, and antitumor activities [13]. One of the most popular reactions within the click chemistry paradigm is the Cu(I)-catalyzed 1,3-dipolar Hüisgen cycloaddition of alkynes and azides. This reaction proceeds with great efficiency and selectivity in aqueous media and produces triazole fractions [14]. Our first objective was to obtain azide **20** using alcohol **3** as a starting material (Scheme 3).

**Scheme 4:**
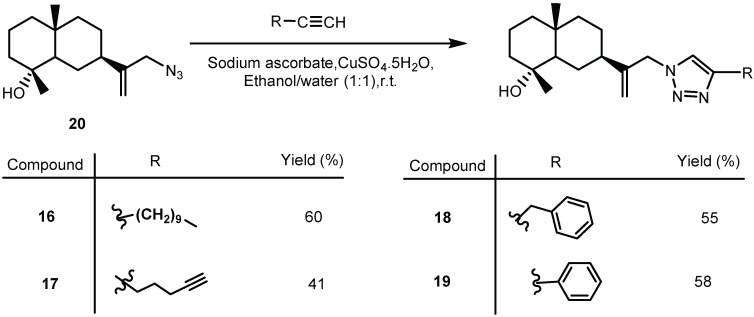
Synthesis of 1,2,3-triazoles **16**–**19**.

Coupling of the azide **20** and various commercial alkynes, under Huisgen conditions, gave the 1,2,3-triazoles **16**–**19** in good yields (Scheme 4).

### 2.2. Biological Results

#### 2.2.1. Antiproliferative Activity

**Figure 4:**
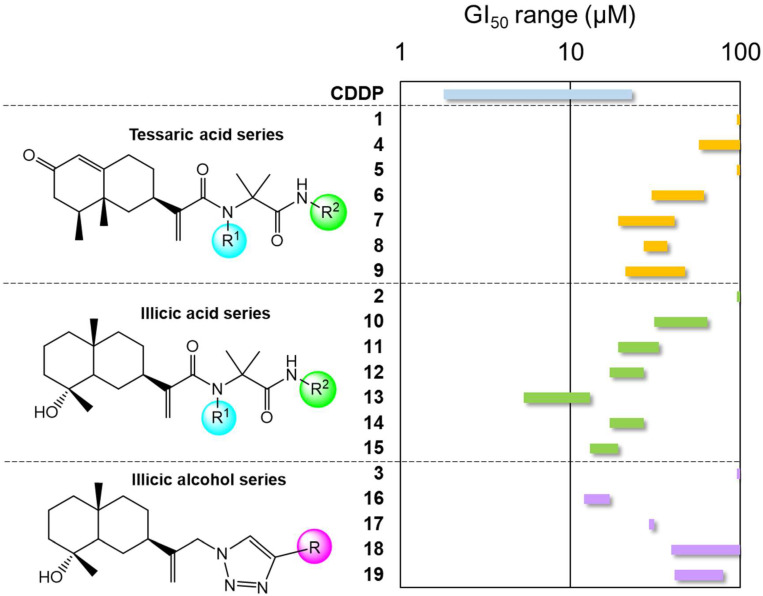
Range plot of antiproliferative activity for compounds **1**–**19**. Bars represent the range between minimal and maximal GI_50_ values (Table 1).

In vitro antiproliferative activity was evaluated using the protocol of the National Cancer Institute (NCI) after 48 h of drug exposure using the sulforhodamine B (SRB) assay. The results, expressed as GI_50_ values, are shown in Table 1 and Figure 4 [15].

The data revealed that almost all the synthesized oxo-nitrogen derivatives were more active than the natural products (**1**–**3**); compounds **6**–**12** and **14** showed moderate to good activity with GI_50_ values ranging between 17 (±5.6) and 64 (±1.5) μM, in all cell lines. On the other hand, derivatives **13** and **15** were the most active, with GI_50_ values that ranged between 5.3 (±0.32) and 14 (±0.90) μM.

The presence of 1,2,3-triazole groups in derivatives **16**–**19** led to improvements in activity values compared to those corresponding to the starting natural product (**3**), with **16** being the most active compound and with GI_50_ values that ranged between 12 (±1.5) and 17 (±1.1) μM.

The results are expressed as 50% growth inhibition (GI_50_) ± SD and are the mean of at least two independent experiments.

#### 2.2.2. Cytotoxicity on Primary Cell Culture

The cytotoxicity of the natural sesquiterpenes (**1**–**3**) and derivatives (**4**–**19**) was determined on primary cell cultures (splenocytes obtained from Balb/c mouse). Tessaric acid (**1**), ilicic acid (**2**), and ilicic alcohol (**3**) showed no toxicity to mammalian cells (CC_50_ > 329 µM). Derivatives of tessaric acid have shown an improvement in CC_50_ values in comparison with the natural sesquiterpene 1. Compound **6** was the least cytotoxic (CC_50_ = 103.6 µM). With regards to the derivatives of ilicic acid (**2**), compounds **10** and **12** were the less cytotoxic of the series with CC_50_ values of 78.2 and 64.3 µM, respectively. All the derivatives synthesized from alcohol ilicic (compounds **10**–**19**) were more toxic to mammalian cells than the natural compound (**3**). Compound **19** presented the highest CC_50_ value of the series (186.8 µM).

Selectivity indices (SI) for the natural compounds and their derivatives were calculated through the ratio of CC_50_ values in splenocytes and the GI_50_ on tumor cells. As it can be seen in Table 2, the derivatives obtained from tessaric acid (compounds **4**–**9**) showed no selectivity against the tumor cells tested (IS values between 0.5 and 3.6). The derivative **11** from ilicic acid was one of the most selective compounds with IS values of 17.6 (HeLa), 14.5 (T-47D), 11.5 (WiDr), 16.7 (A549), and 10.1 (HBL-100 and SW1573). Compound **14** presented IS values greater than 15 on the tumor cell lines evaluated. The triazole derivative of ilicic alcohol, compound **16**, was the most selective on the cell lines evaluated, with IS values of 6.3 (HeLa), 4.8 (T47-D) and 6.8 (WiDr). Compounds **17** and **19** were less selective on HeLa and T47-D lines with IS values between 1.3 and 3.1. In relation to the WiDr cell line, compound **19** presented an IS value of 4.6.

The results are expressed as cytotoxic activity (CC_50_) ± SD and are the mean of at least two independent experiments.

#### 2.2.3. Anti-*Trypanosoma cruzi* Activity

**Figure 5:**
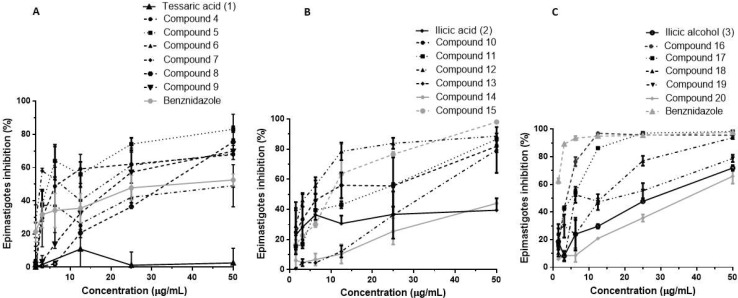
Effect of the natural sesquiterpene tessaric acid (**1**), ilicic acid (**2**), and ilicic alcohol (**3**) and their derivatives on *T. cruzi*. Epimastigotes (RA strain) were cultured in the presence of the compounds (50–1.5 µg/mL). Determinations were performed by triplicate. The results are expressed as the mean ± SD. (**A**) Effect of tessaric acid and derivatives on epimastigotes; (**B**) Effect of ilicic acid and derivatives on epimastigotes; (**C**) Effect of ilicic alcohol and derivatives on epimastigotes.

The natural sesquiterterpenes tessaric acid (**1**), ilicic acid (**2**), and ilicic alcohol (**3**) as well as their derivatives were evaluated against *T. cruzi* epimastigotes. The effect of the compounds on the parasite is shown in Figure 5.

As can be seen in Figure 5A, compounds **5** and **7** were the most active derivatives obtained from tessaric acid (**1**), against *T. cruzi* epimastigotes (IC_50_ = 9.3 and 4.7 µM, respectively) (Table 3). In relation to ilicic acid (**2**), compound **12** showed the best effect on *T. cruzi* with an IC_50_ value of 8.0 µM (Figure 5B and Table 3). Derivatives **16** and **17**, synthesized from ilicic alcohol, were active against epimastigotes with IC_50_ values of 8.8 and 14.8 μM, respectively, while compounds **18** and **19** were moderately active (IC_50_ = 30.2 μM and 48.2 μM, respectively) (Figure 5C and Table 3).

The tessaric acid derivative **7** presented a SI of 8.0 on *T. cruzi* epimastigotes. The other derivatives obtained from this sesquiterpene presented SI values in the range of 0.3 to 4.8. In relation to the triazole-type oxonitrogenated derivatives obtained from ilicic alcohol, compound **16** was the one that presented the highest selectivity index on epimastigotes (SI = 9.4), while derivatives **17** and **18** were less selective on this parasite (SI of 2.7 and 2.1, respectively). Derivative **11**, obtained from ilicic acid, was the one with the highest selectivity over epimastigotes in comparison to the other compounds (SI = 12.6) (Table 3).

Since these tests were conducted with *T. cruzi* epimastigotes, it will be necessary to perform further studies with the clinically more relevant form, intracellular amastigotes, to obtain a better estimate of these compounds’ usefulness against this parasite.

## 3. Materials and Methods

### 3.1. General

Unless otherwise stated, all solvents were purified by standard techniques. Reactions requiring anhydrous conditions were performed under an argon atmosphere. Anhydrous magnesium sulfate was used for drying solutions. Reactions were monitored by thin layer chromatography (TLC) on silica gel plates (60 F254) and were visualized with UV light, 2.5% phosphomolybdic acid in ethanol, or vanillin with acetic and sulfuric acid in ethanol with heating. Purification was performed by column chromatography (CC) on silica gel (230–400 mesh) using *n*-hexane and ethyl acetate gradient as the solvent. ^1^H-NMR spectra were recorded on a Bruker (Fallanden, Switzerland) 200, or 600 MHz; ^13^C-NMR spectra were recorded at 50 and 125 MHz; and chemical shifts were reported relative to internal Me_4_Si (δ = 0). Melting points were determined by using an Electrothermal IA9000 melting point apparatus (Essex, UK); the results are reported in degrees Celsius and are uncorrected. Optical rotations were recorded in a 343 Perkin Elmer polarimeter (Waltham, MA, USA). High-resolution ESI mass spectra were obtained from a Fourier transform ion cyclotron resonance (FT-ICR) mass spectrometer, a RF-only hexapole ion guide, and an external electrospray ion source. HRMS spectra were obtained on a Micromass AutoSpec mass spectrometer (Waters Co., Milford, MA, USA) (see Supplementary Materials).

### 3.2. Plant Material

The species used in the present work were *Tessaria absinthioides* Hook et Arm (Asteraceae) and *Flourensia oolepis* Baker (Asteraceae). All specimens were collected in February 2019, in El Volcán, San Luis Province, Argentina and were identified by Prof. Ing. Luis Del Vitto. *T. absinthioides* was registered as Del Vitto and Petenatti under voucher numbers N° 2329 and N° 461. *F. oolepis* was registered as Del Vitto and Petenatti, N° 4633, 1672-UNSL. The specimens of both species are deposited in the herbarium of the National University of San Luis.

#### 3.2.1. Isolation of Tessaric Acid from *T. absinthioides*

The aerial parts of *T. absinthioides* (2.0 kg) were extracted at room temperature with EtOH (3 × 48 h), and the ethanolic extracts obtained were concentrated under reduced pressure. The residue was suspended in 2 L of 10% sodium bicarbonate and extracted with CH_2_Cl_2_ (4 × 0.5 L). The aqueous fraction was acidified with 10% HCl and partitioned using CH_2_Cl_2_ (4 × 0.5 L). The CH_2_Cl_2_ extracts from this operation were combined for subsequent fractionation. This procedure was performed as described in the literature [16].

Under these conditions, 6 g of tessaric acid (**1**) were obtained, identified by spectroscopic and spectrometric methods (NMR, IR, and HRMS or HRMS-ES).

#### 3.2.2. Isolation of Ilici Alcohol and Ilicic Acid from *F. oolepis*

The aerial parts of *F. oolepis* (2.0 kg) were extracted at room temperature with *n*-hexane for 24 h, carrying out a degreasing process. After filtering, the residue obtained was extracted with EtOAc for another 24 h. The organic extract was divided into two parts, and both were taken to dryness, leaving two residues of 20 g (Fraction A) and 30 g (Fraction B) each [17].

The 30 g fraction was taken up in ethyl ether. Subsequently, a partition was made with a 10% aqueous solution of sodium bicarbonate (3 × 200 mL). The aqueous solution was acidified with 0.1 M hydrochloric acid solution (acid added to litmus paper turn) and then liquid/liquid partitioned with ethyl ether (4 × 150 mL). Fraction A (20 g) was subjected to separation by column chromatography with hexane-ethyl acetate mixtures of increasing polarity.

In this process, 4.6 g of ilicic acid (**2**) were obtained from Fraction B and 3 g of ilicic alcohol (**3**) from Fraction A, which represents a yield of 2.3 g of ilicic acid and 1.5 g of ilicic alcohol per kg of dry plant.

### 3.3. Chemistry

The following is the general procedure for the Ugi reaction. A solution of acetone (1.2 eq, 0.472 mmol), and amine (aniline or benzylamine) (1 eq, 0.393 mmol) in methanol (0.7 mL, 0.5 M) were stirred for 3 h. After that, natural acid (1 eq, 0.393 mmol) and isocyanide (1.1 eq, 0.4323 mmol) were added. The reaction mixture was stirred for 24 h. The solvent was removed under reduced pressure, and the crude reaction mixture was purified by flash chromatography on silica gel to afford the desired product, Scheme 2 and Scheme 3.

Preparation of derivative **4**: This compound was prepared by general procedure for the Ugi reaction, using **1** as an acid, aniline, and *tert*-butyl-isocyanide to obtain 92 mg of **4** (51% yield), as an amorphous solid.

Preparation of derivative **5**: This compound was prepared by general procedure for the Ugi reaction, using **1** as an acid, benzylamine, and *tert*-butyl-isocyanide to obtain 40 mg of **5** (42% yield), as an amorphous solid.

Preparation of derivative **6**: This compound was prepared by general procedure for the Ugi reaction, using **1** as an acid, aniline, and benzylisocyanide to obtain 115 mg of **6** (61% yield), as an amorphous solid.

Preparation of derivative **7**: This compound was prepared by general procedure for the Ugi reaction, using **1** as an acid, benzylamine, and benzylisocyanide to obtain 120 mg of **7** (65% yield), as an amorphous solid.

Preparation of derivative **8**: This compound was prepared by general procedure for the Ugi reaction, using **1** as an acid, aniline, and cyclohexyl isocyanide to obtain 90 mg of **8** (46% yield), as an amorphous solid.

Preparation of derivative **9**: This compound was prepared by general procedure for the Ugi reaction, using **1** as an acid, benzylamine, and cyclohexyl isocyanide to obtain 82 mg of **9** (42% yield), as an amorphous solid.

Preparation of derivative **10**: This compound was prepared by general procedure for the Ugi reaction, using **2** as an acid, aniline, and *tert*-butyl-isocyanide to obtain 120 mg of **10** (65% yield), as an amorphous solid.

Preparation of derivative **11**: This compound was prepared by general procedure for the Ugi reaction, using **2** as an acid, benzylamine, and *tert*-butyl-isocyanide to obtain 140 mg of **11** (76% yield), as an amorphous solid.

Preparation of derivative **12**: This compound was prepared by general procedure for the Ugi reaction, using **2** as an acid, aniline, and benzylisocyanide to obtain 113 mg of **12** (68% yield), as an amorphous solid.

Preparation of derivative **13**: This compound was prepared by general procedure for the Ugi reaction, using **2** as an acid, benzylamine, and benzylisocyanide to obtain 110 mg of **13** (71% yield), as an amorphous solid.

Preparation of derivative **14**: This compound was prepared by general procedure for the Ugi reaction, using **2** as an acid, aniline, and cyclohexyl isocyanide to obtain 92 mg of **14** (47% yield), as an amorphous solid.

Preparation of the derivative **15**: This compound was prepared by general procedure for the Ugi reaction, using **2** as an acid, benzylamine, and cyclohexyl isocyanide to obtain 80 mg of **15** (50% yield), as an amorphous solid.

The following is the general procedure for the Huisgen reaction. A solution of 50 mg (1eg, 0.2 mmol) of the azide **20** and 1.2 eq of alkyne, in 2 mL (0.1 M) in a mixture of ethanol and water 1:1, then 12 mg (0.3 eq, 0.06 mmol) of sodium ascorbate and 3 mg (0.1 eq, 0.02 mmol) of sulfate CuSO_4_·5H_2_O, was stirred for 24 h at room temperature. The solvent was removed under reduced pressure, and the crude reaction mixture was purified by flash chromatography on silica gel to afford the desired product.

Preparation of derivative **16**: This compound was prepared by general procedure for the Huisgen reaction, using **20** as a starting material and 1-dodecyne as an alkyne to obtain 49 mg of **16** (60% yield), as like a colorless oil.

Preparation of derivative **17**: This compound was prepared by general procedure for the Huisgen reaction, using **20** as a starting material and 1,6-heptadiyne as an alkyne to obtain 27 mg of **17** (41% yield), as like a colorless oil.

Preparation of derivative **18**: This compound was prepared by general procedure for the Huisgen reaction, using **20** as starting material and 3-phenyl-1-propyne as an alkyne to obtain 39 mg of **18** (55% yield), as like a colorless oil.

Preparation of derivative **19**: This compound was prepared by general procedure for the Huisgen reaction, using **20** as a starting material and 2,3,4,6-tetracetyl-1-ethynyl-β-d-glucopyranose as an alkyne to obtain 40 mg of **19** (58% yield), as like a colorless oil.

### 3.4. Spectroscopic and Physical Data

Compound [**1**]: White solid, m.p.: 155–156 °C. [α]_D_^20^: −143.4 (*c* 1.7, CHCl_3_). IR (KBr; υ_max_: cm^−1^): 2974, 1709, 1620, 1460, 1306, 1248, 1080. ^1^H-NMR (δ = ppm, 200 MHz): 0.98 (3H, s, Me-15); 1.08 (3H, s, Me-14); 1.51–1.68 (1H, m, H-6); 1.76–1.93 (2H, m, H-6, H-8); 1.93–2.12 (1H, m, H-8); 2.23–2.40 (4H, m, H-4, H-3, H-3a, H-9); 2.48–2.69 (2H, m, H-9, H-7); 5.69 (1H, s, H-13); 5.90 (1H, s, H-1); 6.36 (1H, s, H-13). ^13^C-NMR (δ = ppm, 50 MHz): 15.4 (C-15); 19.1 (C-14); 28.9 (C-9); 29.2 (C-8); 32.3 (C-7); 35.8 (C-4); 39.4 (C-6); 40.3 (C-5); 42.0 (C-3); 125.3 (C-13); 125.7 (C-1); 143.8 (C11); 171.8 (C-12); 173.9 (C-10); 199.6 (C-2). HRMS-ES (*m*/*z*): [M + Na]^+^: Calcd. for C_15_H_19_O_3_Na: 293.1130; found: 293.1127.

Compound [**2**]: Yellowish solid, m.p.: 181–182 °C. [α]_D_^20^: −32.8 (*c* 5.1; CHCl_3_). IR (KBr; υ_max_: cm^−1^): 3431, 2929, 1691, 1452, 1244, 1041, 863. ^1^H-NMR (δ = ppm, 200 MHz): 0.91 (3H, s, Me-14); 1.03–1.17 (1H, m, H-9); 1.17–1.25 (1H, m, H-6); 1.11 (3H, s, Me-15); 1.25–1.39 (2H, m, H-3, H-5); 1.39–1.52 (4H, m, H-1, H-2, H-3, H-9); 1.55–1.71 (3H, m, H-2, H-8, H-8a); 1.76-1.89 (1H, m, H-3); 1.91–2.03 (1H, m, H-6); 2.40–2.66 (1H, m, H-7); 5.0 (1H, s, H-13); 6.29 (1H, s, H-13). ^13^C-NMR (δ = ppm, 50 MHz): 18.7 (C-14); 20.1 (C-8); 22.4 (C-15); 26.6 (C-6); 27.1 (C-2); 34.6 (C-10); 40.0 (C-7); 40.9 (C-9); 43.4 (C-3); 44.5 (C-1); 55.0 (C-5); 72.4 (C-4); 124.5 (C-13); 145.1 (C-11); 171.6 (C-12). HRMS-ES (*m*/*z*): [M + Na]^+^: Calcd. for C_15_H_19_O_3_Na: 275.1623; found: 275.1620.

Compound [**3**]: White solid, m.p.: 134–135 °C. [α]_D_^20^: −45.0 (*c* 2.2; CHCl_3_). IR (KBr; υ_max_: cm^−1^): 3265, 2924, 2845, 1605, 1444, 1385, 1171, 1072, 889. ^1^H-NMR (δ = ppm, 200 MHz): 0.90 (3H, s, Me-14); 1.088–1.15 (1H, m, H-9); 1.11 (3H, s, Me-15); 1.15–1.31 (3H, m, H-1, H-5, H-6); 1.35–1.47 (3H, m, H-1, H-3, H-9); 1.47–1.66 (4H, m, H-2, H-2a, H-8, H-8a); 1.73–1.86 (1H, m, H-3); 1.88–1.97 (1H, m, H-6); 1.99–2.21 (1H, m, H-7); 4.14 (2H, s, H-12); 4.91 (1H, s, H-13); 5.02 (1H, s, H-13). ^13^C-NMR (δ = ppm, 50 MHz): 18.7 (C-14); 20.1 (C-8); 22.6 (C-15); 26.5 (C-6); 27.1 (C-2); 34.6 (C-10); 41.0 (C-9); 41.8 (C-7); 43.3 (C-3); 44.6 (C-1); 54.8 (C-5); 65.2 (C-12); 72.4 (C-4); 107.9 (C-13); 153.9 (C-11). HRMS-ES (*m*/*z*): [M + Na]^+^: Calcd. for C_15_H_26_O_2_Na: 261.1830; found: 261.1831.

Compound [**4**]: White solid, m.p.: 50–52 °C. [α]_D_^20^: −73.2 (*c* 10.21; CHCl_3_). IR (KBr; υ_max_: cm^−1^): 3442, 2962, 2359, 1653, 1508, 1362, 1279, 1163, 706. ^1^H-NMR: (δ = ppm, 600 MHz): 0.94 (3H, d, *J* = 7 Hz, Me-15); 1.03 (3H, s, Me-14); 1.38–1.39 (6H, s, H-1′, H-2′); 1.41 (9H, s; H-7′; H-8′; H-9′); 1.47–1.50 (1H, m, H-6); 1.65–1.68 (1H, m, H-6); 1.68–1.72 (2H, m, H-8); 1.99–2.07 (2H; m; H-4, H-7); 2.16–2.22 (1H, m, H-9); 2.24–2.30 (2H, m, H-3); 2.46–2.53 (1H, m, H-9); 4.94 (1H, d, *J* = 2 Hz, H-13); 4.99 (1H, s, H-13); 5.70 (1H, s, H-5′); 5.80 (1H, s, H-1′); 7.31–7.33 (3H, m, H-11′, H-13′, H-15′); 7.23–7.25 (2H, m, H12′, H-14′). ^13^C-NMR (δ = ppm, 150 MHz):15.5 (C-15); 19.0 (C-14); 25.5, 25.6 (C-1′,C-2′); 28.6 (C-7′,8′,9′); 28.7 (C-8); 28.8 (C-9); 34.4 (C-7); 36.3 (C-4); 38.8 (C-6); 40.1 (C-5); 42.2 (C-3); 51.1 (C-6′); 63.1 (C-3′); 115.5 (C-13); 125.5 (C-1); 128.1 (C-13′); 128.6 (C-11′,C15′); 131.2 (C-12′,C14′); 139.8 (C-10′); 149.5 (C-11); 171.2 (C-12); 173.6 (C-4′); 173.7 (C-10); 199.2 (C-2). HRMS-ES (*m*/*z*) [M + Na]^+^: Calcd. for C_29_H_40_N_2_O_3_Na: 487.2937; found 487.2927.

Compound [**5**]: Yellow solid, m.p.: 65–67 °C. [α]_D_^20^: −13.7 (*c* 8.35; CHCl_3_). IR (KBr; υ_max_: cm^−1^): 3421, 2926, 2360, 1653, 1541, 1458, 1171. ^1^H-NMR: (δ = ppm, 200 MHz): 0.93 (3H, d, *J* = 7 Hz, Me-15); 1.05 (3H, s, Me-14); 1.36 (9H, s; H-7′; H-8′; H-9′); 1.42–1.43 (6H, s, H-1′, H-2′); 1.57–1.63 (1H, m, H-6); 1.80–1.92 (3H, m, H-6, H-8, H-8a); 1.93–2.04 (1H, m, H-4); 2.19–2.28 (3H; m; H-3, H-3a, H-9); 2.32–2.42 (1H, m, H-7); 2.51–2.61 (1H, m, H-9); 4.70 (2H, s, H-10′); 5.03 (1H, s, H-13); 5.20 (1H, s, H-13); 5.5 (1H, s, H-5′); 5.8 (1H, s, H-1′); 7.25–7.27 (1H, m, H-14′), 7.33–7.37 (4H, m, H-12′, H-13′, H-15′,H-16′). ^13^C-NMR (δ = ppm, 50 MHz): 15.5 (C-15); 19.1 (C-14); 24.4, 24.5 (C-1′,C-2′); 28.4 (C-8); 28.6 (C-7′,8′,9′); 28.8 (C-9); 35.1 (C-7); 36.1 (C-4); 39.0 (C-6); 40.1 (C-5); 42.2 (C-3); 50.3 (C-10′); 51.0 (C-6′); 62.9 (C-3′); 112.3 (C-13); 125.7 (C-1); 126.1 (C-12′,C-16′); 127.2 (C-14′); 128.8 (C-13′,C15′); 139.5 (C-11′); 149.5 (C-11); 173.0 (C-10); 173.4 (C-12); 173.9 (C-4′); 199.0 (C-2). HRMS-ES (*m*/*z*) [M + Na]^+^: Calcd. for C_30_H_42_N_2_O_3_Na: 501.3089; found: 501.3093.

Compound [**6**]: Yellow solid, m.p.: 70–71 °C. [α]_D_^20^: −90.5 (*c* 9.96; CHCl_3_). IR (KBr; υ_max_: cm^−1^): 3365, 2931, 2359, 1653, 1518, 1491, 1365, 1282, 1167, 702. ^1^H-NMR: (δ = ppm, 600 MHz): 0.93 (3H, d, *J* = 7 Hz, Me-15); 1.03 (3H, s, Me-14); 1.39–1.45 (1H, m, H-6); 1.42, 1.49 (6H, s, H-1′, H-2′); 1.64–1.68 (1H, m, H-8); 1.70–1.74 (2H, m; H-6,H-8); 2.04–2.09 (1H, m, H-4); 2.10–2.15 (1H, m, H-7); 2.17–2.22 (1H, m, H-9); 2.24–2.30 (2H, m, H-3, H-3a); 2.45–2.52 (1H, m, H-9); 4.60 (2H, d, *J* = 6 Hz, H-6′); 4.95 (1H, s, H-13); 4.98 (1H, s, H-13); 5.8 (1H, s, H-1′); 6.1 (1H, t, *J* = 5 Hz, H-5′); 7.25–7.28 (2H, m, H-15′, H-17′); 7.29–7.31 (1H, m, H-16′); 7.31–7.37 (5H, m, H-9′, H-10′, H-11′, H-14′, H-18′); 7.38–7.40 (2H, m, H-8′, H-12′). ^13^C-NMR (δ = ppm, 150 MHz): 15.4 (C-15); 19.1 (C-14); 25.4, 25.7 (C-1′,C-2′); 28.7 (C-9); 28.8 (C-8); 34.3 (C-7); 35.9 (C-4); 38.6 (C-6); 40.2 (C-5); 42.2 (C-3); 44.0 (C-6′); 62.7 (C-3′); 116.1 (C-13); 125.6 (C-1); 127.4 (C-16′); 127.8 (C-8′,C-12′); 128.2, 128.6, 128.7 (C-9′,C-10′,C-11′,C-12′,C-14′,C-18′); 131.3 (C-15′,C-17′); 138.5 (C-7′); 139.4 (C-13′); 149.0 (C-11); 171.5 (C-12); 173.7 (C-10); 174.5 (C-4′); 199.2 (C-2). HRMS-ES (*m*/*z*) [M + Na]^+^: Calcd. for C_32_H_38_N_2_O_3_Na: 521.2778; found: 521.2780.

Compound [**7**]: Yellow solid, m.p.: 70–72 °C. [α]_D_^20^: −74.7 (*c* 8.53; CHCl_3_). IR (KBr; υ_max_: cm^−1^): 3361, 2935, 2359, 1653, 1522, 1362, 1456, 1171, 698. ^1^H-NMR: (δ = ppm, 600 MHz): 0.93 (3H, d, *J* = 7 Hz, Me-15); 1.07 (3H, s, Me-14); 1.50–1.54 (1H, m, H-6); 1.50, 1.54 (6H, s, H-1′, H-2′); 1.83–1.90 (3H, m, H-6, H-8, H-8a); 1.97–2.02 (1H, m, H-4); 2.22–2.27 (3H, m, H-3, H-3a, H-9); 2.38–2.45 (1H, m, H-7); 2.52–2.59 (1H, m, H-9); 4.51 (2H; t; *J* = 5 Hz, H-6′); 4.78 (2H, d, *J* = 3 Hz, H-13′); 5.05 (1H, d, *J* = 2 Hz, H-13); 5.3 (1H, s, H-13); 5.8 (1H, s, H-1′); 6.0 (1H, t, *J* = 5 Hz, H-5′); 7.27–7.31 (2H, m, H-10′, H-17′); 7.34–7.38 (6H, m, H8′, H-9′, H-11′, H-12′, H-16′, H-18′); 7.41–7.44 (2H, m, H-15′, H-19′). ^13^C-NMR (δ = ppm, 150 MHz): 15.5 (C-15); 19.1 (C-14); 24.3, 24.6 (C-1′,C-2′); 28.4 (C-8); 28.8 (C-9); 34.9 (C-4); 35.8 (C-7); 38.7 (C-6); 40.2 (C-5); 42.1 (C-3); 43.9 (C-6′); 50.3 (C-13′); 62.5 (C-3′); 112.6 (C-13); 125.7 (C-1); 126.1 (C-15′,C-19′); 127.2, 127.4 (C-10′, C17′); 127.8, 128.7, 128.8 (C-8′, C-9′, C-11′, C-12′, C16′, C-18′); 138.5 (C-7′); 139.3 (C-14′); 149.0 (C-11); 173.2 (C-10); 173.6 (C-12); 174.7 (C-4′); 199.1 (C-2). HRMS-ES (*m*/*z*) [M + Na]^+^: Calcd. for C_33_H_40_N_2_O_3_Na: 535.2934; found: 535.2937.

Compound [**8**]: Amorphous solid, m.p.: 73–74 °C. [α]_D_^20^: −85.9 (*c* 9.66; CHCl_3_). IR (KBr; υ_max_: cm^−1^): 3385, 2927, 2360, 1653, 1541, 1456, 1363, 1169, 669. ^1^H-NMR: (δ = ppm, 600 MHz): 0.92 (3H, d, *J* = 7 Hz, Me-15); 1.00 (3H, s, Me-14); 1.12–1.23 (3H, m, H-7′/H-11′, H-9′); 1.34–1.43 (2H, m, H-8′/H-10′, H-6); 1.37, 1.40 (6H, s, H-1′, H-2′); 1.58–1.73 (7H, m, H-6, H-8, H-8a, H-8′/H-10′, H-9′); 1.92–2.10 (4H, m, H-4, H-7, H-7′/H-11′); 2.14–2.23 (1H, m, H-9); 2.23–2.36 (2H, m, H-3, H-3a); 2.37–2.56 (1H, m, H-9); 3.78 (1H; m, H-6′); 4.91 (1H, s, H-13); 4.97 (1H, s, H-13); 5.66 (1H, d, *J* = 8 Hz, H-5′); 5.78 (1H, s, H-1); 7.19–7.26 (2H, m, H-13′, H-17′); 7.27–7.34 (3H, m, H-14′, H-15′, H-16′). ^13^C-NMR: (δ = ppm, 150 MHz): 15.4 (C-15); 19.0 (C-14); 24.2, 24.4 (C-1′,C-2′); 24.9 (C-8, C-10′); 25.6 (C-9′); 28.8 (C-8, C-9); 33.0 (C-7′, C-11′); 34.2 (C-7); 36.0 (C-4); 38.7 (C-6); 40.1 (C-5); 42.2 (C-3); 48.5 (C-6′); 62.7 (C-3′); 115.6 (C-13); 125.5 (C-1); 128.1 (C-15′); 128.5 (C-14′,C-16′); 131.2 (C13′,C17′); 139.9 (C-12′); 149.3 (C-11); 171.4 (C-12); 173.6 (C-4′,C-10); 199.1 (C-2). HRMS-ES (*m*/*z*) [M + Na]^+^: Calcd. for C_31_H_42_N_2_O_3_Na: 513.3093; found: 513.3099.

Compound [**9**]: Amorphous solid, m.p.: 75–77 °C. [α]_D_^20^: −62.6 (*c* 9.96; CHCl_3_). IR (KBr; υ_max_: cm^−1^): 3423, 2931, 2852, 1655, 1522, 1387, 1179, 729. ^1^H-NMR: (δ = ppm, 600 MHz): 0.92 (3H, d, *J* = 7 Hz, Me-15); 1.04 (3H, s, Me-14); 1.16–1.27 (3H, m, H-7′/H-11′, H-9′); 1.29–1.37 (2H, m, H-8′/H-10′); 1.43, 1.45 (6H, s, H-1′, H-2′); 1.48–1.56 (1H, m, H-6); 1.555–1.634 (1H, m, H-9′); 1.692–1.777 (2H, m, H-8′/H-10′); 1.803–1.917 (3H, m; H-6, H-8, H-8a); 1.92–1.99 (3H, m, H-4, H-7′/H-11′); 2.18–2.26 (3H, m, H-3, H-3a, H-9); 2.30–2.44 (1H, m, H-7); 2.47–2.64 (1H, m, H-9); 3.74 (1H, m, H-6′); 4.70 (2H, s, H-12′); 5.0 (1H, s, H-13); 5.2 (1H, s, H-13); 5.5 (1H, d, *J* = 8 Hz, H-5′); 5.8 (1H, s, H-1); 7.27–7.32 (1H, m, H-16′); 7.34–7.39 (2H, t, *J* = 7 Hz, H-15′, H-17′); 7.41–7.48 (2H, m, H-14′, H-18′). ^13^C-NMR: (δ = ppm, 150 MHz): 15.4 (C-15); 19.1 (C-14); 24.2, 24.4 (C-1′, C-2′); 24.9 (C-8, C-10′); 25.6 (C-9′); 28.4 (C-8); 28.8 (C-9); 33.0 (C-7′, C-11′); 34.7 (C-7); 35.8 (C-4); 38.6 (C-6); 40.1 (C-5); 42.1 (C-3); 48.5 (C-6′); 50.0 (C-12′); 62.4 (C-3′); 112.3 (C-13); 125.7 (C-1); 126.1 (C-14′, C-18′); 127.2 (C-16′); 128.7 (C-15′, C-17′); 139.5 (C-7′); 149.2 (C-11); 173.2 (C-10); 173.4 (C-12); 173.8 (C-4′); 199.1 (C-2). HRMS-ES (*m*/*z*) [M + Na]^+^: Calcd. for C_32_H_44_N_2_O_3_Na: 527.3250; found: 527.3246.

Compound [**10**]: Amorphous solid, m.p.: 44–46 °C. [α]_D_^20^: −23.6 (*c* 10.81; CHCl_3_). IR (KBr; υ_max_: cm^−1^): 3429, 2926, 1655, 1493, 1363, 1228, 1171, 706. ^1^H-NMR: (δ = ppm, 600 MHz): 0.82 (3H, s, Me-14); 0.97–1.03 (1H, m, H-6); 1.03–1.09 (2H, m, H-1, H-9); 1.10 (3H, s, Me-15); 1.19 (1H, dd, *J* = 2 y 12 Hz, H-5); 1.25–1.33 (2H, m, H-2); 1.35, 1.43 (6H, s, H-1′, H-2′); 1.37–1.39 (3H, m, H-1, H3, H-9); 1.41 (9H, s, H-7′,H-8′, H-9′); 1.50–1.56 (2H, m, H-8,); 1.79–1.81 (1H, m, H-3); 1.85–1.89 (1H, m, H-6); 1.94–2.00 (1H, m, H-7); 4.92, 4.96 (2H, s, H-13); 5.8 (1H, s, H-5′); 7.26–7.29 (3H, m, H-12′, H-13′, H-14′); 7.34–7.36 (2H, m, H-11′, H-15′). ^13^C-NMR: (δ = ppm, 150 MHz): 18.6 (C-14); 20.0 (C-8); 22.8 (C-15); 25.1, 25.8 (C-1′, C-2′); 25.6 (C-6); 26.1 (C-2); 28.7 (C-7′, C-8′, C-9′); 34.5 (C-10); 40.9 (C-9); 41.9 (C-7); 42.3 (C-3); 44.3 (C-1); 51.1 (C-6′); 54.9 (C-5); 63.2 (C-3′); 72.0 (C-4); 114.4 (C-13); 128.1 (C-13′); 128.5 (C11′,C-15′); 131.4 (C-12′,C-14′); 139.6 (C-10′); 150.9 (C-11); 172.0 (C-12); 173.8 (C-4′). HRMS-ES (*m*/*z*) [M + Na]^+^: Calcd. for C_29_H_44_N_2_O_3_Na: 491.3251; found: 491.3250.

Compound [**11**]: White amorphous solid, m.p.: 70–71 °C. [α]_D_^20^: −13.2 (*c* 9.11; CHCl_3_). IR (KBr; υ_max_: cm^−1^): 3446, 2922, 1624, 1454, 1362, 1232, 1171, 908. ^1^H-NMR: (δ = ppm, 600 MHz): 0.80 (3H, s, Me-14); 0.98 (1H, m, H-9); 1.00 (3H, s, Me-15); 1.06–1.09 (1H, m, H-6); 1.09–1.13 (1H, m, H-1); 1.18 (1H, dd, *J* = 2 y 12 Hz, H-5); 1.25 (9H, s, H-7′, H-8′, H-9′); 1.26–1.29 (2H, m, H-9, H-3); 1.29, 1.32 (6H, s, H-1′, H-2′); 1.33–1.36 (1H, m, H-1); 1.37–1.40 (1H, m, H-2); 1.41-1.47 (2H, m, H-8); 1.53–1.59 (1H, m, H-2); 1.67–1.72 (1H, dd, *J* = 12 Hz, H-3);1.92–1.97 (1H, m, H-6); 2.20 (1H, m, H-7); 4.6 (2H, s, H-10′); 4.92, 5.08 (2H, s, H-13); 5.5 (1H, s, H-5′); 7.17 (1H, m, H-14′); 7.25–7.27 (4H, m, H-12′, H-13′, H-15′, H-16′). ^13^C-NMR: (δ = ppm, 150 Hz): 18.6 (C-14); 20.0 (C-8); 22.9 (C-15); 24.4, 24.6 (C-1′,C-2′); 25.5 (C-6); 26.1 (C-2); 28.6 (C-7′, C-8′, C-9′); 34.5 (C-10); 41.0 (C-9); 42.9 (C-7); 42.6 (C-3); 44.2 (C-1); 51.0 (C-6′); 54.8 (C-5); 63.0 (C-3′); 71.8 (C-4); 111.1 (C-13); 126.4 (C-12′,C-16′); 127.2 (C-14′); 128.7 (C-13′,C15′); 139.5 (C11′); 150.8 (C-11); 174.0 (C-4′); 174.1 (C-12). HRMS-ES (*m*/*z*) [M + Na]^+^: Calcd. for C_30_H_46_N_2_O_3_Na: 505.3401; found: 505.3404.

Compound [**12**]: Amorphous solid, m.p.: 61–62 °C. [α]_D_^20^: −27.5 (*c* 13.09; CHCl_3_). IR (KBr; υ_max_: cm^−1^): 3450, 2926, 1655, 1491, 1369, 1169, 700. ^1^H-NMR: (δ = ppm, 600 MHz): 0.80 (3H, s, Me-14); 0.97–1.01 (1H, m, H-6); 1.05 (3H, s, Me-15); 1.08–1.06 (1H, m, H-9); 1.10 (1H, dd, *J* = 4 y 13 Hz, H-1); 1.15 (1H, dd, *J* = 2 y 12 Hz, H-5); 1.24–1.28 (1H, m, H-2); 1.34–1.38 (3H, m, H-1, H-3, H-9); 1.44, 1.45 (6H, s, H-1′, H-2′); 1.48–1.56 (2H, m, H-8,); 1.74–1.77 (1H, m, H-3); 1.81–1.84 (1H, m, H-6); 2.01–2.05 (1H, m, H-7); 4.51, 4.58 (2H, dd, *J* = 6 Hz, H-6′); 4.94 (2H, s, H-13); 6.2 (1H, t, *J* = 6 Hz, H-5′); 7.26–7.31 (1H, m, H-10′); 7.28–7.31 (2H, m, H15′, H-17′); 7.34–7.37 (5H, m, H-9′, H-11′, H-14′, H16′, H-18′); 7.38–7.40 (2H, m, H-8′, H12′). ^13^C-NMR (δ = ppm, 150 MHz): 18.5 (C-14); 20.0 (C-8); 22.7 (C-15); 25.2, 25.6 (C-1′, C-2′); 25.9 (C-6); 26.1 (C-2); 34.4 (C-10); 40.9 (C-9); 41.8 (C-7); 42.7 (C-3); 43.9 (C-6′); 44.3 (C-1); 54.8 (C-5); 62.8 (C-3′); 72.0 (C-4); 114.8 (C-13); 127.3 (C-10′); 127.8 (C-8′,C-12′); 128.2 (C-16′); 128.5, 128.6 (C-9′, C-11′, C-14′, C-18′); 131.4 (C-15′, C-17′); 139.5 (C-13′); 150.4 (C-11); 172.2 (C-12); 174.6 (C-4′). HRMS-ES (*m*/*z*) [M + Na]^+^: Calcd for C_33_H_42_N_2_O_3_Na: 525.3091; found: 525.3093.

Compound [**13**]: Amorphous solid. m.p.: 75–76 °C. [α]_D_^20^: −15.5 (*c* 10.53; CHCl_3_). IR (KBr; υ_max_: cm^−1^): 3361, 2926, 1647, 1456, 1385, 1362, 908; 698. ^1^H-NMR: (δ = ppm, 600 MHz): 0.88 (3H, s, Me-14); 1.08–1.10 (1H, m, H-9); 1.10 (3H, s, Me-15); 1.16–1.21 (2H, m, H-1; H-6); 1.22–1.25 (1H, m, H-5); 1.33–1.37 (1H, m, H-3); 1.37–1.41 (1H, m, H-9); 1.43–1.46 (1H, m, H-1); 1.48, 1.52 (6H, s, H-1′, H-2′); 1.50–1.51 (1H, m, H-2); 1.53–1.59 (2H, m, H-8); 1.66–1.71 (1H, m, H-2); 1.76–1.80 (1H, m, H-3); 2.00–2.04 (1H, m, H-6); 2.35–2.40 (1H, m, H-7); 4.5 (2H, ddd, *J* = 6 Hz, H-6′); 4.81 (2H, s, H-13′); 5.03, 5.20 (2H, s, H-13); 6.1 (1H, t, *J* = 6 Hz, H-5′); 7.26–7.28, 7.29–7.30 (2H, m, H-10′, H-17′); 7.36–7.39 (6H, m, H-8′, H-9′, H-11′, H-12′, H-16′, H-18′); 7.41 (2H, d, *J* = 7 Hz, H-15′, H-19′). ^13^C-NMR (δ = ppm, 150 MHz): 18.6 (C-14); 20.0 (C-8); 22.7 (C-15); 24.4, 24.5 (C-1′,C-2′); 25.6 (C-6); 26.2 (C-2); 34.6 (C-10); 40.9 (C-9); 42.8 (C-7); 42.7 (C-3); 43.9 (C-6′); 44.3 (C-1); 50.5 (C-13′); 54.8 (C-5); 62.7 (C-3′); 72.0 (C-4); 111.1 (C-13); 126.4 (C-15′,C-19′); 127.2, 127.3 (C-10′,C-17′); 127.9 (C-8′yC-12′); 128.6, 128.7 (C-9′,C-11′,C-16′,C-18′); 138.7 (C-7′); 139.3 (C-14′); 150.3 (C-11); 174.2 (C-12); 174.9 (C-4′). HRMS-ES (*m*/*z*) [M + Na]^+^: Calcd. for C_33_H_44_N_2_O_3_Na: 539.3246; found: 539.3250.

Compound [**14**]: Amorphous solid, m.p.: 78–79 °C. [α]_D_^20^: −17.8 (*c* 9.3; CHCl_3_). IR (KBr; υ_max_: cm^−1^): 3448, 2931, 2361, 1655, 1543, 1460, 1176. ^1^H-NMR: (δ = ppm, 600 MHz): 0.79 (3H, s, Me-14); 0.96–1.02 (1H, m, H-6); 1.03–1.10 (2H, m, H-9, H-1); 1.07 (3H, s, Me-15); 1.16–1.27 (5H, m, H-5, H-7′, H-9′, H-9′, H-11′,); 1.30–1.38 (5H, m, H-1, H-3, H-9, H-8′, H-10′); 1.34, 1.42 (6H, s, H-1′, H-2′); 1.40–1.447 (2H, m, H-2); 1.54–1.58 (2H, m, H-8); 1.67–1.74 (2H, m, H-8′,H-10′); 1.76–1.82 (2H, m, H-6,H-3); 1.93–2.20 (3H, m, H-7; H-7′-H-11′); 3.70–3.85 (1H, m, H-6′); 4.91 (1H, s, H-13); 4.96 (1H, s, H-13); 5.7475 (1H, d, *J* = 8 Hz, H-5′); 7.23–7.28 (2H, m, H-14′, H-16′); 7.30–7.36 (3H, m, H-13′, H-15′, H-17′). ^13^C-NMR (δ = ppm, 150 MHz): 18.5 (C-14); 19.9 (C-8); 22.7 (C-15); 24.8 (C-8′/C10′); 25.05, 25.73 (C-1′,C-2′); 25.6 (C-6)*; 25.7 (C-9′); 26.2 (C-2); 32.9, 33.0 (C-7′,C11′); 34.4 (C-10); 40.8 (C-9); 42.6 (C-7); 42.6 (C-3); 44.3 (C-1); 48.4 (C-6′); 54.8 (C-5); 62.7 (C-3′); 71.9 (C-4); 114.5 (C-13); 128.1 (C-13′,C-17′); 128.4 (C-15′); 131.5 (C-14′y C-16′); 139.6 (C-12′); 150.7 (C-11); 172.0 (C-12); 173.7 (C-4′). HRMS-ES (*m*/*z*) [M + Na]^+^: Calcd. for C_31_H_46_N_2_O_3_Na: 517.3405; found: 517.3406.

Compound [**15**]: Amorphous solid, m.p.: 57–59 °C. [α]_D_^20^: −13.7 (*c* 8.35; CHCl_3_). IR (KBr; υ_max_: cm^−1^): 3421, 2926, 2360, 1653, 1541, 1458, 1171. ^1^H-NMR: (δ = ppm, 600 MHz): 0.87 (3H, s, Me-14); 1.10–1.11 (1H, m, H-9); 1.10 (3H, s, Me-15); 1.14–1.20 (4H, m, H-6, H-7′, H-9′, H-11′); 1.21–1.24 (1H, m, H-1); 1.27–1.29 (1H, m, H-5); 1.37–1.40 (3H, m, H-3, H-9, H-8′/H-10′); 1.33–1.37 (1H, m, H-8′/H-10′); 1.43–1.45 (1H, m, H-1); 1.44, 1.44 (6H,, H-2′); 1.46–1.50 (1H, m, H-2); 1.53–1.57 (2H, m, H-8); 1.59–1.62 (1H, m, H-9′); 1.66–1.73 (3H, m, H-2, H-8′/H-10′); 1.78–1.81 (1H, m, H-3); 1.93–1.99 (2H, m, H-7′, H-11′), 1.99–2.03 (1H, m, H-6); 2.29–2.42 (1H, m, H-7); 3.74 (1H, m, H-6′); 4.74 (2H, d, *J* = 5 Hz, H-12); 5.02 (1H, s, H-13); 5.19 (1H, s, H-13); 5.63 (1H, d, *J* = 8 Hz, H-5′); 7.29–7.27 (1H, m, H-16′); 7.37 (2H, t, *J* = 7 Hz, H-15′, H-17′); 7.42 (2H, d, *J* = 7 Hz, H-14′, H-18′). ^13^C-NMR (δ = ppm, 150 MHz): 18.6 (C-14); 20.0 (C-8); 22.8 (C-15); 24.3, 24.5 (C-1′,C-2′); 24.83, 24.9 (C-8′,C-10′); 25.5 (C-6); 25.7 (C-9′); 26.2 (C-2); 32.9, 30.0 (C-7′,C11′); 34.6 (C-10); 41.0 (C-9); 42.6 (C-7); 42.7 (C-3); 44.3 (C-1); 48.4 (C-6′); 50.2 (C-12′); 54.8 (C-5); 62.6 (C-3′); 71.9 (C-4); 111.2 (C-13); 126.4 (C-14′,C-18′); 127.1 (C-16′); 128.6 (C-15′y C-17′); 139.5 (C-13′); 150.5 (C-11); 174.0 (C-4′); 174.1 (C-12′). HRMS-ES (*m*/*z*) [M + Na]^+^: Calcd. for C_32_H_48_N_2_O_3_Na: 531.3565; found: 531.3563.

Compound [**16**]: Colorless oil. [α]_D_^20^: −10.2 (*c* 6.17; CHCl_3_). IR (KBr; υ_max_: cm^−1^): 3294, 2926, 2854, 1713, 1468, 1261, 1093, 802. ^1^H-NMR (δ = ppm, 600 MHz): 0.88 (3H, s, Me-14); 0.88 (3H, t, *J* = 8.0 Hz Me-12′); 1.04–1.12 (1H, m, H-9a); 1.10 (3H, s, Me-15); 1.14–1.21 (2H, m, H-1a, H-2a); 1.21–1.25 (1H, m, H-5); 1.25–1.35 (14H, m, H-5′, H-6′, H-7′, H-8′, H-9′, H-10′, H-11′); 1.35-1.37 (1H, m, H-3a); 1.37–1.40 (1H, m, H-9b); 1.40–1.44 (2H, m, H-1b, H-6a); 1.50–1.60 (3H, m, H-6b, H-8a, H-8b); 1.63–1.70 (2H, m, H-4′); 1.78–1.83 (1H, m, H-3b); 1.86–1.94 (2H, m, H-2b, H-7); 2.7 (2H, t, *J* = 8.8 Hz, H-3′); 4.83, 5.08 (2H, s, H-13a, H-13b); 4.95 (2H, c, *J* = 9.0 Hz, H-12); 7.26 (1H, s, H-1′). ^13^C-NMR (δ = ppm, 50 MHz): 14.1 (C-12′); 18.2 (C-6′); 18.7 (C-15); 20.1 (C-8); 22.6 (C-14); 25.2 (C-3′); 26.1 (C-2); 27.0 (C-6); 28.4 (C-4′); 27.9 (C-5′); 34.6 (C-10); 40.9 (C-9); 42.4 (C-7); 43.5 (C-3); 44.4 (C-1); 54.2 (C-12); 54.8 (C-5); 64.5 (C-8′); 72.1 (C-4); 84.4 (C-7′); 112.8 (C-13); 120.8 (C-1′); 148.1 (C-2′); 148.7 (C-11). HRMS (*m*/*z*) [M]^+^: Calcd. for C_27_H_47_N_3_O: 429.3719; found 429.3716.

Compound [**17**]: Colorless oil. [α]_D_^20^: −30.8 (*c* 1.59; CHCl_3_). IR (KBr; υ_max_: cm^−1^): 2962, 1741, 1477, 1261, 1089, 798. ^1^H-NMR: (δ = ppm, 600 MHz): 0.87 (3H, s, Me-14); 1.02–1.08 (1H, m, H-9); 1.09 (3H, s, Me-15); 1.41.24 (3H, m, H-1, H-2, H-5); 1.32–1.37 (1H, m, H-3); 1.37–1.46 (3H, m, H-1, H-6, H-9); 1.52–1.61 (5H, m, H-6, H-8, H-8a, H-5′, H-5′a); 1.74–1.83 (3H, m, H-3, H-4′, H-4′a); 1.83–1.91 (2H, m, H-2, H-7); 1.94 (1H, s, H-8′); 2.2 (2H, t, *J* = 11 Hz, H-6′); 2.7 (2H, t, *J* = 9 Hz, H-3′); 4.8 (1H, s, H-13); 4.94 (2H, c, *J* = 15 Hz, H-12); 5.10 (1H, s, H-13); 7.26 (1H, s, H-1′). ^13^C-NMR (δ = ppm, 150 MHz): 18.2 (C-6′); 18.7 (C-15); 20.1 (C-8); 22.6 (C-14); 25.2 (C-3′); 26.1 (C-2); 27.0 (C-6); 28.4 (C-4′); 27.9 (C-5′); 34.6 (C-10); 40.9 (C-9); 42.4 (C-7); 43.5 (C-3); 44.4 (C-1); 54.2 (C-12); 54.8 (C-5); 64.5 (C-8′); 72.1 (C-4); 84.4 (C-7′); 112.8 (C-13); 120.8 (C-1′); 148.1 (C-2′); 148.7 (C-11). HRMS (*m*/*z*) [M]^+^: Calcd. for C_22_H_32_N_3_O: 355.2624; found 355.2646.

Compound [**18**]: Colorless oil. [α]_D_^20^: −20.5 (*c* 4.60; CHCl_3_). IR (KBr; υ_max_: cm^−1^): 3477, 3330, 2927, 1648, 1467, 1172, 763. ^1^H-NMR: (δ = ppm, 600 MHz): 0.86 (3H, s, Me-14); 1.04–1.09 (1H, m, H-9); 1.07 (3H, s, Me-15); 1.12–1.173 (3H, m, H-1, H-2, H-5); 1.29–1.35 (1H, m, H-3); 1.36–1.42 (3H, m, H-1, H-6, H-9); 1.49–1.55 (3H, m, H-6, H-8, H-8a); 1.75–1.81 (1H, m, H-3); 1.82–1.88 (2H, m, H-2, H-7); 4.09 (2H, s, H-3′); 4.8 (1H, s, H-13); 4.92 (2H, c, *J* = 14 Hz, H-12); 5.04 (1H, s, H-13); 7.15 (1H, s, H-1′); 7.22 (1H, m, H-7′); 7.22–7.25 (2H, m, H-5′, H-9′); 7.29 (2H, m, H-6′, H-8′). ^13^C-NMR (δ = ppm, 150 MHz): 18.8 (C-14); 20.2 (C-8); 22.8 (C-15); 26.3 (C-2); 27.1 (C-6); 32.5 (C-3′); 34.7 (C-10); 41.0 (C-9); 42.6 (C-7); 43.7 (C-3); 44.6 (C-1); 54.4 (C-12); 55.0 (C-5); 72.2 (C-4); 113.0 (C-13); 121.8 (C-1′); 126.2 (C-7′); 128.7, 128.8 (C-5′ y 9′,C-6′ y 8′); 139.3 (C-4′); 148.1 (C-2′); 148.6 (C-11). HRMS (*m*/*z*) [M]^+^: Calcd. for C_24_H_332_N_3_O: 379.2604; found: 379.2619.

Compound [**19**]: Amorphous white solid, m.p.: 100–101 °C. [α]_D_^20^: −6.0 (*c* 5.41; CHCl_3_). IR (KBr; υ_max_: cm^−1^): 3475, 3338, 2927, 2357, 1647, 1466, 1173, 908. ^1^H-NMR: (δ = ppm, 200 MHz) 0.88 (3H, s, Me-14); 1.06–1.13 (1H, m, H-9); 1.10 (3H, s, Me-15); 1.15–1.24 (3H, m, H-1, H-2, H-5); 1.25–1.32 (1H, m, H-3); 1.32–1.43 (3H, m, H-1, H-6, H-9); 1.53–1.64 (3H, m, H-6, H-8, H-8a); 1.74–1.84 (1H, m, H-3); 1.88–2.03 (2H, m, H-2, H-7); 4.92 (1H, s, H-13); 5.03 (2H, s, H-12); 5.13 (1H, s, H-13); 7.29–7.37 (1H, m, H-6′); 7.37–7.48 (2H, m, H-5′, H-7′); 7.81–7.88 (2H, m, H-4′, H-8′); 7.74 (1H, s, H-1′). ^13^C-NMR (δ = ppm, 50 MHz): 18.7 (C-14); 20.1 (C-8); 22.5 (C-15); 26.1 (C-2); 27.0 (C-6); 34.6 (C-10); 40.9 (C-9); 42.5 (C-7); 43.6 (C-3); 44.4 (C-1); 54.4 (C-12); 54.9 (C-5); 72.1 (C-4); 113.1 (C-13); 119.6 (C-1′); 125.7 (C-4′, C-8′); 128.1 (C-6′); 128.8 (C-5′, C-7′); 130.7 (C-3′); 148.0 (C-2′); 148.6 (C-11). HRMS-ES (*m*/*z*) [M + Na]^+^: Calcd. for C_23_H_31_N_3_ONa: 388.2369; found: 388.2365.

### 3.5. Cells, Culture and Plating

The following human solid tumor cell lines were used in this study: HBL-100 (breast), HeLa (cervix), SW1573 (non-small cell lung), T-47D (breast), A549 (lung), and WiDr (colon). These cell lines were a kind gift from Prof. G. J. Peters (VU Medical Center, Amsterdam, The Netherlands). Cells were maintained in 25 cm^2^ culture flasks in RPMI 1640 supplemented with 5% heat-inactivated fetal calf serum and 2 mM L-glutamine in a 37 °C, 5% CO_2_, 95% humidified air incubator. Exponentially growing cells were trypsinized and resuspended in antibiotic containing medium (100 units penicillin G and 0.1 mg of streptomycin per mL). Single cell suspensions displaying > 97% viability by trypan blue dye exclusion test were subsequently counted. After counting, dilutions were made to give the appropriate cell densities for inoculation onto 96-well microtiter plates. Cells were inoculated in a volume of 100 μL per well at densities of 5000 (WiDr and T-47D), and 2500 (A549, HeLa, SW1573 and HBL-100) cells per well, based on their doubling times.

### 3.6. Antiproliferative Tests

Chemosensitivity tests were performed using the SRB assay of the NCI with slight modifications. Briefly, pure compounds were initially dissolved in DMSO at 400 times the desired final maximum test concentration. Control cells were exposed to an equivalent concentration of DMSO (0.25% *v*/*v*, negative control). Each agent was tested in triplicates at different dilutions in the range 1–100 μM. Drug treatment started on day 1 after plating. Drug incubation periods were 48 h, after which cells were precipitated with 25 μL of ice-cold 50% (*w*/*v*) trichloroacetic acid and fixed for 60 min at 4 °C. Then, the SRB assay was performed. The optical density (OD) of each well was measured at 530 nm, using BioTek’s PowerWave XS Absorbance Microplate Reader. Values were corrected for background OD from wells containing only culture medium. The percentage growth (PG) was calculated with respect to untreated control cells (C) at each level of drug concentrations based on the difference in OD at the start time (T_0_) and at the end of drug exposure (T), according to NCI formulas. Therefore, if T is greater than or equal to T_0_, the calculation is 100 × ((T − T0)/(C − T_0_)). If T is lower than T_0_, denoting cell death, the calculation is 100 × ((T − T_0_)/(T_0_)). The effect is defined as the growth percentage, where 50% growth inhibition (GI_50_) represents the concentration at which PG is +50. Based on these calculations, a PG value of 0 corresponds to the number of cells present at the beginning of drug exposure, while negative PG values denote net cell death.

### 3.7. Parasites

*Trypanosoma cruzi* epimastigotes (RA strain, from discrete typing unit (DTU) VI) were grown in a biphasic medium. Cultures were maintained by weekly passages at 28 °C [18].

### 3.8. In Vitro Anti-Trypanosoma cruzi Assay

The growth inhibition of *T. cruzi* epimastigotes was evaluated by a (^3^H) thymidine uptake assay as previously described [19]. Natural sesquiterpenes and derivatives were evaluated at concentrations ranging from 1.5 to 50 µg/mL. Benznidazole (Active Pharmaceutical Ingredient) was used as positive control (Elea). The percentage of inhibition was calculated as 100 = {((cpm of treated parasites)/(cpm of untreated parasites)) × 100}.

### 3.9. Cytotoxicity on Primary Cell Culture

The cytotoxicity of the compounds was evaluated on splenocytes obtained from Balb/c mouse (1.5 × 10^5^) [20,21] and incubated with different drug dilutions (5–200 μg/mL) as previously described [9]. Cell death was determined by flow cytometry using a BD FACSaria II cytometer. Cells incubated only with drug vehicle were used as 100% viability control, and death percentage was calculated according to the following formula:

$$\mathrm{Death}\left(\%\right)=\left[1-\frac{(\%{\mathrm{PI}}^{-}\mathrm{cells}{)}_{\mathrm{drug}-\mathrm{treated}}}{(\%{\mathrm{PI}}^{-}\mathrm{cells}{)}_{100\%\;\mathrm{viability}\;\mathrm{control}}}\right]\;\times \;100$$

The concentration capable of causing 50% cell death (CC_50_) was determined using a non-linear regression approach.

### 3.10. Statistical Analysis

The results are presented as means ± SD. GraphPad Prism 5.0 software (GraphPad Software Inc., San Diego, CA, USA) was employed to carry out calculations. The results account for three to four independent experiments.

## 4. Conclusions

In this investigation, sesquiterpenes were the starting material for their transformation into various oxygenated and oxo-nitrogenated derivatives by chemical reactions directed at primary hydroxyls or acid groups. Our strategy was to obtain new derivatives, including functionalities such as diamides and 1,2,3-triazoles. The natural sesquiterpenes tessaric acid, ilicic acid, and ilicic alcohol did not show antiproliferative activity nor trypanocidal activity, but most of the oxonitrogenated derivatives showed activity against the solid tumor cell lines evaluated and against *T. cruzi*. The results obtained showed that from abundant and easily accessible natural products, the introduction of new functional groups improves their activity, so they can be used as scaffolds for the synthesis of new active molecules.

## Figures and Tables

**Table 1:** Antiproliferative activity (GI_50_, µM) of the natural sesquiterpenes **1**–**3** and their derivatives **4**–**19** against human solid tumor cells.

Compound	Cell Line					
	A549	HBL-100	HeLa	SW1573	T-47D	WiDr
**1**	n.e.	>100	>100	>100	>100	>100
**2**	n.e.	>100	>100	>100	>100	>100
**3**	n.e.	>100	>100	>100	>100	>100
**4**	68 (±0.12)	>100	68 (±9.5)	57 (±5.7)	95 (±6.6)	98 (±3.5)
**5**	>100	>100	>100	>100	>100	>100
**6**	33 (±5.2)	61 (±0.11)	36 (±6.5)	40 (±7.4)	30 (±4.8)	44 (±2.8)
**7**	27 (±0.33)	41 (±0.14)	19 (±1.7)	30 (±0.25)	22 (±4.4)	37 (±3.6)
**8**	27 (±1.7)	37 (±1.7)	31 (±1.7)	30 (±1.7)	27 (±1.7)	30 (±1.7)
**9**	28 (±0.29)	47 (±0.75)	24 (±0.56)	30 (±0.29)	21 (±0.22)	39 (±0.29)
**10**	31 (±5.5)	64 (±1.5)	37 (±5.3)	50 (±1.0)	36 (±5.3)	45 (±5.8)
**11**	20 (±3.8)	33 (±3.2)	19 (±3.3)	33 (±2.4)	23 (±2.3)	29 (±3.1)
**12**	22 (±2.4)	20 (±1.5)	17 (±5.6)	27 (±7.4)	20 (±2.4)	24 (±5.4)
**13**	5.3 (±0.32)	13 (±2.2)	8.0 (±0.1)	11 (±0.8)	11 (±1.0)	11 (±1.7)
**14**	22 (±4.0)	20 (±3.2)	17 (±1.8)	27 (±5.6)	23 (±2.6)	20 (±4.7)
**15**	13 (±2.1)	19 (±1.6)	14 (±1.4)	17 (±2.0)	19 (±1.0)	18 (±1.7)
**16**	n.e.	n.e.	13 (±2.2)	n.e.	17 (±1.1)	12 (±1.5)
**17**	n.e.	n.e.	31 (±2.1)	n.e.	29 (±1.8)	31 (±9.8)
**18**	n.e.	n.e.	>100	n.e.	44 (±8.2)	39 (±1.2)
**19**	n.e.	n.e.	79 (±5.0)	n.e.	61 (±3.2)	41 (±0.2)
**Cisplatin**	4.9 (±0.2)	1.9 (±0.2)	1.8 (±0.5)	2.7 (±0.4)	17 (±0.5)	23 (±4.3)

n.e.: not evaluated.

**Table 2:** Cytotoxic activity of the natural sesquiterpenes and its derivatives on spleen cells (CC_50_, µM) and selectivity indexes (SI) on tumor cells.

Compound	Splenocytes	Cell Lines					
		A549	HBL-100	HeLa	SW1573	T-47D	WiDr
**1**	329.4 (±12.9)	n.d.	n.d.	n.d.	n.d.	n.d.	n.d.
**2**	>839	n.d.	n.d.	n.d.	n.d.	n.d.	n.d.
**3**	>792.6	n.d.	n.d.	n.d.	n.d.	n.d.	n.d.
**4**	17.3 (±1.1)	0.25	n.d.	0.3	0.3	0.2	0.2
**5**	44.7 (±6.7)	n.d.	n.d.	n.d.	n.d.	n.d.	n.d.
**6**	103.6 (±0.8)	3.1	1.7	2.9	2.6	3.5	2.4
**7**	38.1 (±7.8)	1.4	0.9	2.0	1.3	1.7	1.0
**8**	19.8 (±2.0)	0.7	0.5	0.6	0.7	0.7	0.7
**9**	76.5 (±15.9)	2.7	1.6	3.2	2.6	3.6	2.0
**10**	78.2 (±5.3)	2.5	1.2	2.1	1.6	2.2	1.7
**11**	333.5 (±4.2)	16.7	10.1	17.6	10.1	14.5	11.5
**12**	64.3 (±8.5)	2.9	3.2	3.8	2.4	3.2	2.7
**13**	27.4 (±2.8)	5.2	2.1	3.4	2.5	2.5	2.5
**14**	>404.8	>18.4	>20.2	>24	>20.2	>17	>20
**15**	21.5 (±10.2)	2.5	1.5	2.2	1.5	1.7	1.7
**16**	82.4 (±0.02)	n.d.	n.d.	6.3	n.d.	4.8	6.8
**17**	40.4 (±10.4)	n.d.	n.d.	1.3	n.d.	1.4	1.3
**18**	62.2 (±13.7)	n.d.	n.d.	n.d.	n.d.	1.4	1.6
**19**	186.8 (±0.01)	n.d.	n.d.	2.4	n.d.	3.1	4.6

n.d.: not determined.

**Table 3:** Anti-*Trypanosoma cruzi* activity of the natural and semisynthetic sesquiterpenes and selectivity indexes. Results are expressed as 50% inhibitory concentration (IC_50_) ± SD.

Compound	IC_50_ (µM)	Selectivity Index
**1**	>201.4	n.d.
**2**	>198.1	n.d.
**3**	156.5 (±4.81)	n.d.
**4**	16.3 (±1.36)	1.1
**5**	9.3 (±0.89)	4.8
**6**	>100.3	n.d.
**7**	4.7 (±0.16)	8.0
**8**	63.2 (±2.00)	0.3
**9**	45.1 (±8.24)	1.7
**10**	19.6 (±0.92)	4.0
**11**	26.1 (±1.67)	12.6
**12**	8.0 (±0.4)	8.0
**13**	59.6 (±6.95)	0.5
**14**	>101.1	n.d.
**15**	22.4 (±0.91)	1.0
**16**	8.8 (±0.24)	9.4
**17**	14.8 (±0.69)	2.7
**18**	30.2 (±5.22)	2.1
**19**	48.2 (±5.40)	3.9

n.d.: not determined.

## Data Availability

Not applicable.

## Supplementary Materials

The following supporting information can be downloaded at: https://www.mdpi.com/article/10.3390/molecules27103067/s1, Figure S1. ^1^H-NMR of **1**; Figure S2. ^13^C-NMR of **1**; Figure S3 ^1^H-NMR of **2**; Figure S4 ^13^C-NMR of **2**; Figure S5 ^1^H-NMR of **3**; Figure S6 ^13^C-NMR of **3**; Figure S7 ^1^H-NMR of **4**; Figure S8 ^13^C- MR of **4**; Figure S9 COSY of **4**; Figure S10 HSQC of **4**; Figure S11 HMBC of **4**; Figure S12 ^1^H-NMR of **5**; Figure S13 ^13^C-NMR of **5**; Figure S14 COSY of **5**; Figure S15 HSQC of **5**; Figure S16 HMBC of **5**; Figure S17 ^1^H-NMR of **6**; Figure S18 ^13^C-NMR of **6**; Figure S19 COSY of **6**; Figure S20 HSQC of **6**; Figure S21 HMBC of **6**; Figure S22 ^1^H-NMR of **7**; Figure S23 ^13^C-NMR of **7**; Figure S24 COSY of **7**; Figure S25 HSQC of **7**; Figure S26 HMBC of **7**; Figure S27 ^1^H-NMR of **8**; Figure S28 ^13^C-NMR of **8**; Figure S29 COSY of **8**; Figure S30 HSQC of **8**; Figure S31 HSQC of **8**; Figure S32 ^1^H-NMR of **9**; Figure S33 ^13^C-NMR of **9**; Figure S34COSY of **9**; Figure S35 HSQC of **9**; Figure S36 HMBC of **9**; Figure S37 ^1^H-NMR of **10**; Figure S38 ^13^C-NMR of **10**; Figure S39 COSY of **10**; Figure S40 HSQC of **10**; Figure S41 HMBC of **10**; Figure S42 ^1^H-NMR of **11**; Figure S43 ^13^C-NMR of **11**; Figure S44 COSY of **11**; Figure S45 HSQC of **11**; Figure S46 HMBC of **11**; Figure S47 ^1^H-NMR of **12**; Figure S48 ^13^C-NMR of **12**; Figure S49 COSY of **12**; Figure S50 HSQC of **12**; Figure S51 HMBC of **12**; Figure S52 ^1^H-NMR of **13**; Figure S53 ^13^C-NMR of **13**; Figure S54 COSY of **13**; Figure S55 HSQC of **13**; Figure S56 HMBC of **13**; Figure S57 ^1^H-NMR of **14**; Figure S58 ^13^C-NMR of **14**; Figure S59 COSY of **14**; Figure S60 HSQC of **14**; Figure S61 HMBC of **14** Figure S62 ^1^H-NMR of **15**; Figure S63 ^13^C-NMR of **15**; Figure S64 COSY of **15**; Figure S65 HSQC of **15**; Figure S66 HMBC of **15**; Figure S671H-MR of **16**; Figure S68 ^13^C-NMR of **16**; Figure S69 COSY of **16**; Figure S70 HSQC of **16**; Figure S71 HMBC of **16**; Figure S72 ^1^H-NMR of **17**; Figure S73 ^13^C-NMR of **17**; Figure S74 COSY of **17**; Figure S75 HSQC of **17**; Figure S76 HMBC of **17**; Figure S77 ^1^H-NMR of **18**; Figure S78 ^13^C-NMR of **18**; Figure S79 COSY of **18**; Figure S80 HSQC of **18**; Figure S81 HMBC of **18**; Figure S82 ^1^H-NMR of **19**; Figure S83 ^13^C-NMR of **19**; Figure S84 COSY of **19**; Figure S85 HSQC of **19**; Figure S86 HMBC of **19**; Figure S87 HRMS-ES of **1**; Figure S88 HRMS-ES of **2**; Figure S89 HRMS-ES of **3**; Figure S90 HRMS-ES of **4**; Figure S91 HRMS-ES of **5**; Figure S92 HRMS-ES of **6**; Figure S93 HRMS-ES of **7**; Figure S94 HRMS-ES of **8**; Figure S95 HRMS-ES of **9**; Figure S96 HRMS-ES of **10**; Figure S97 HRMS-ES of **11**; Figure S98 HRMS-ES of **12**; Figure S99 HRMS-ES of **13**; Figure S100 HRMS-ES of **14**; Figure S101 HRMS-ES of **15**; Figure S102 HRMS-ES of **16**; Figure S103 HRMS-ES of **17**; Figure S104 HRMS-ES of **18**; Figure S105 HRMS-ES of **19**.

## Sample Availability

Samples of the compounds are available from the authors.
