# Supplementary material for: Oxonitrogenated Derivatives of Eremophilans and Eudesmans: Antiproliferative and Anti-Trypanosoma cruzi Activity
Source: Molecules. 2022 May 10;27(10):3067. doi: 10.3390/molecules27103067 (PMC9143450; doi:10.3390/molecules27103067)
Supplement: Supplementary file 1 [file molecules-27-03067-s001.zip › molecules-1685839-supplementary.pdf]

Supplementary Materials

Oxonitrogenated Derivatives of Eremophilans and Eudesmans: Antiproliferative and Anti-*Trypanosoma cruzi* Activity

Table of contents

1.	NMR spectra of natural sesquiterpene, tessaric acid (**1**), ilicic acid (**2**) and ilicic alcohol (**3**)	Pages S2–S4
2.	NMR spectra of tessaric acic derivatives (**4-9**)	Pages S5–S19
3.	NMR spectra of ilicic acid derivatives (**10-15**)	Pages S20–S34.
4.	NMR spectra of ilicic alcohol derivative (**16-19**)	Pages S35-S44
5.	HRMS-ES of compounds **1-19**	Pages S45–S54

1. NMR spectra of natural sesquiterpene tessaric acid (1), ilicic acid (2) and ilicic alcohol (3)

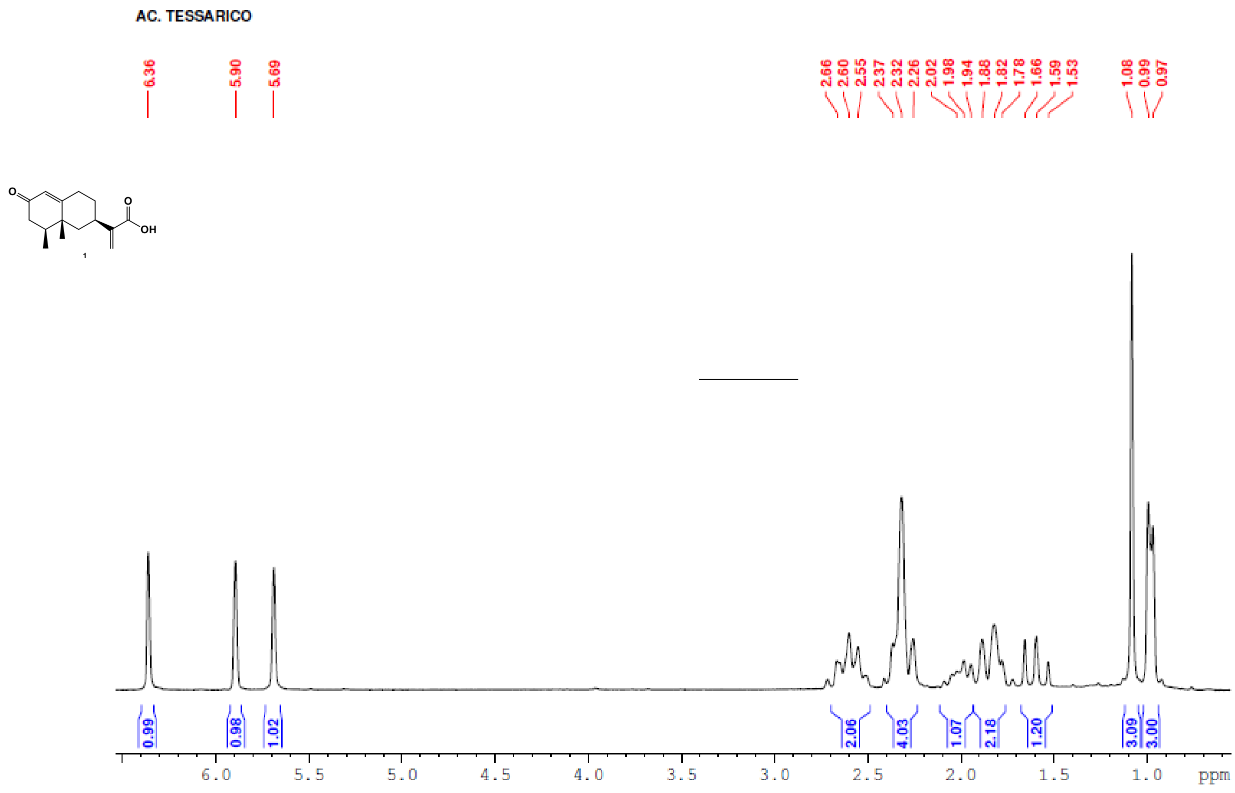

Figure S1. ^{1}H-NMR of **1.**

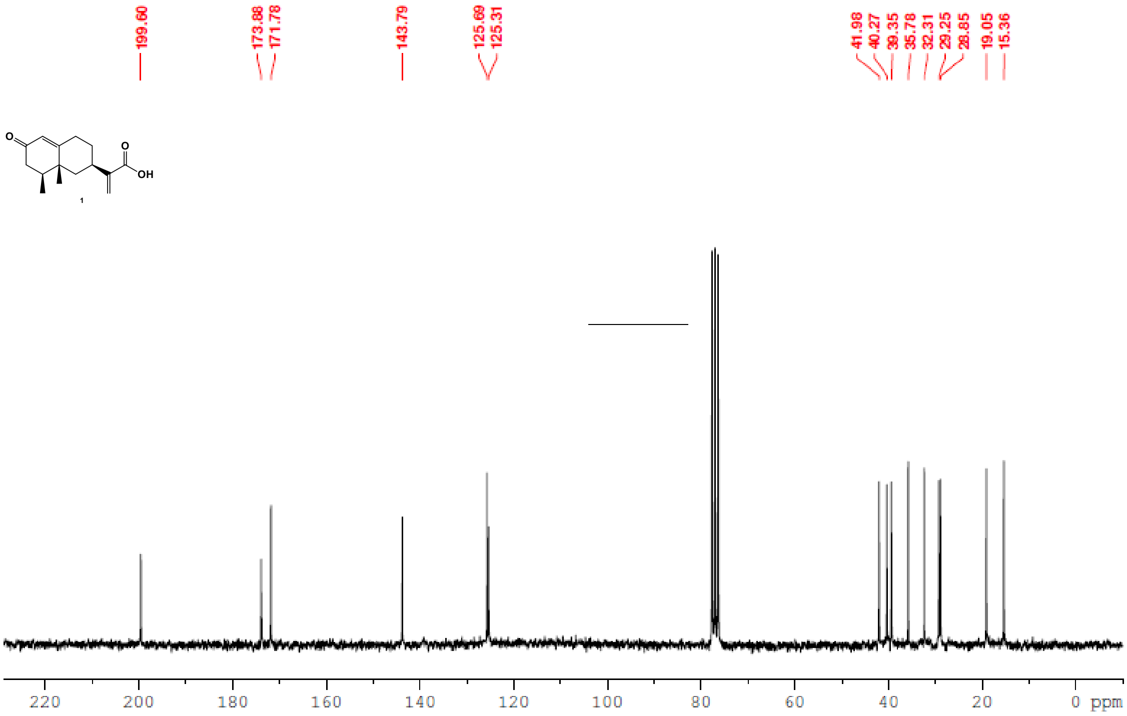

Figure S2. ^{13}C-NMR of **1.**

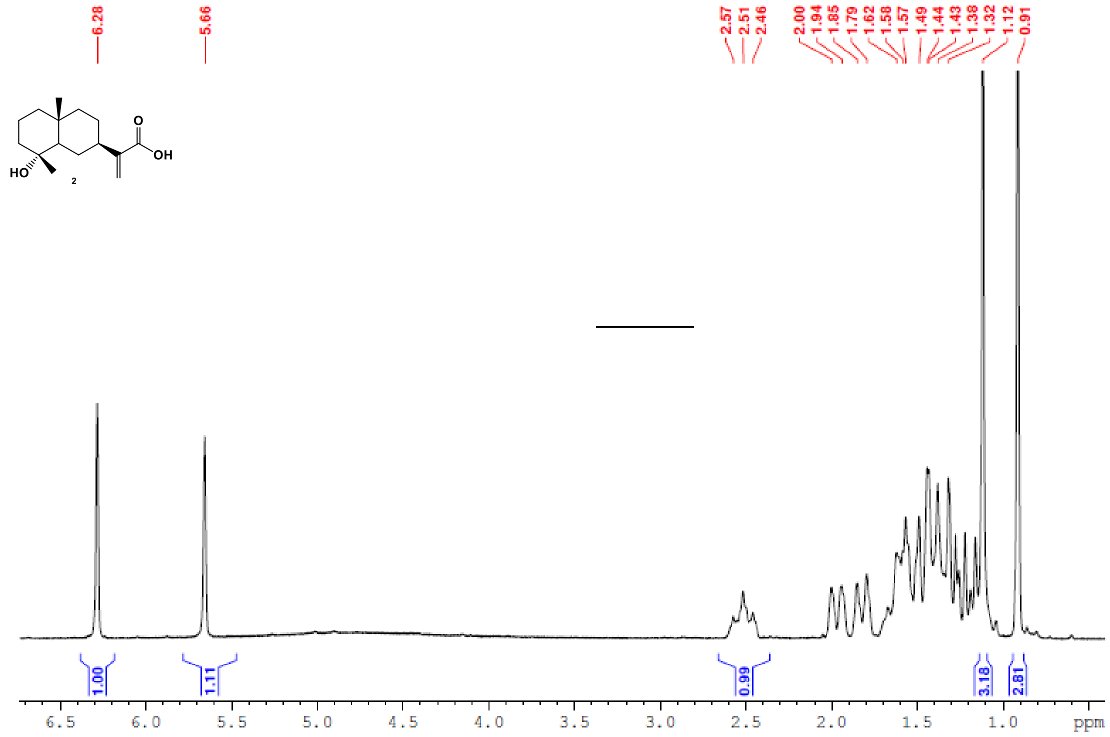

Figure S3. ^{1}H-NMR of **2.**

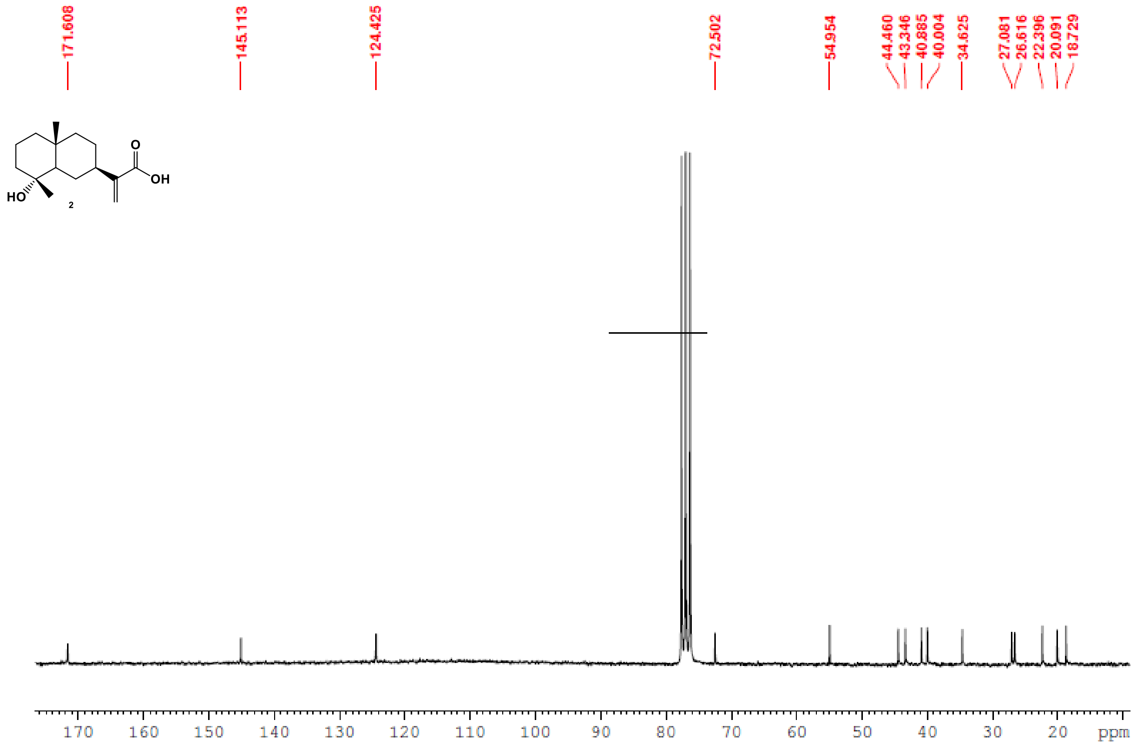

Figure S4. ^{13}C-NMR of **2.**

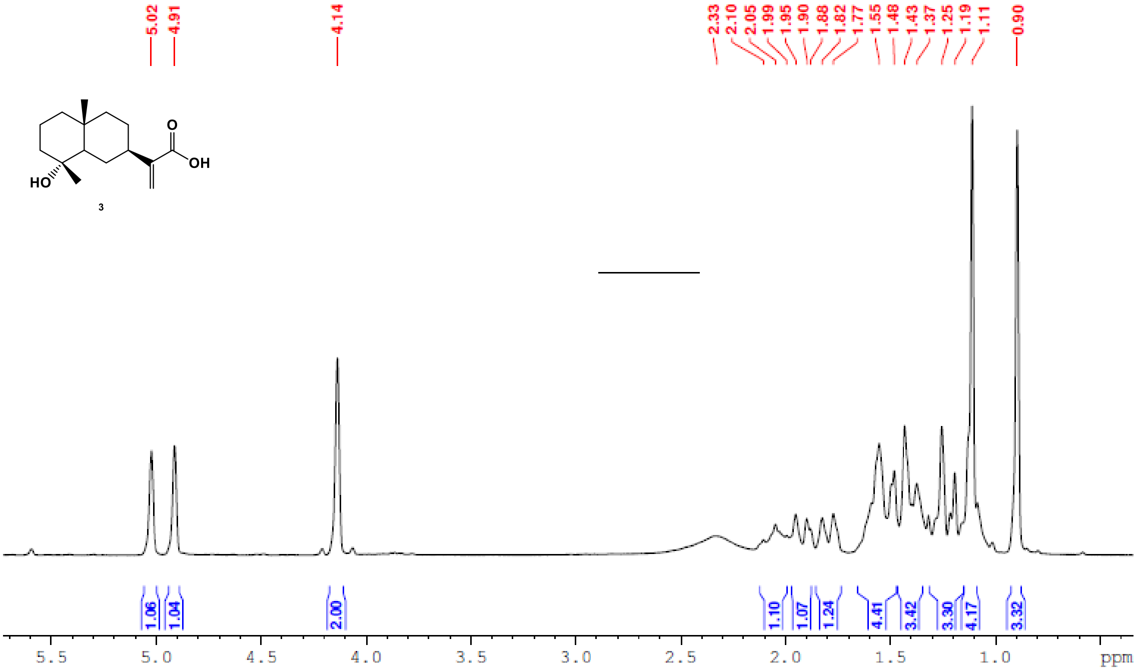

Figure S5. ^{1}H-NMR of **3.**

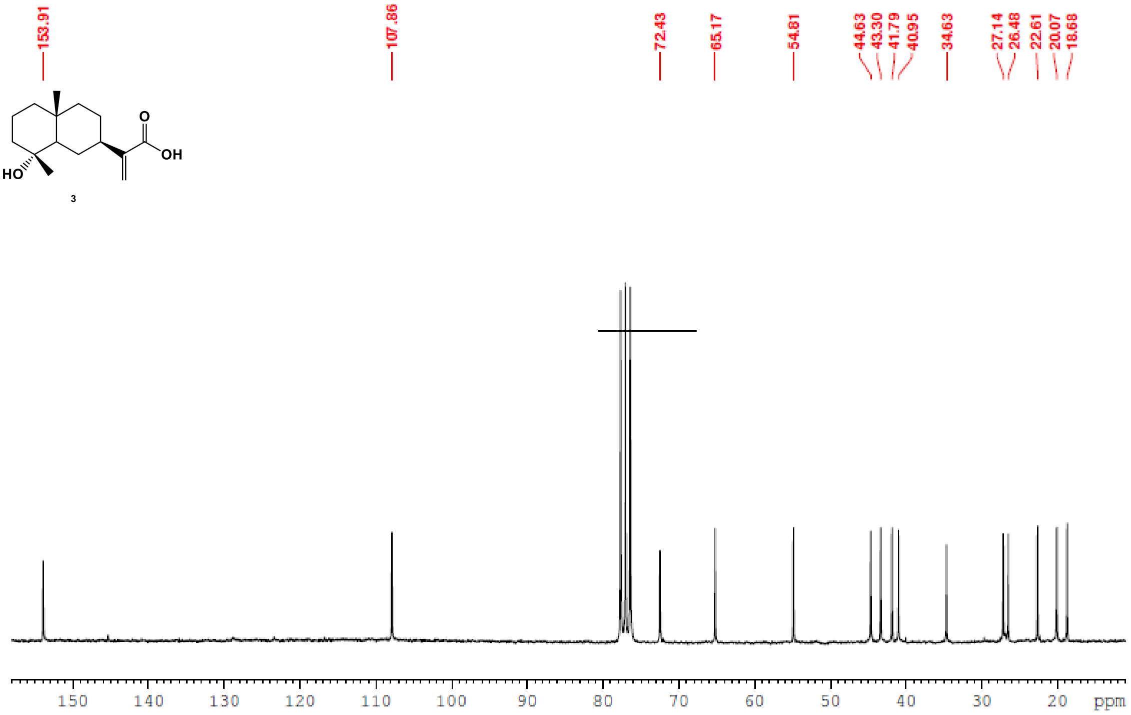

Figure S6. ^{13}C-NMR of **3.**

2. NMR spectra of tessaric acid derivatives

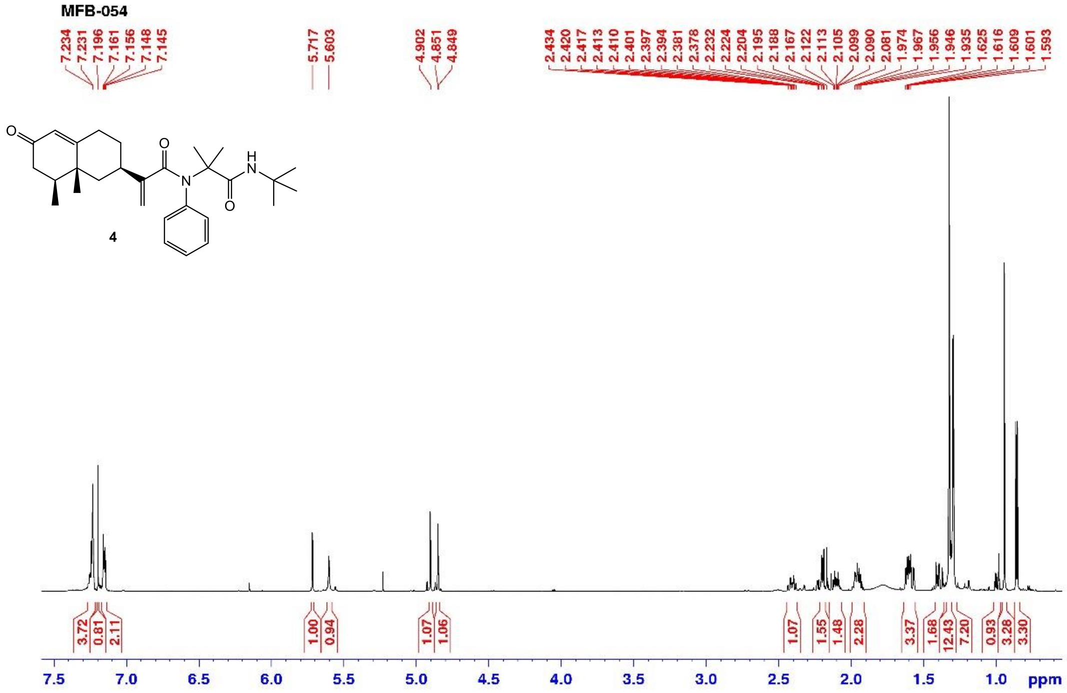

Figure S7. ^{1}H-NMR of **4.**

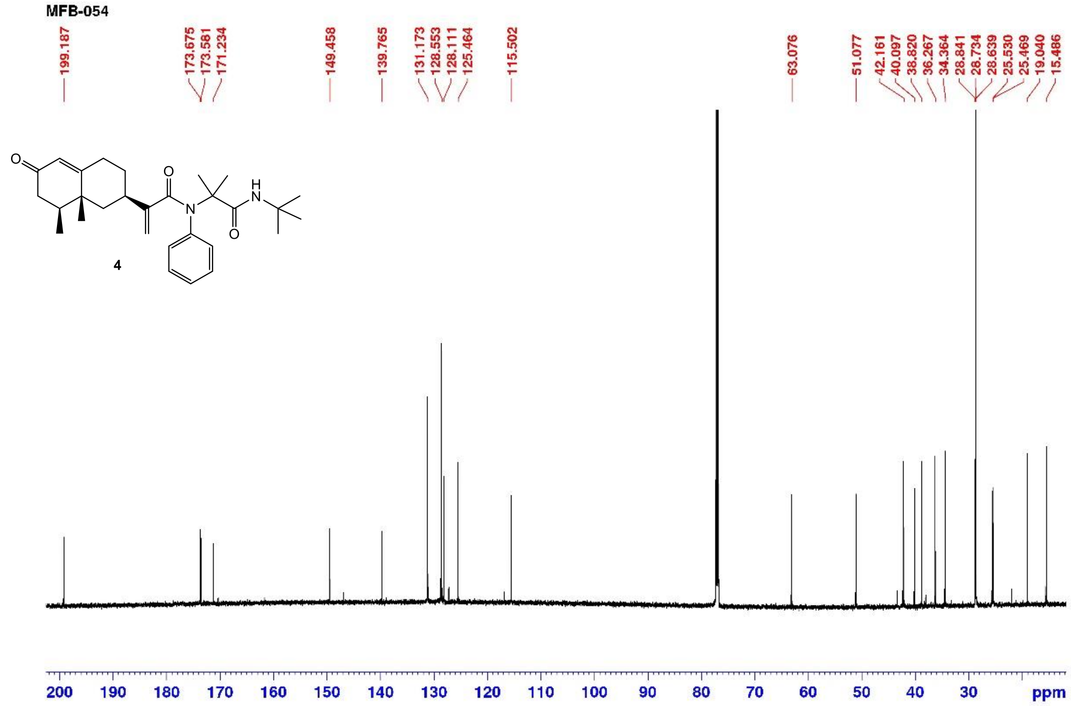

Figure S8. ^{13}C-NMR of **4.**

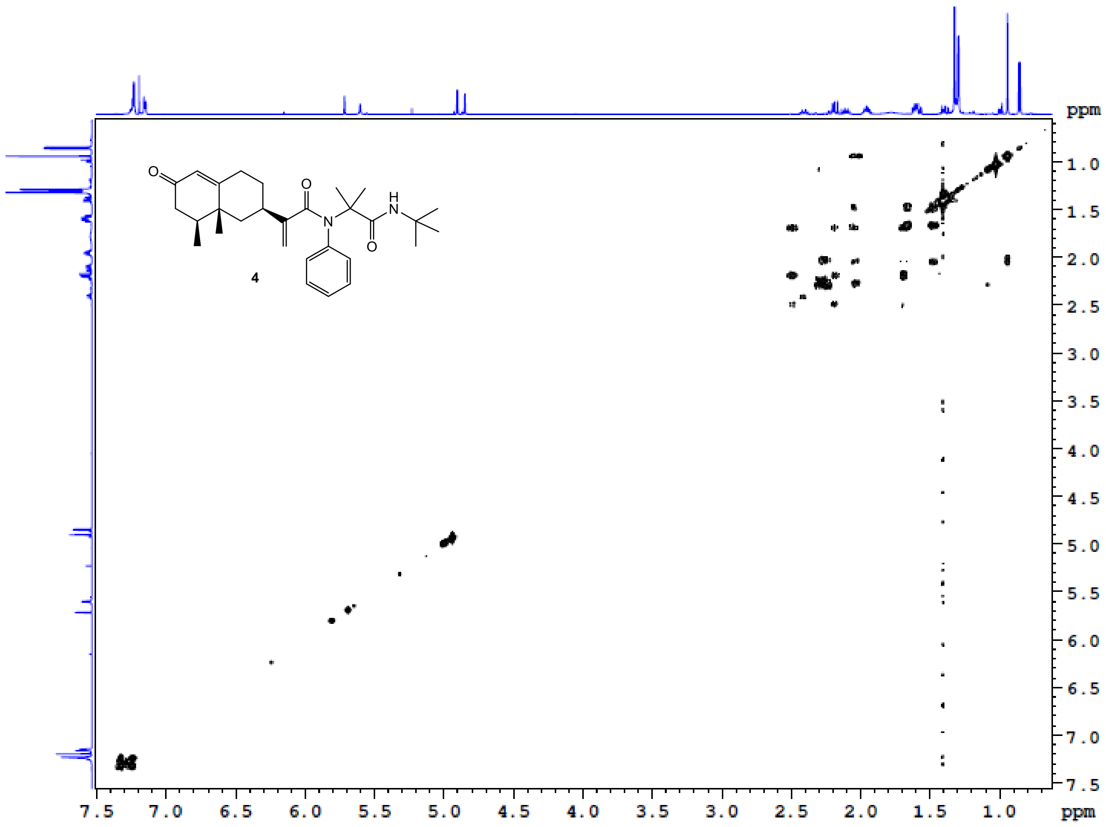

Figure S9. COSY of **4.**

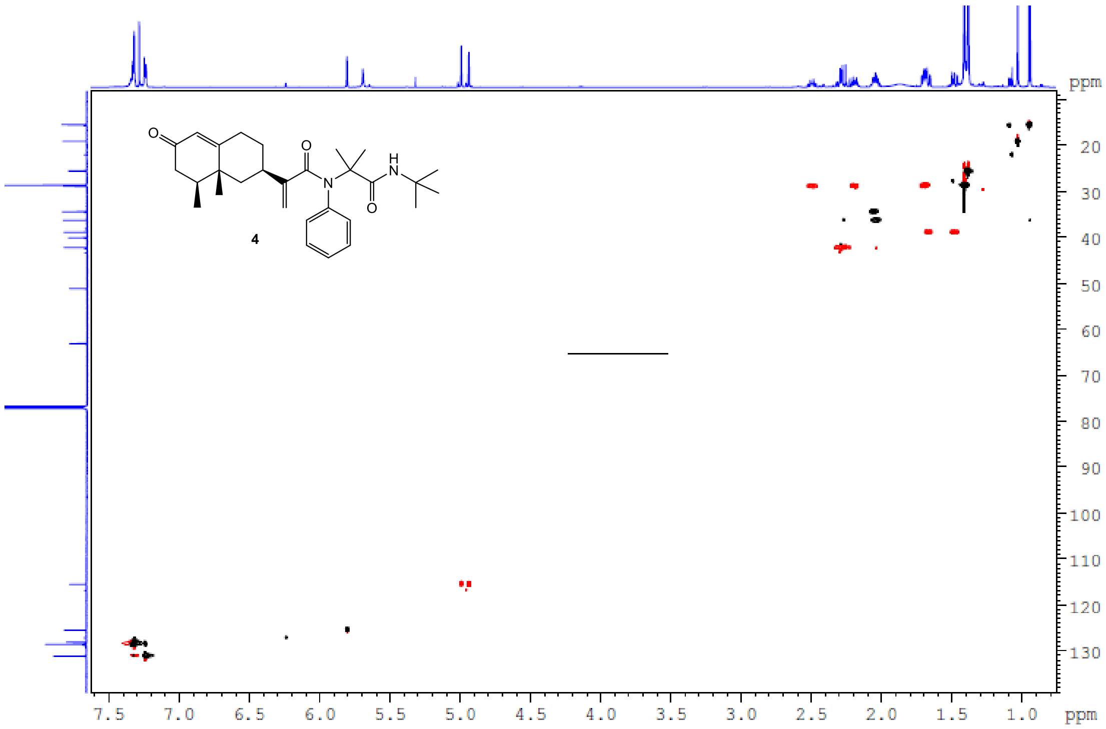

Figure S10. HSQC of **4.**

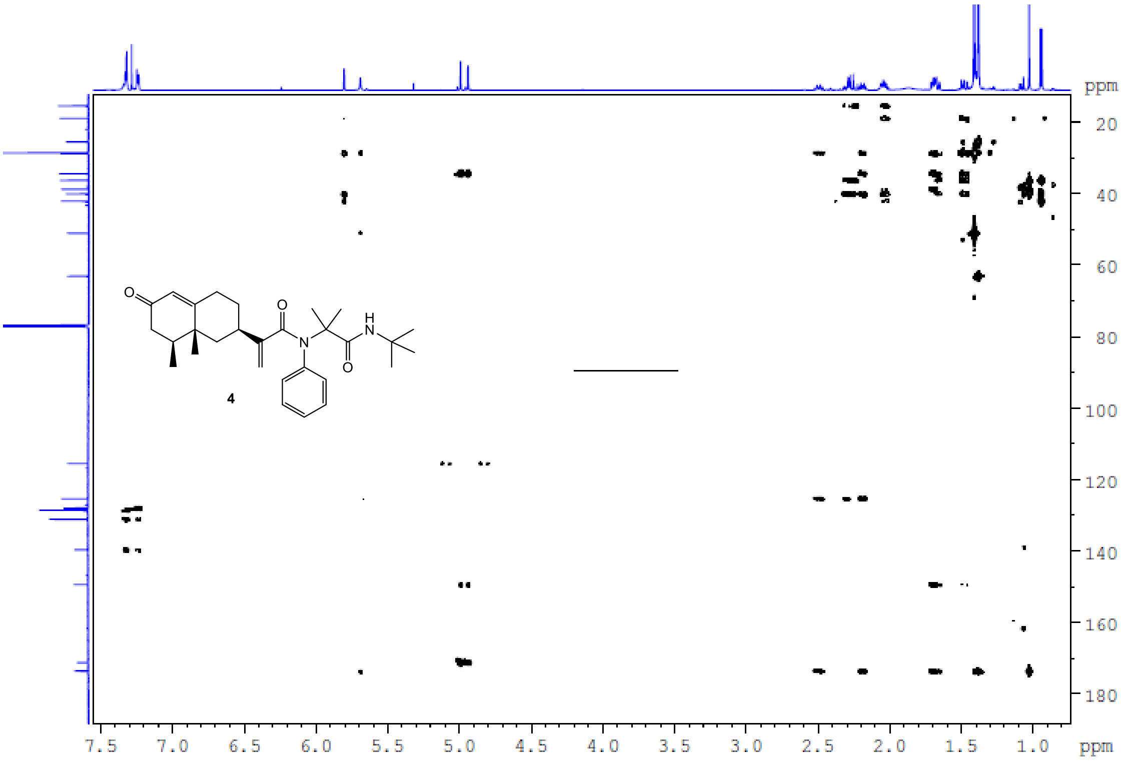

Figure S11. HMBC of **4.**

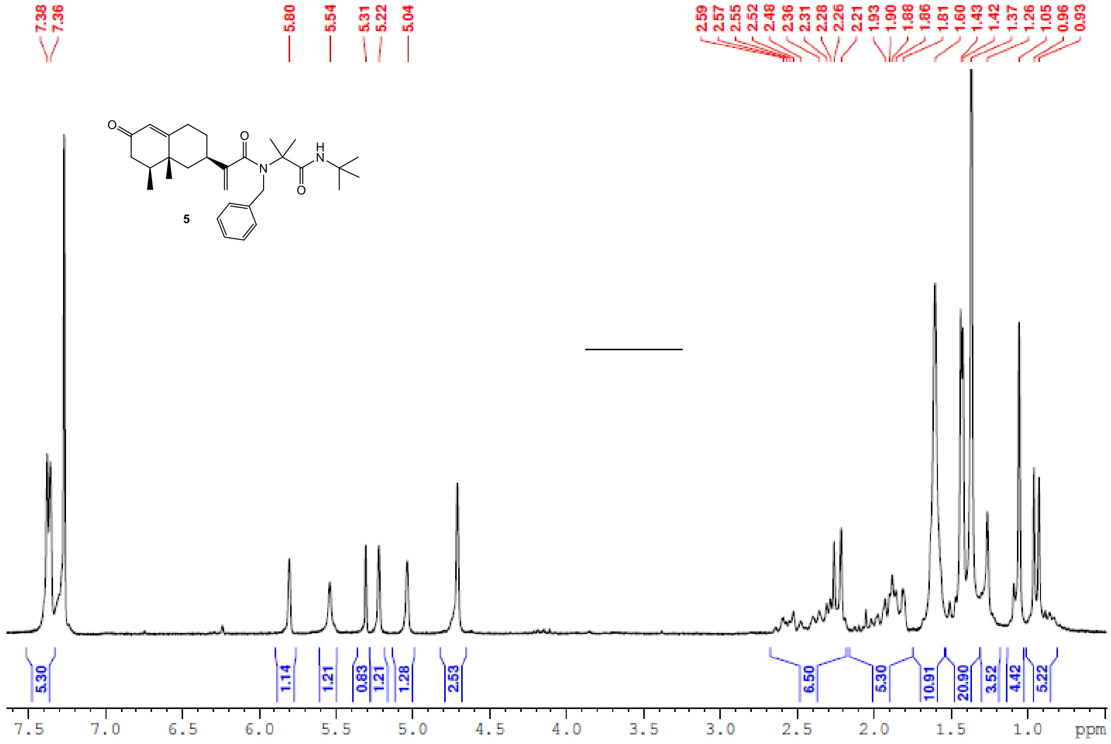

Figure S12. ^{1}H-NMR of **5.**

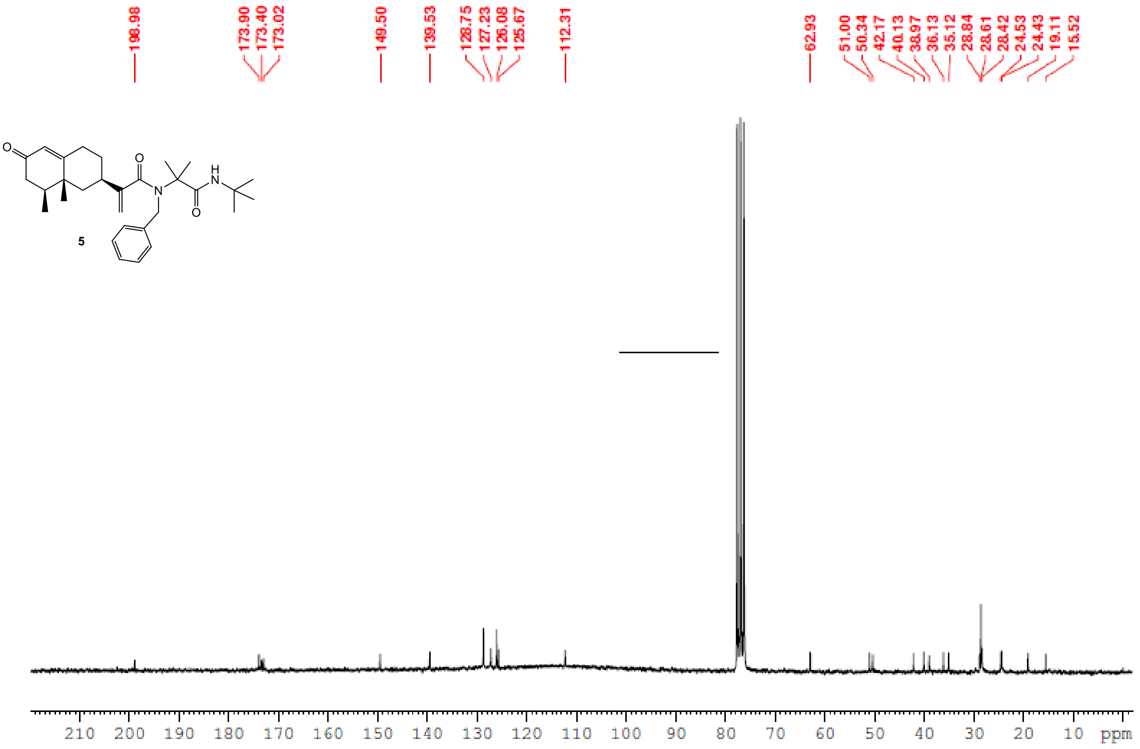

Figure S13. ^{13}C-NMR of **5.**

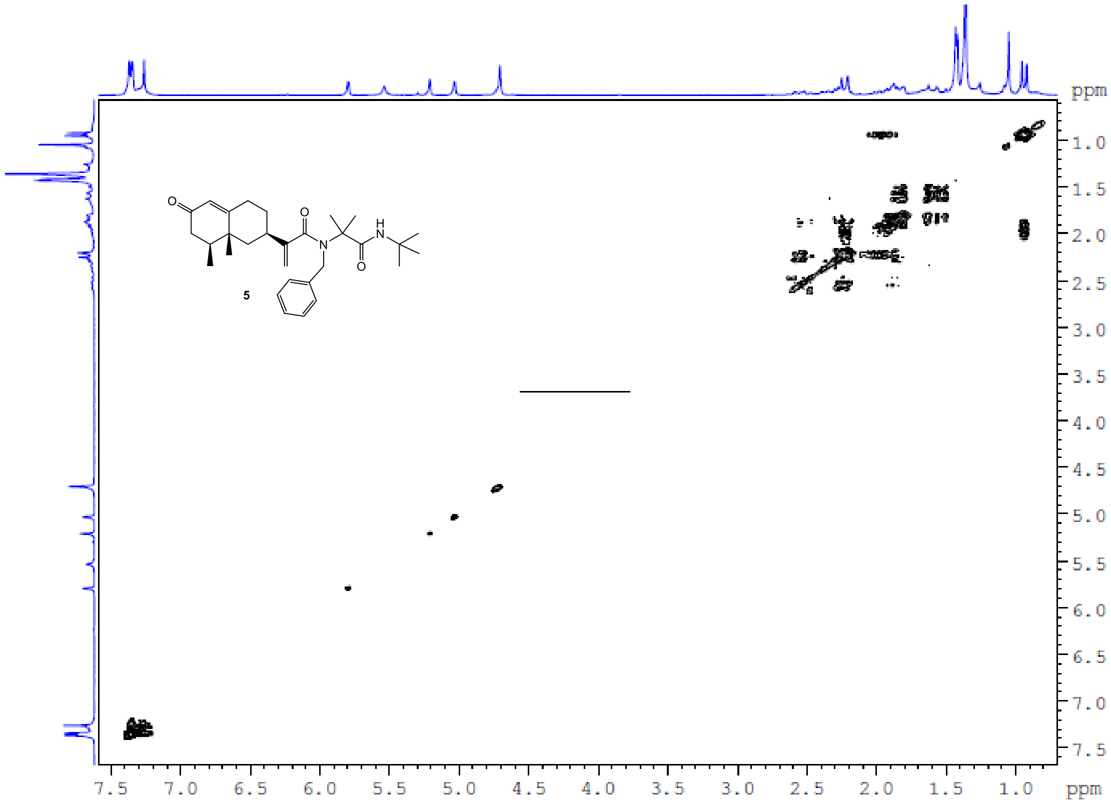

Figure S14. COSY of **5.**

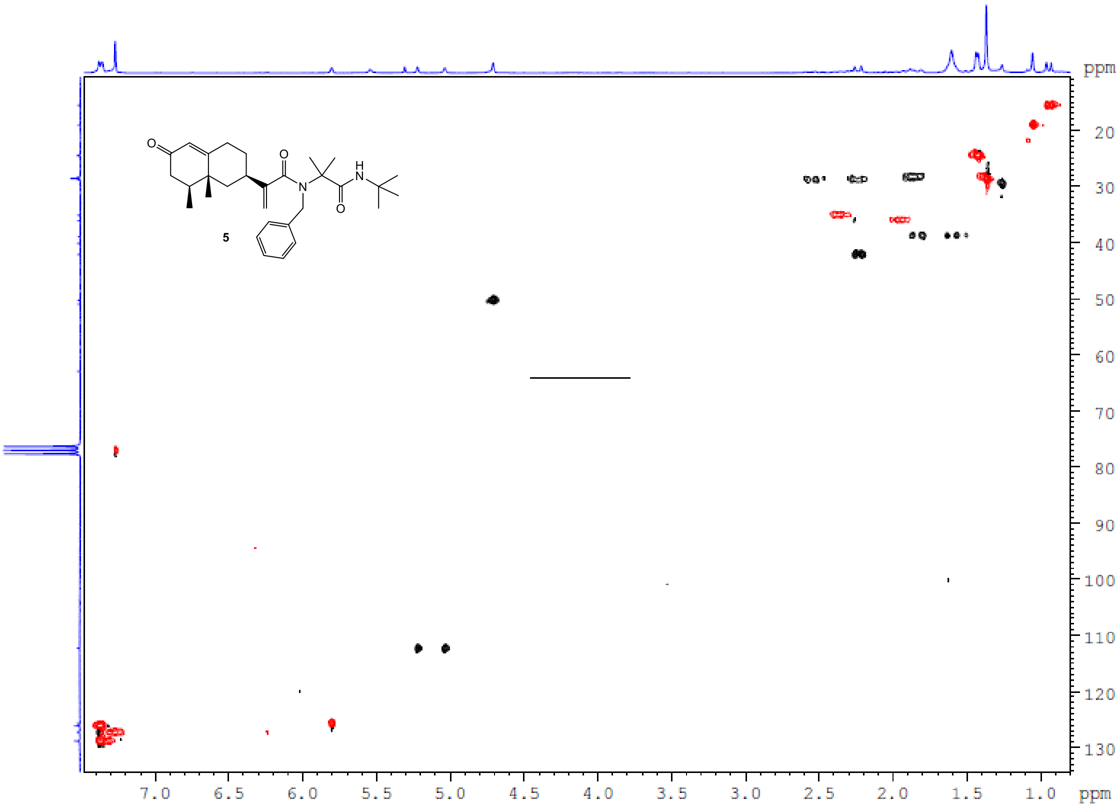

Figure S15. HSQC of **5.**

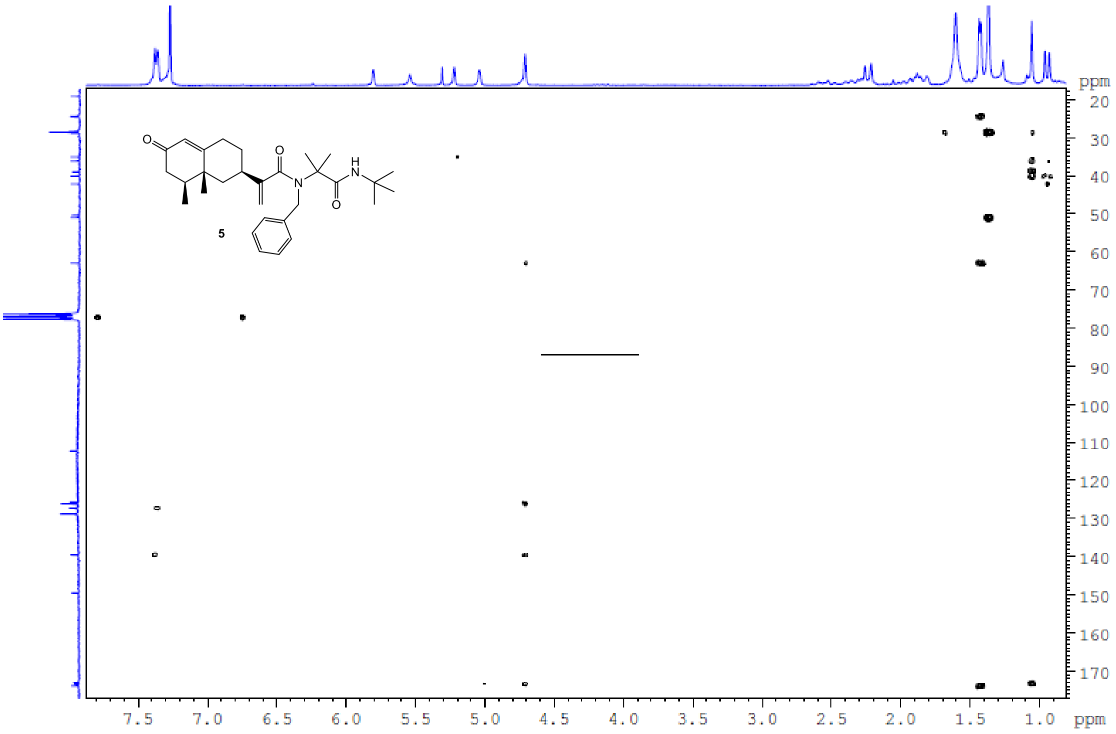

Figure S16. HMBC of **5.**

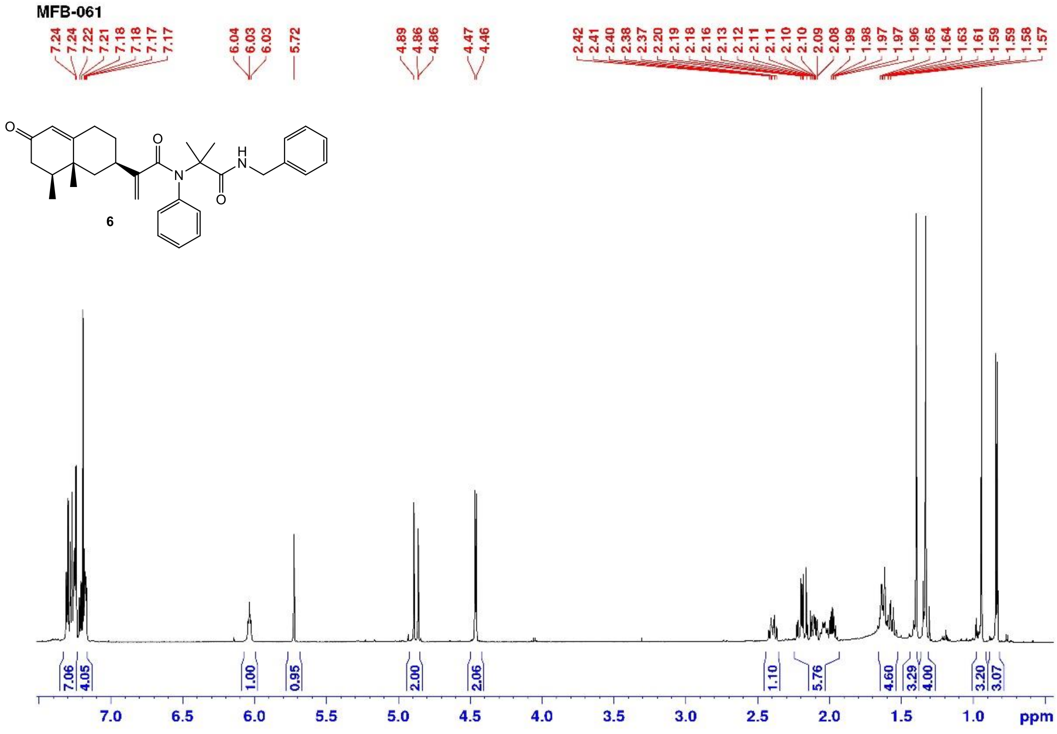

Figure S17. ^{1}H-NMR of **6.**

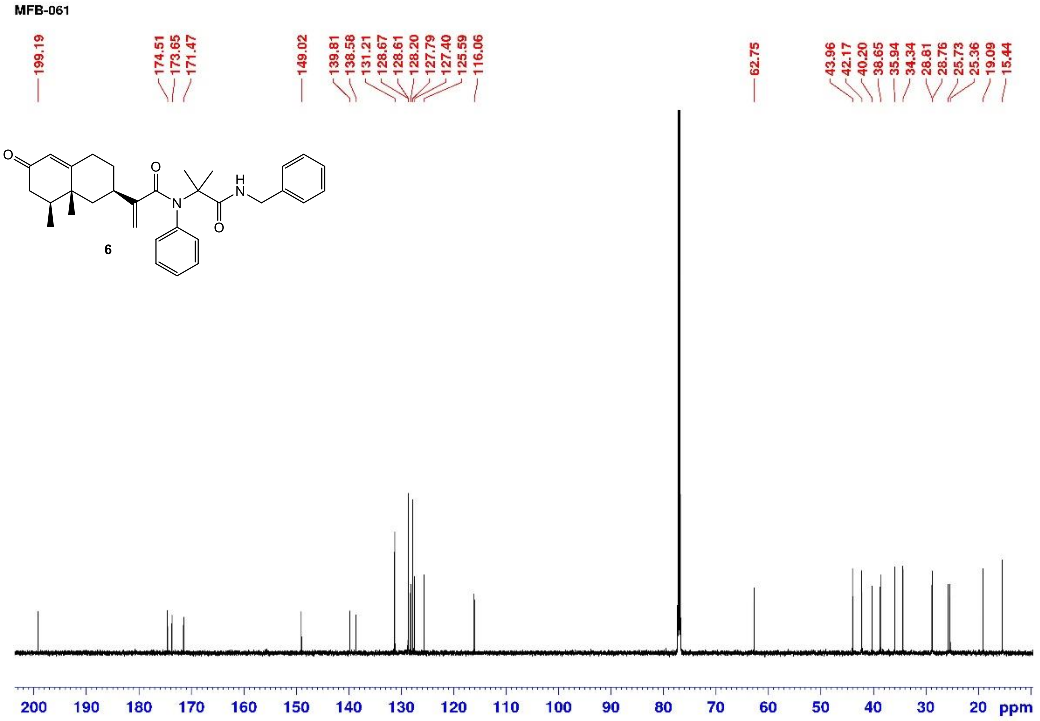

Figure S18. ^{13}C-NMR of **6.**

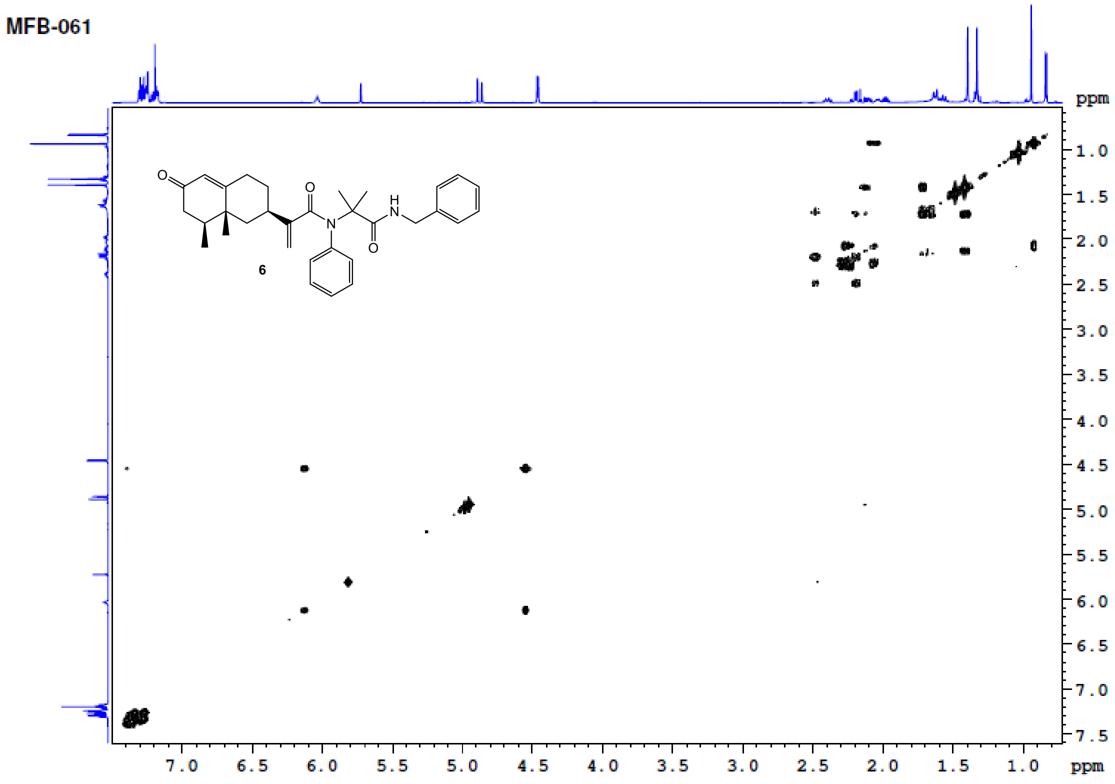

Figure S19. COSY of **6.**

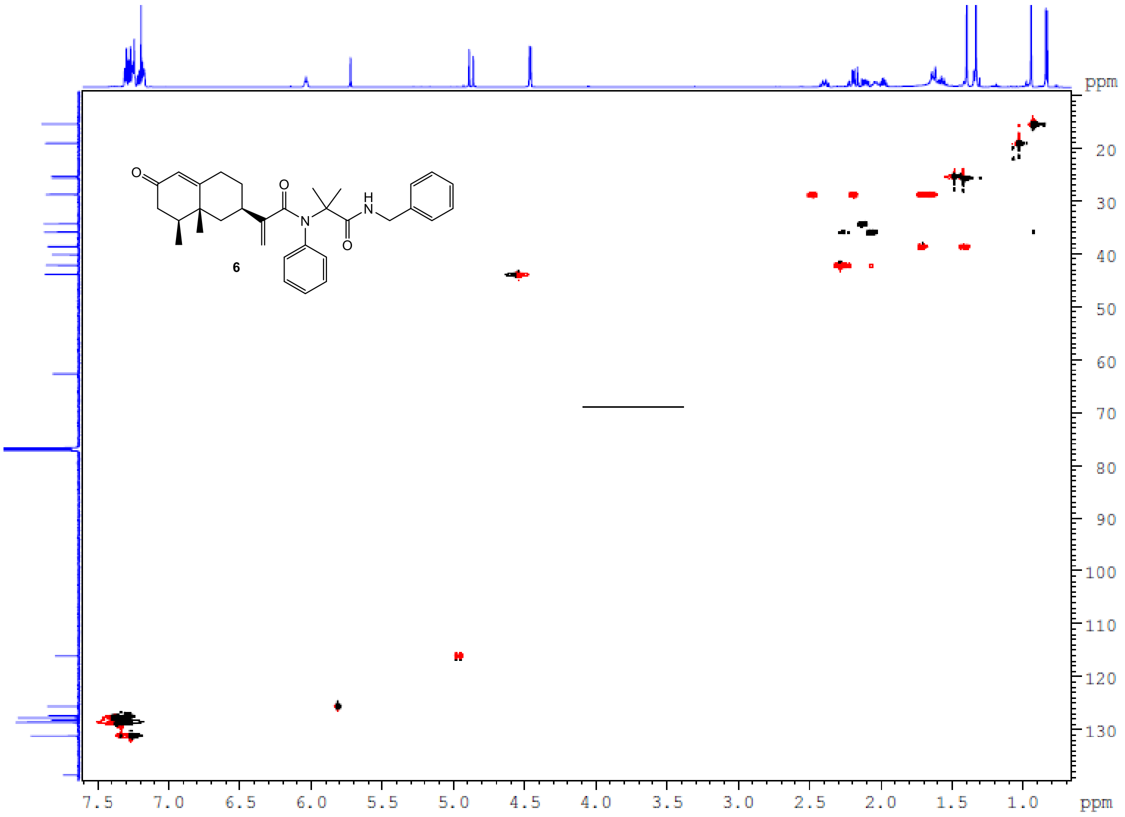

Figure S20. HSQC of **6.**

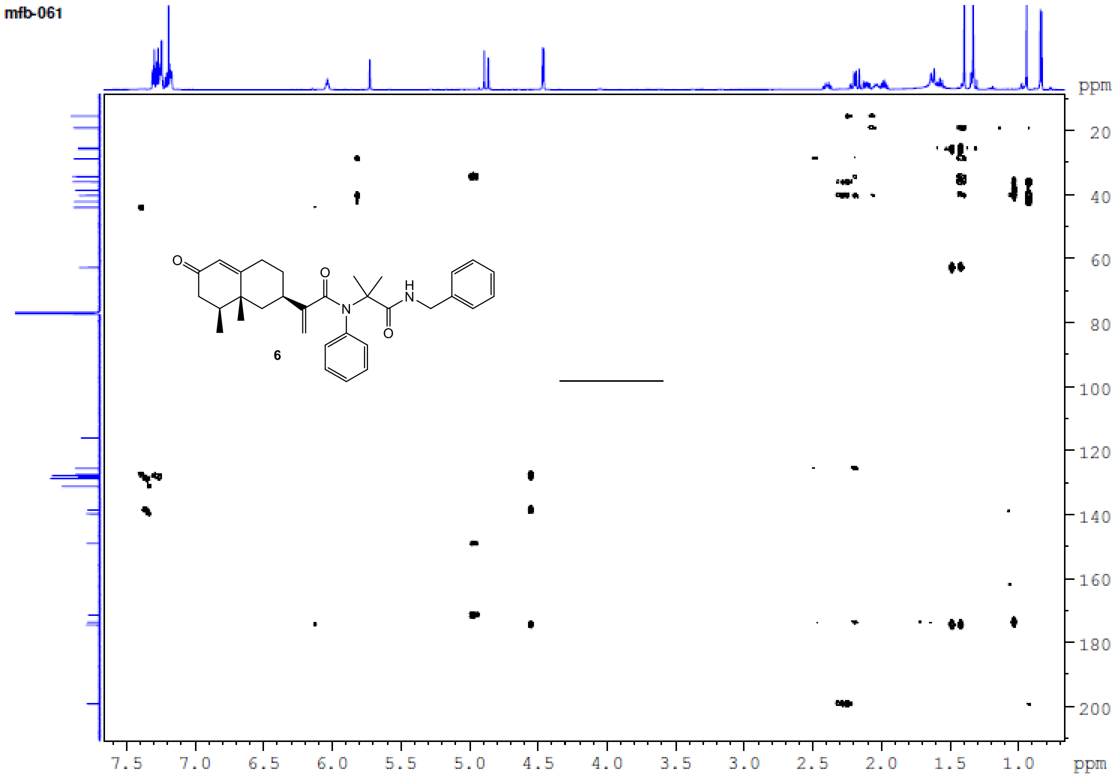

Figure S21. HMBC of **6.**

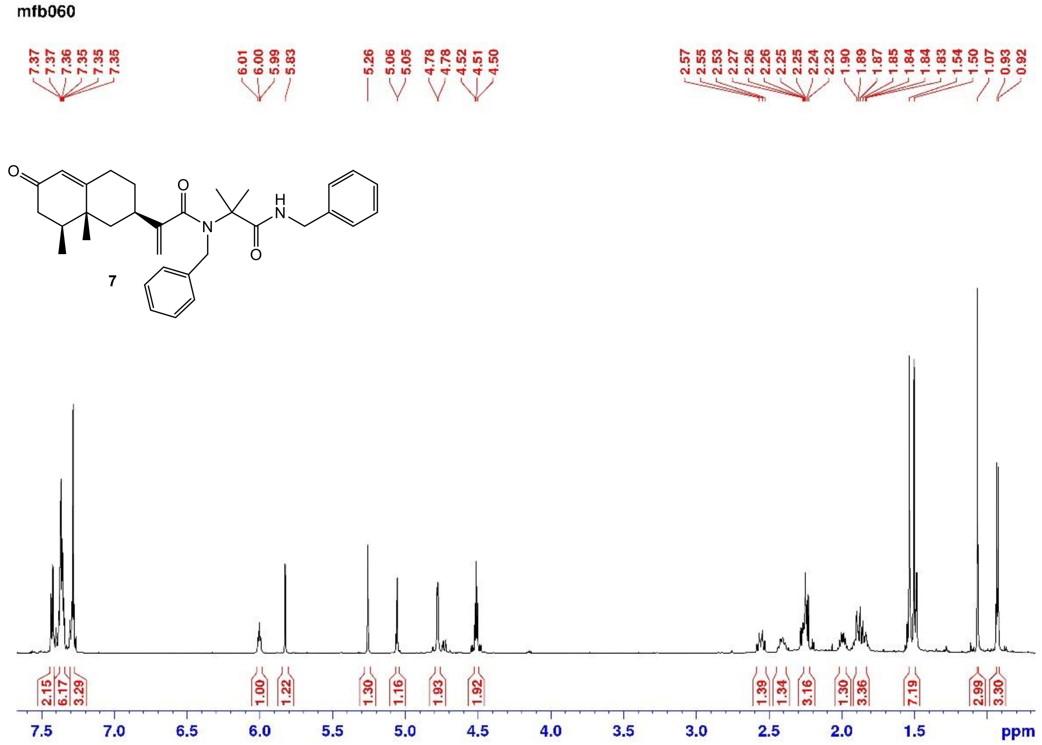

Figure S22. ^{1}H-NMR of **7.**

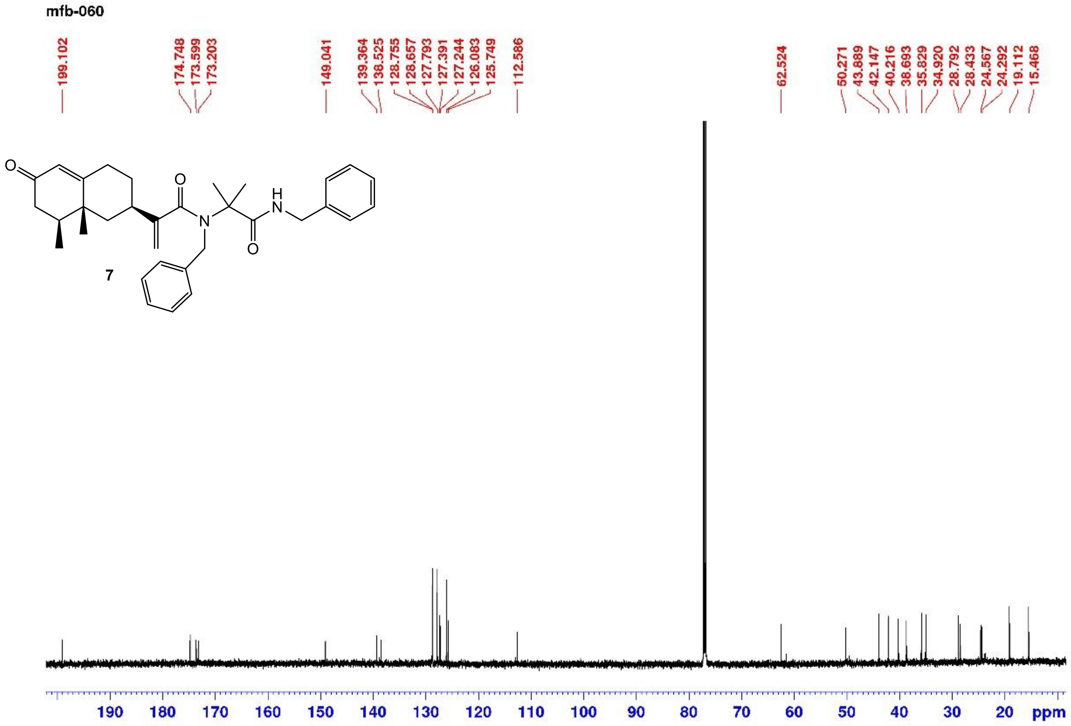

Figure S23. ^{13}C-NMR of **7.**

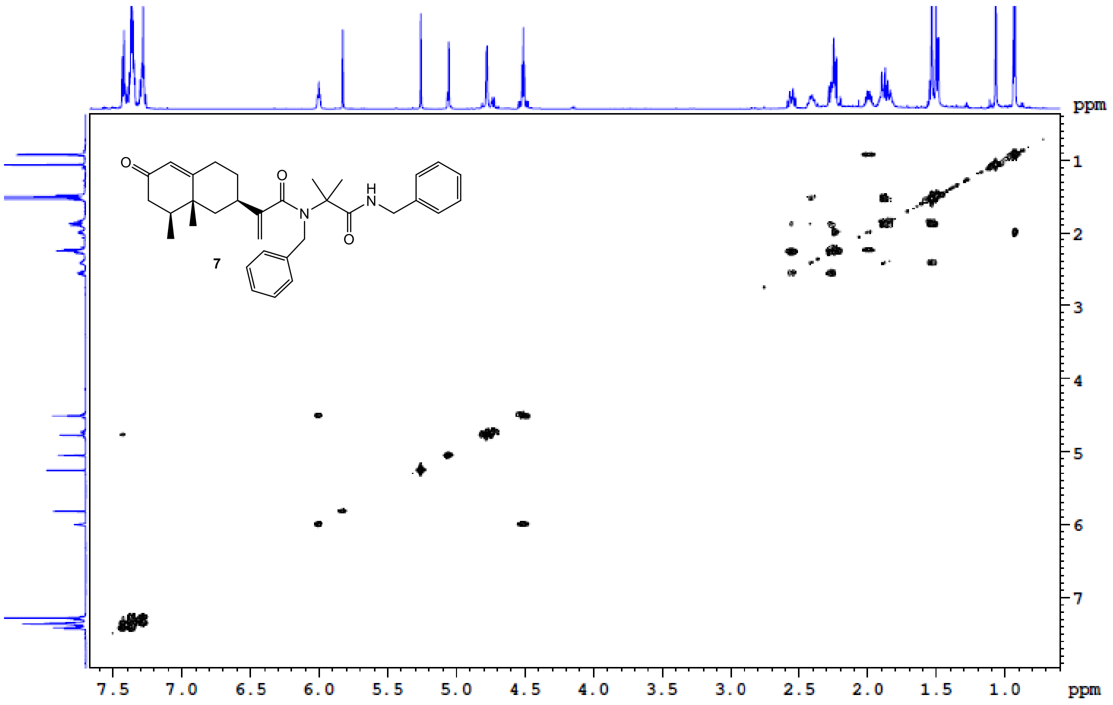

Figure S24. COSY of **7.**

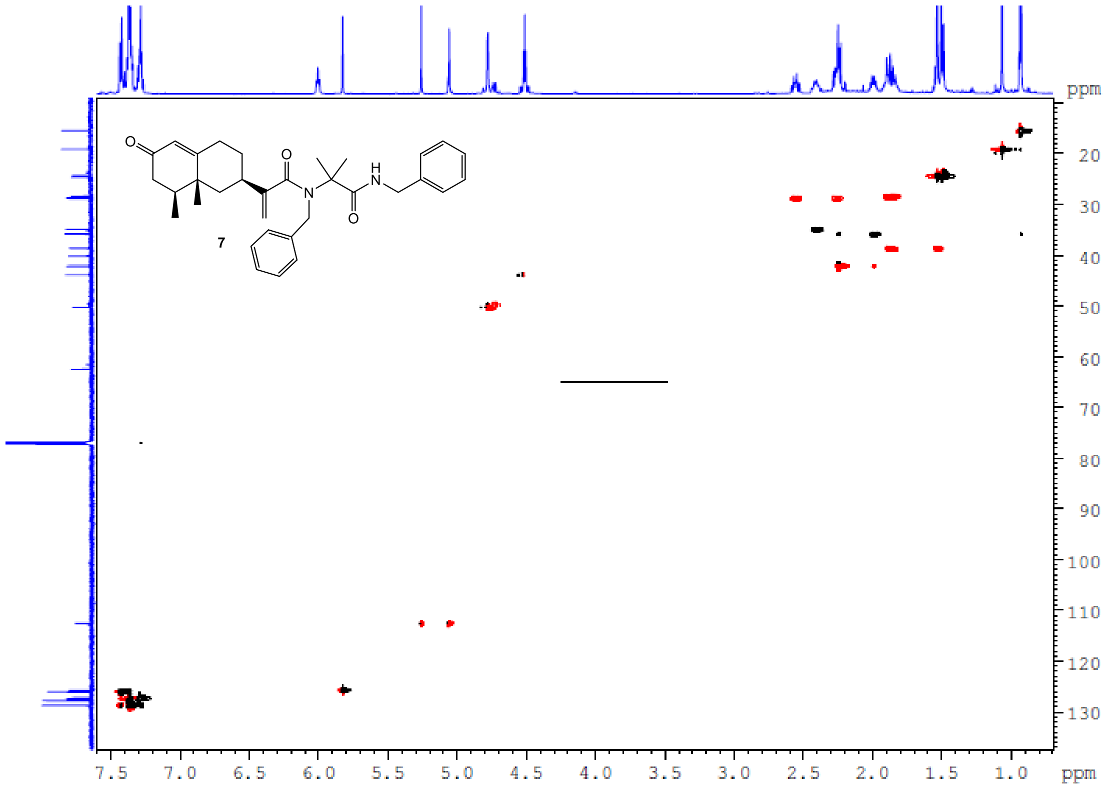

Figure S25. HSQC of **7.**

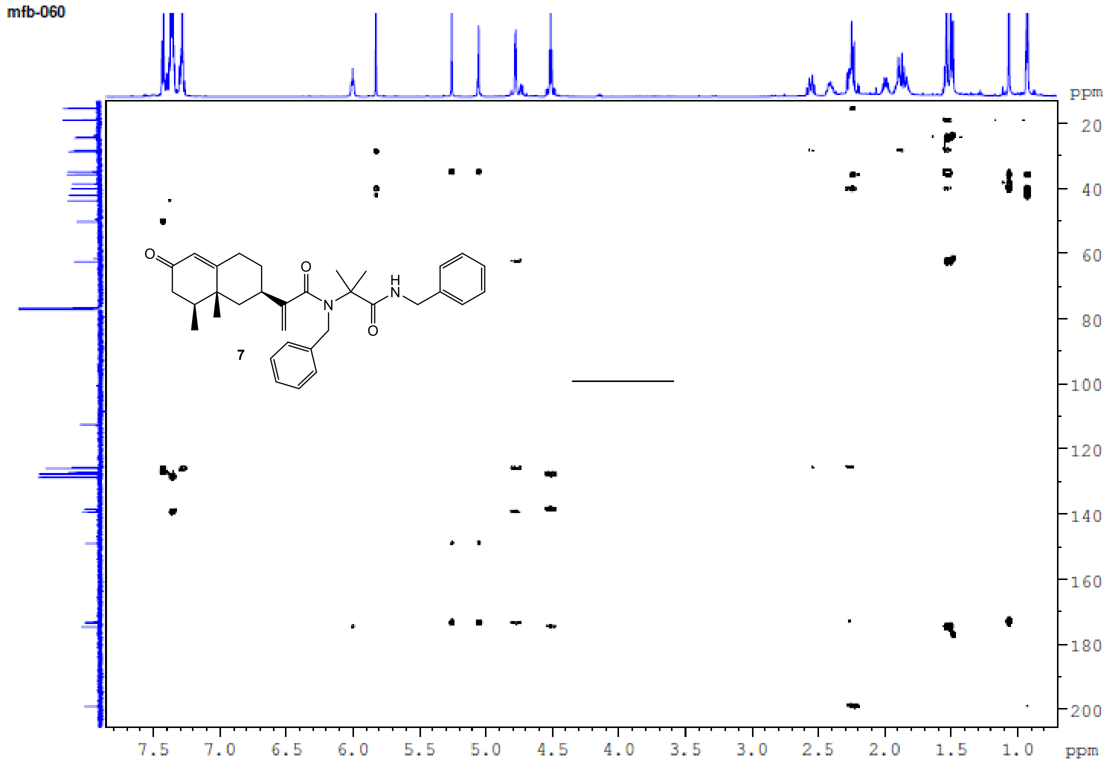

Figure S26. HMBC of **7.**

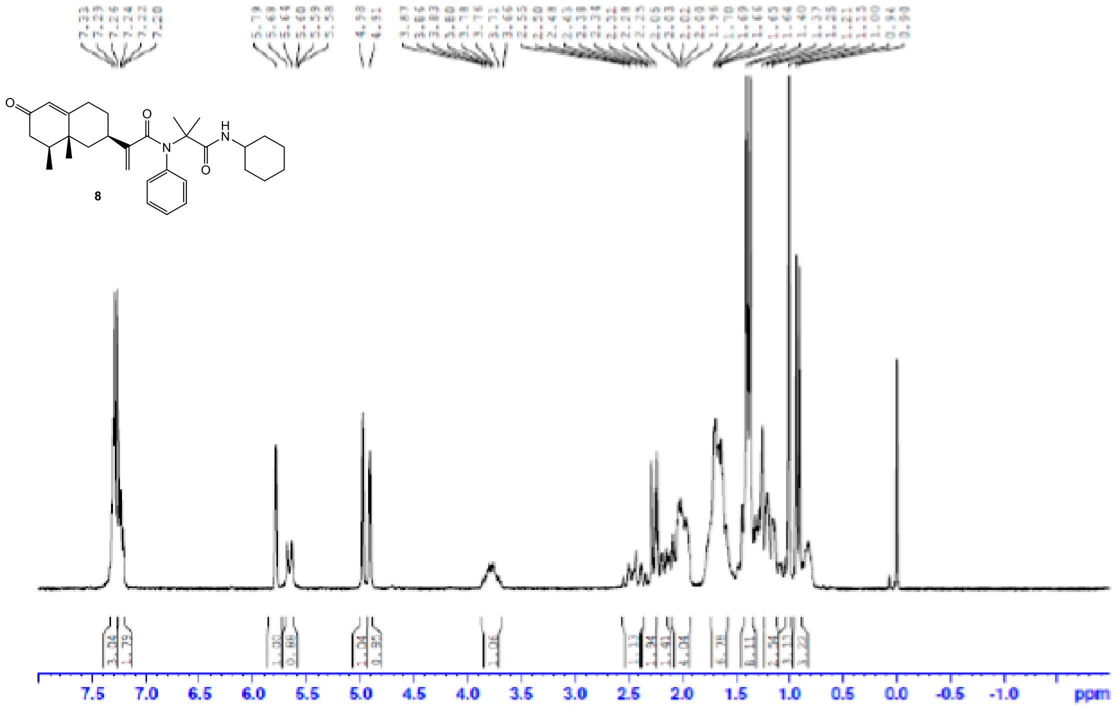

Figure S27. ^{1}H-NMR of **8.**

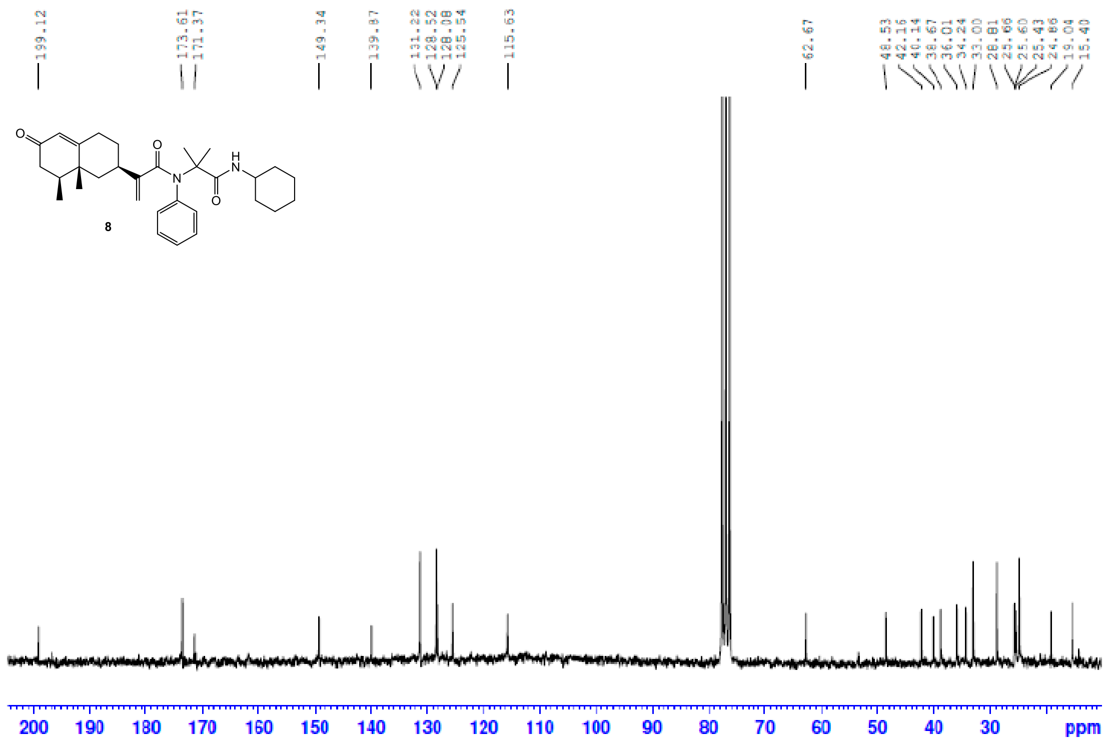

Figure S28. ^{13}C-NMR of **8.**

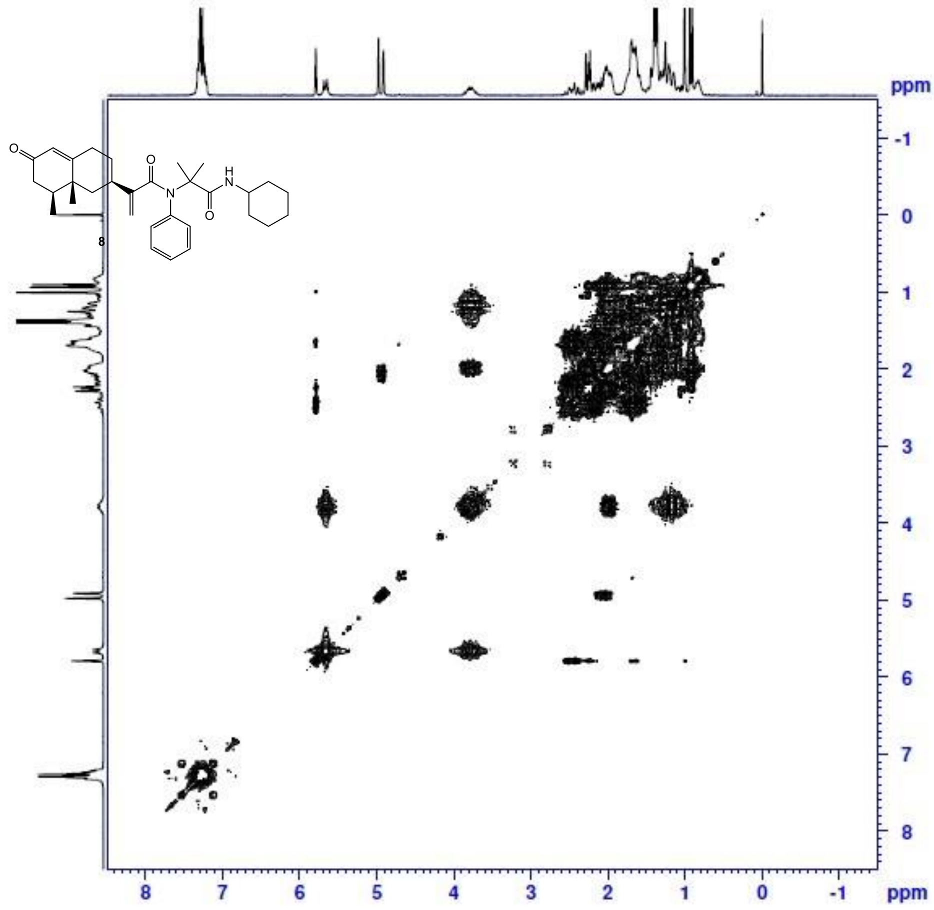

Figure S29. COSY of **8.**

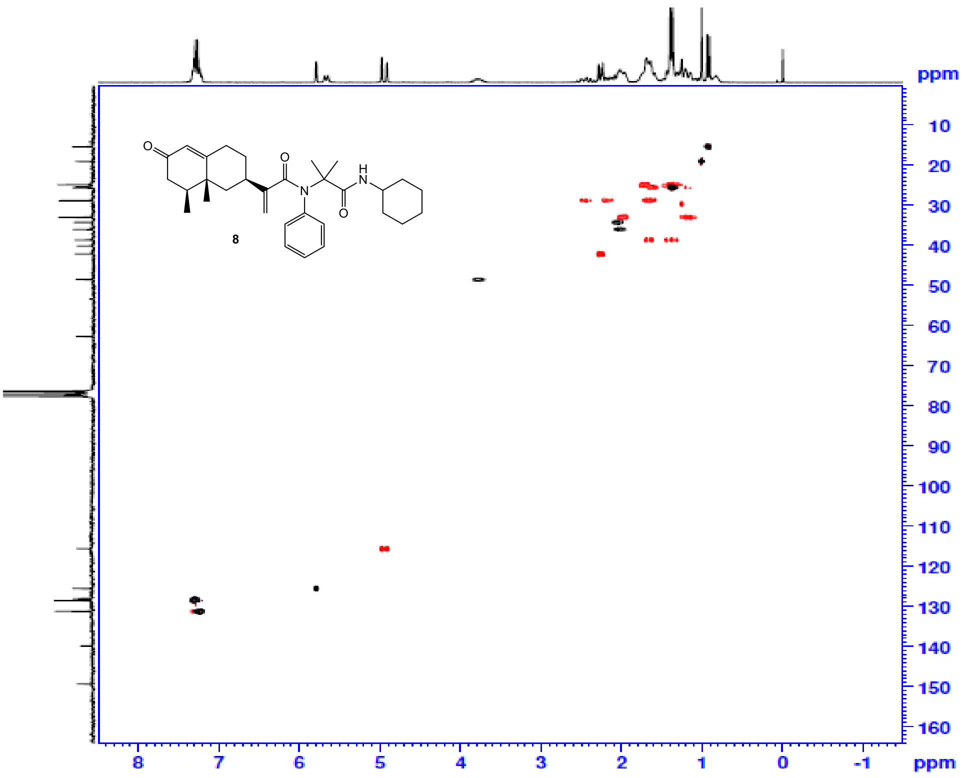

Figure S30. HSQC of **8.**

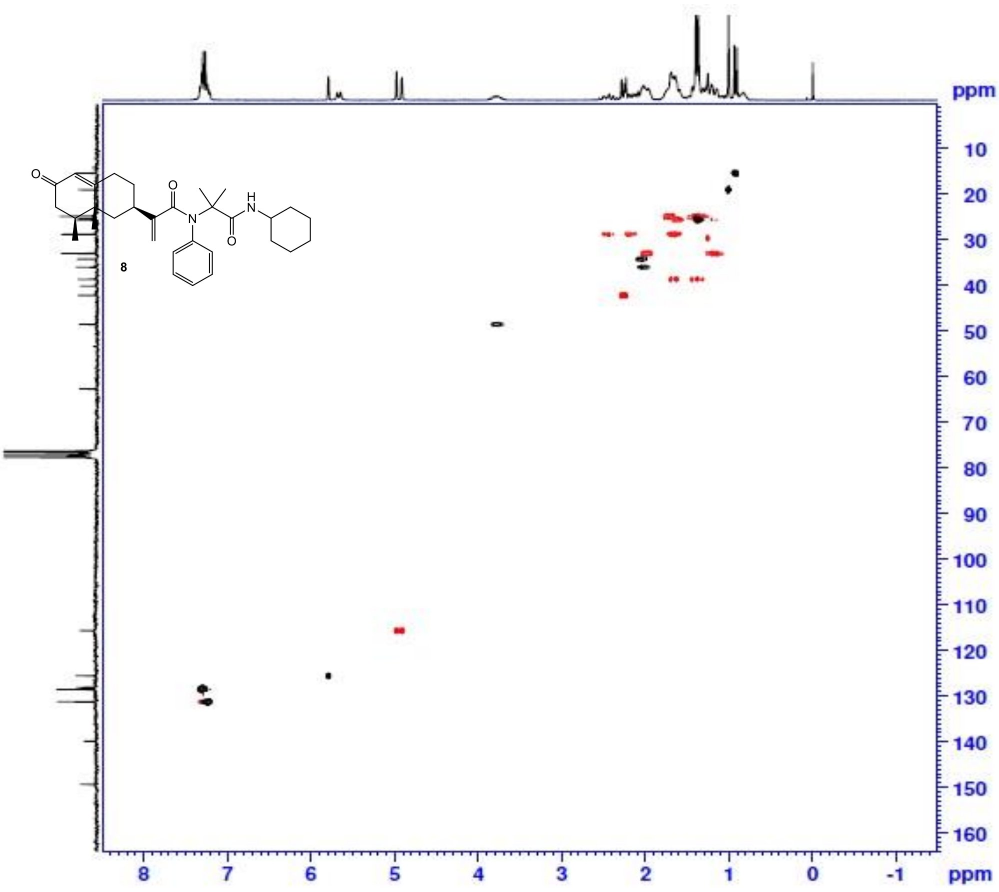

Figure S31. HSQC of **8.**

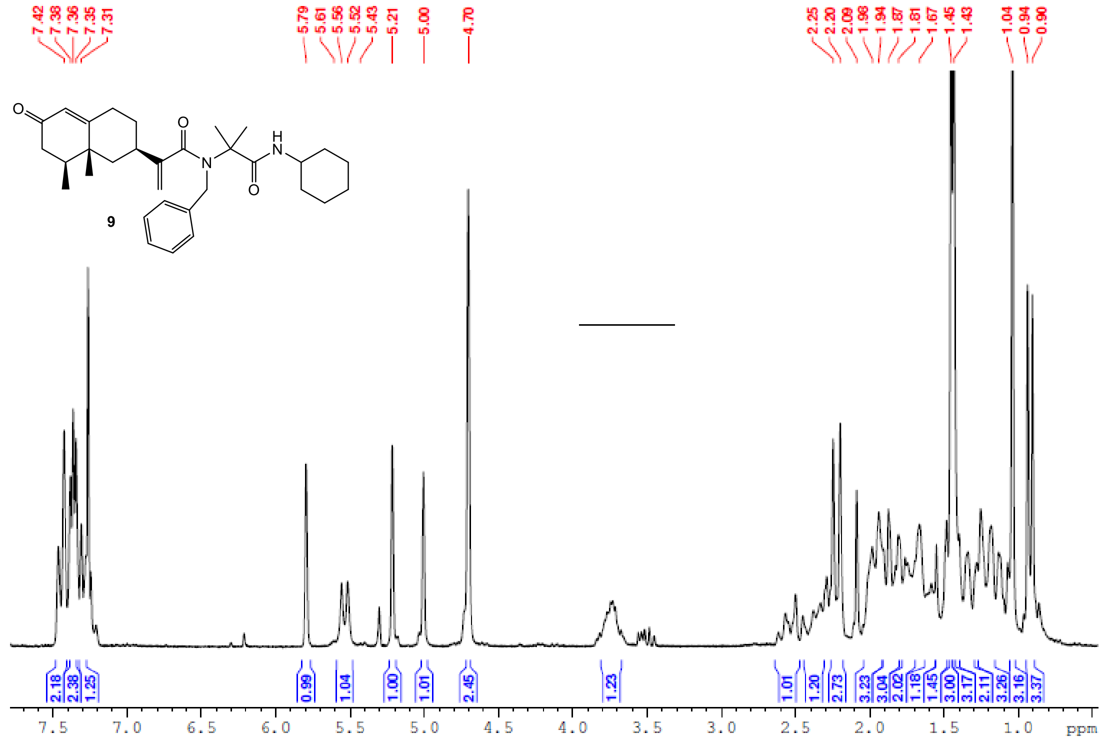

Figure S32. ^{1}H-NMR of **9.**

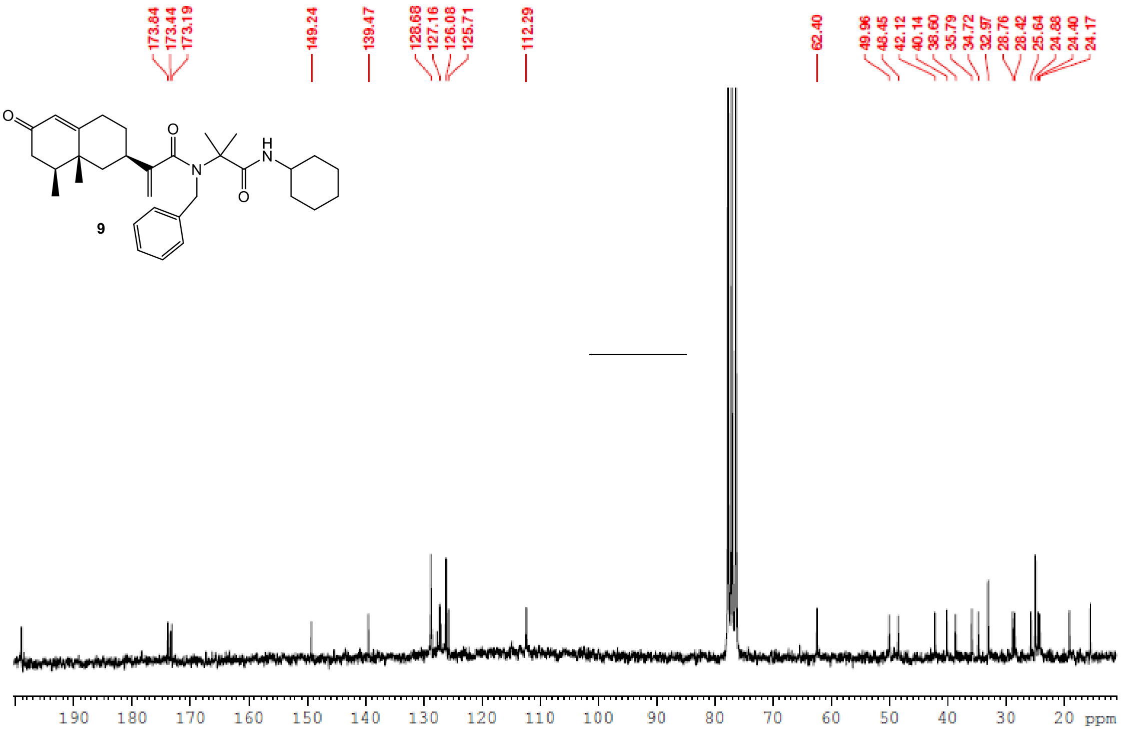

Figure S33. ^{13}C-NMR of **9.**

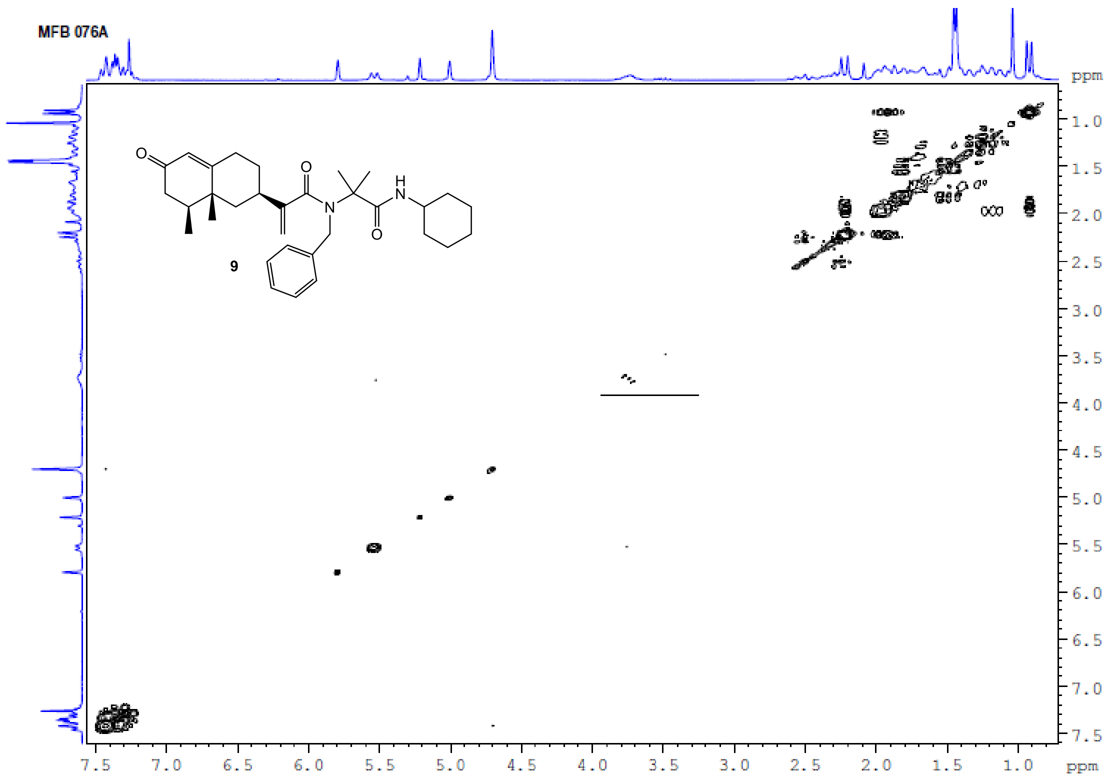

Figure S34. COSY of **9.**

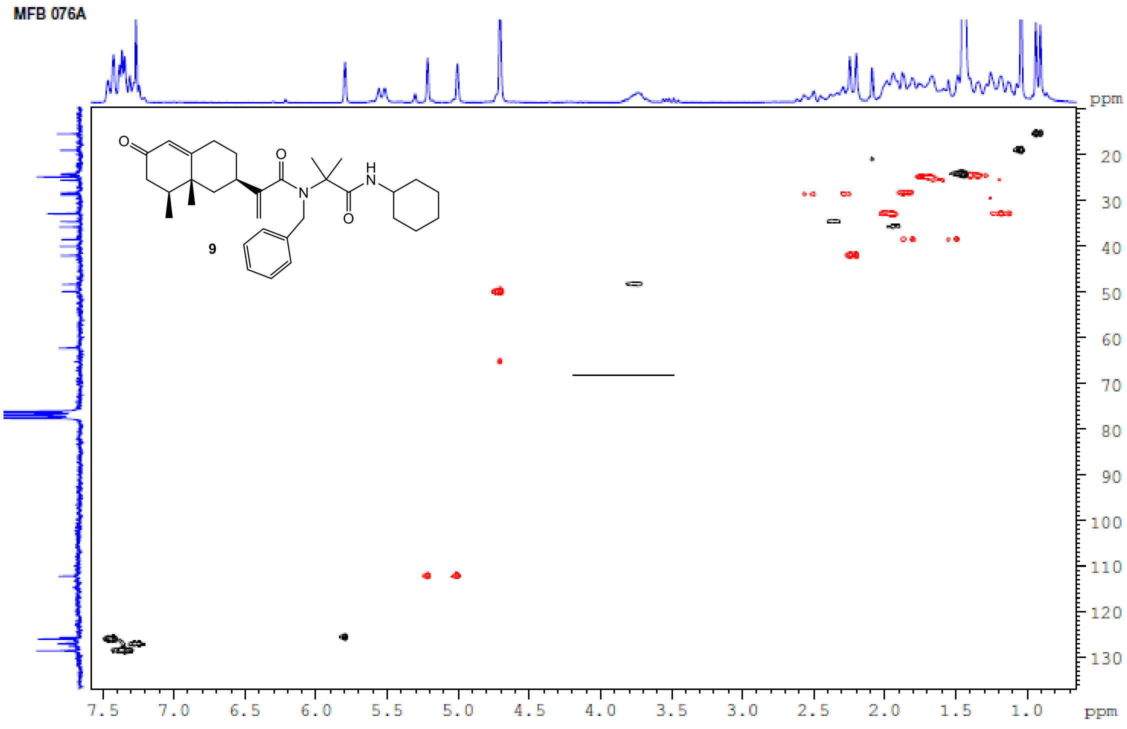

Figure S35. HSQC of **9.**

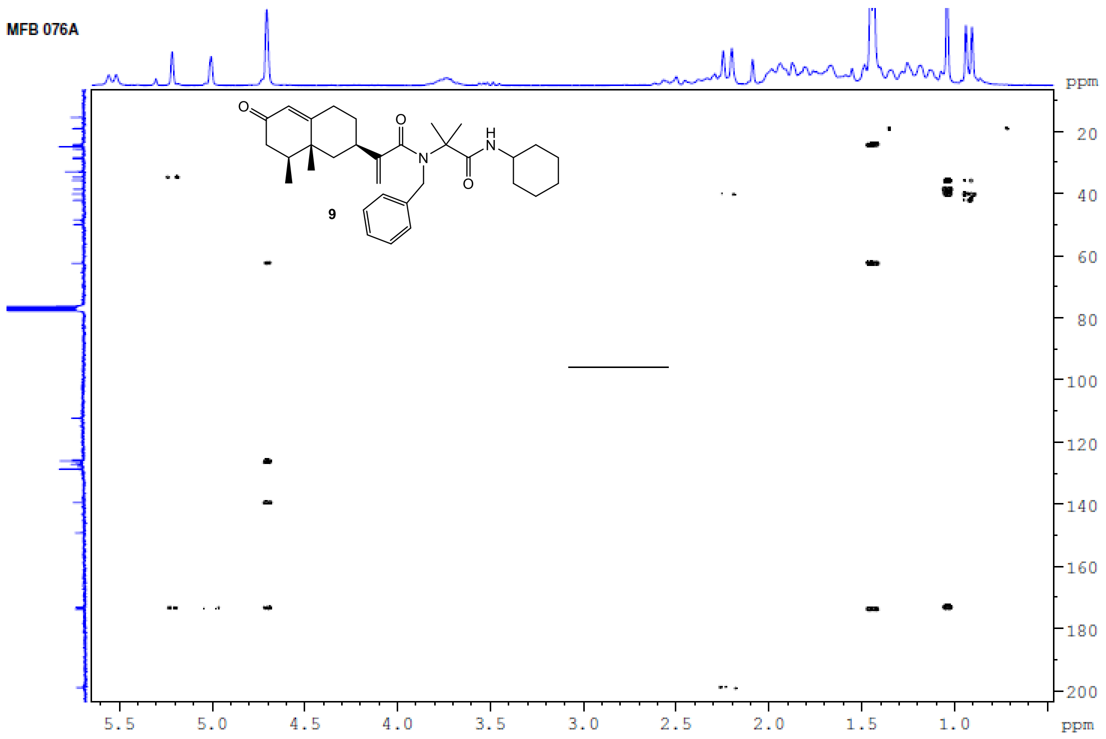

Figure S36. HMBC of **9.**

3. NMR spectra of Ilicic acid derivatives

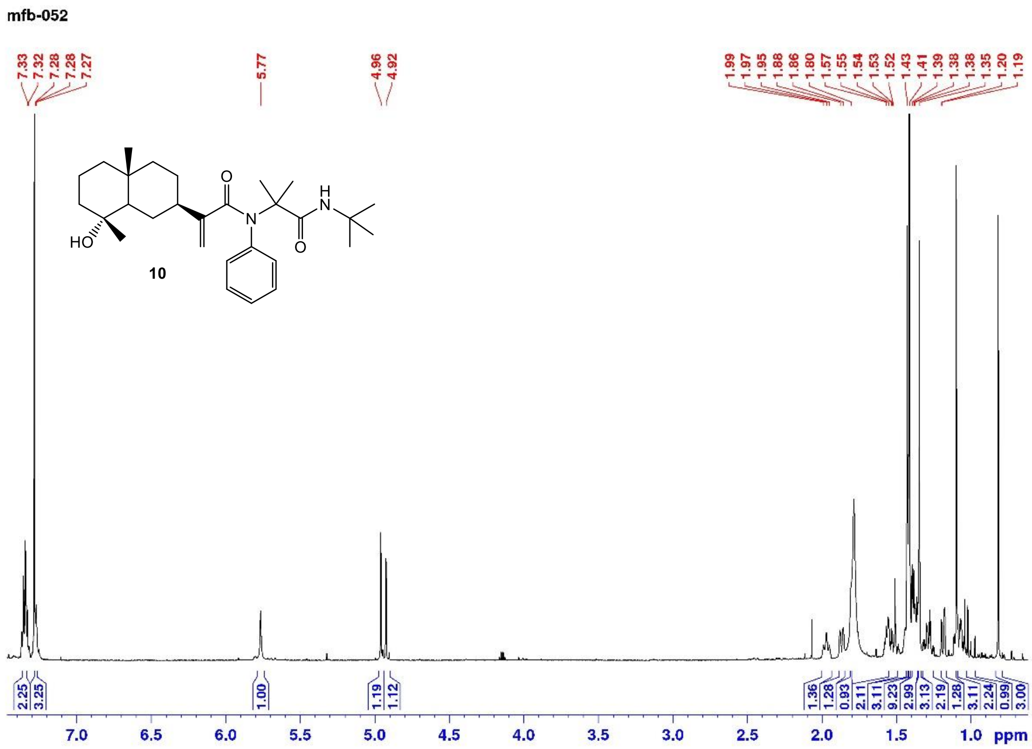

Figure S37. ^{1}H-NMR of **10.**

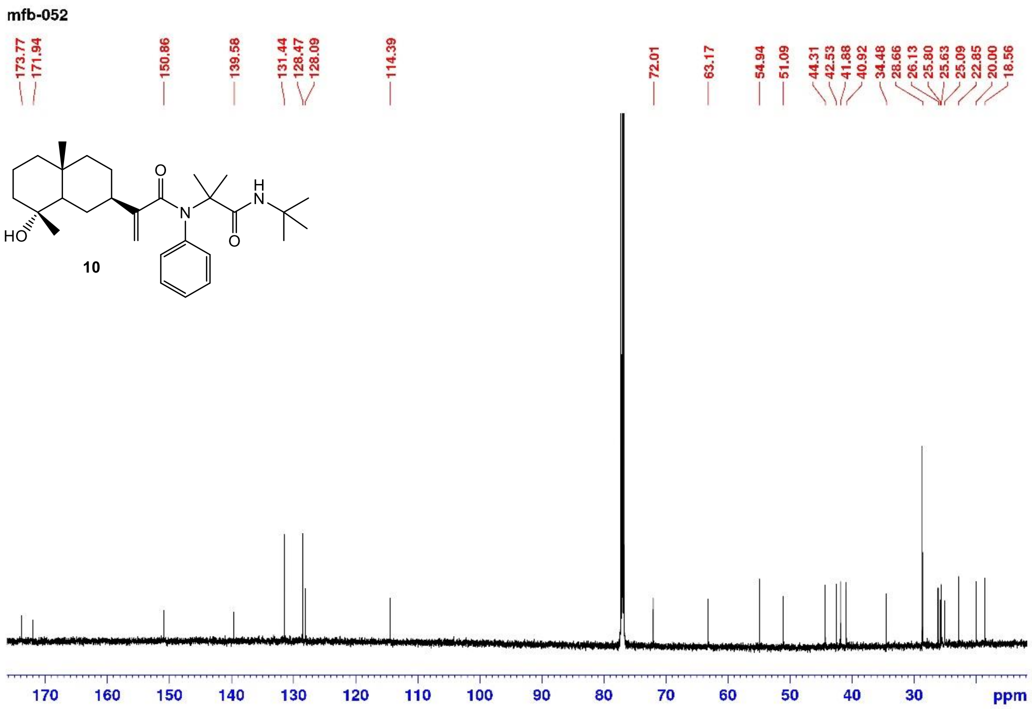

Figure S38. ^{13}C-NMR of **10.**

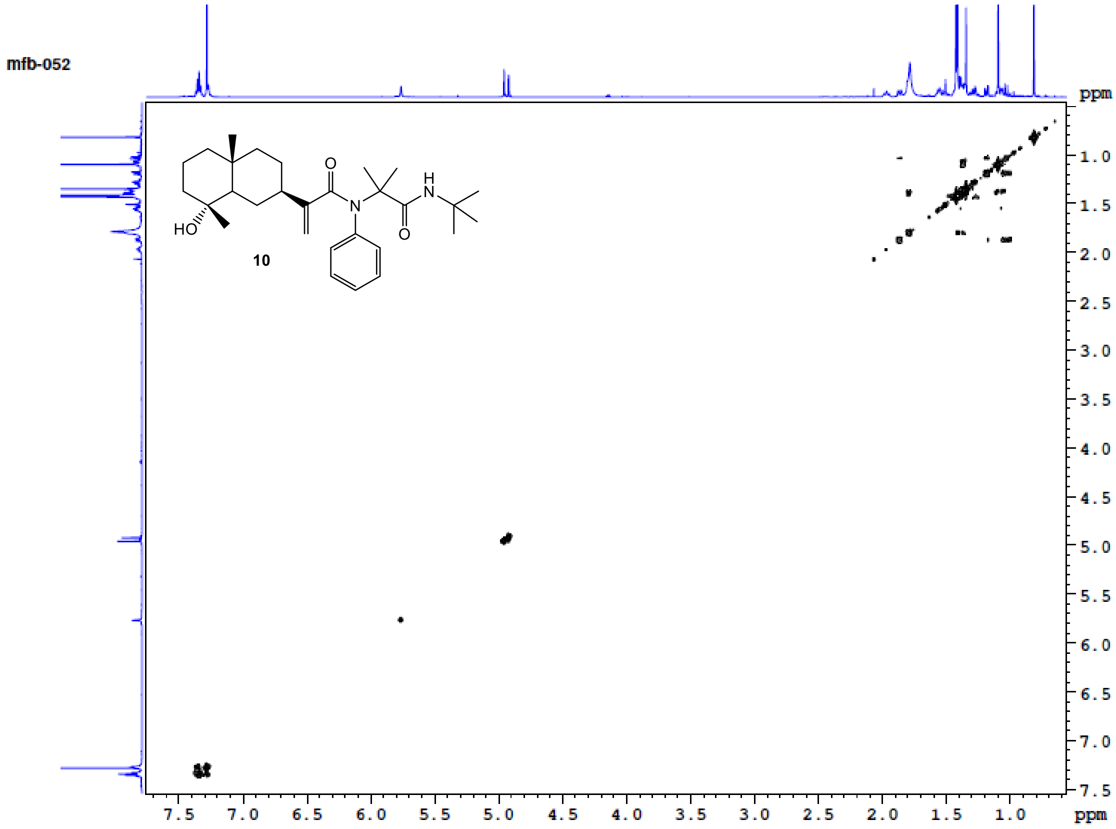

Figure S39. COSY of **10.**

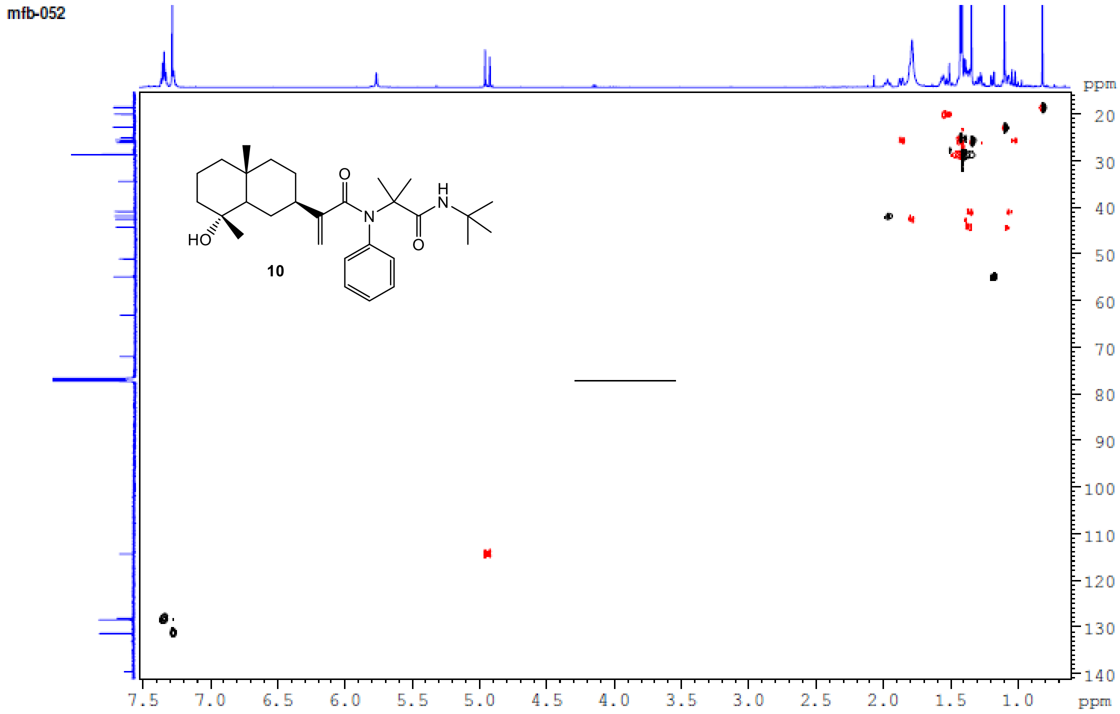

Figure S40. HSQC of **10.**

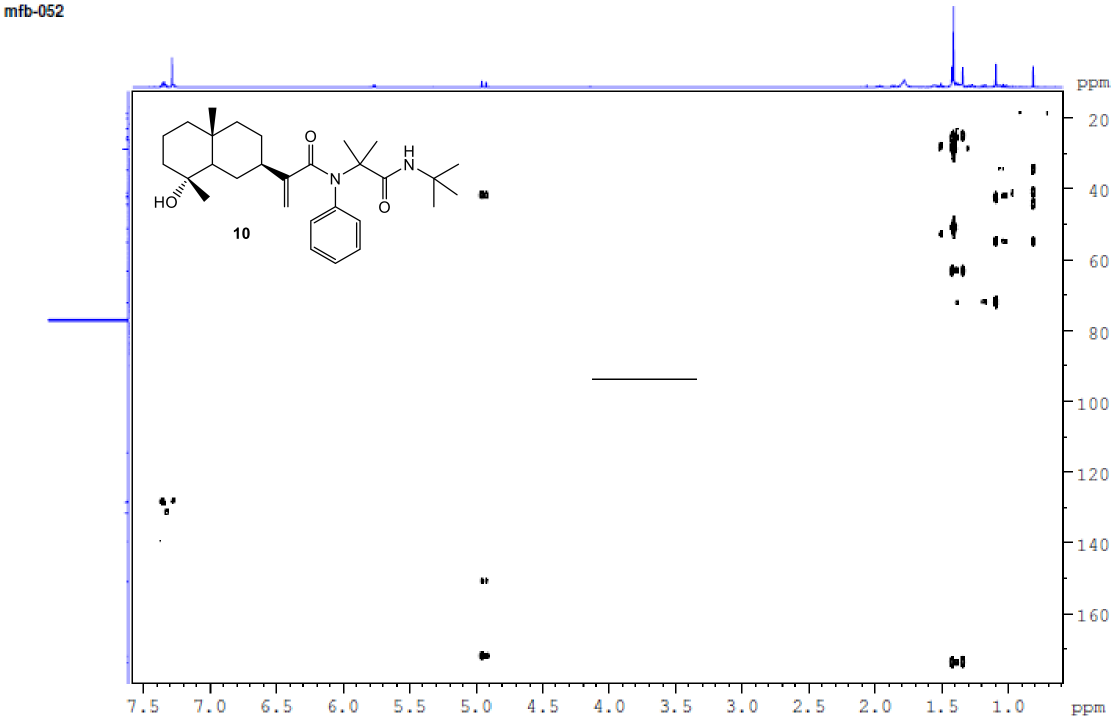

Figure S41. HMBC of **10.**

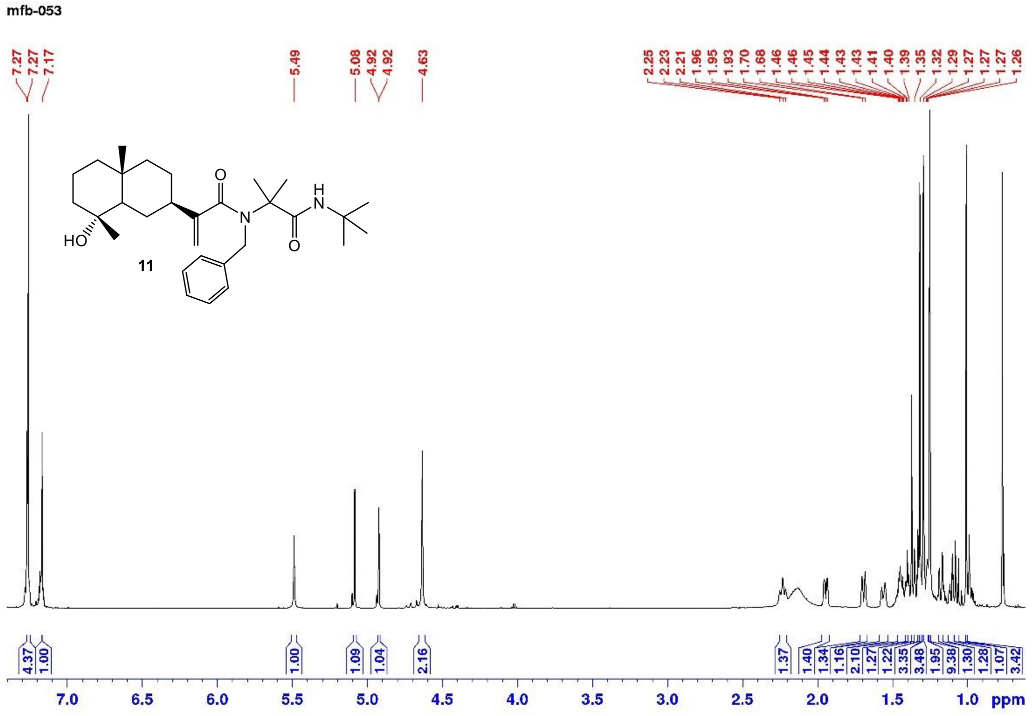

Figure S42. ^{1}H-NMR of **11.**

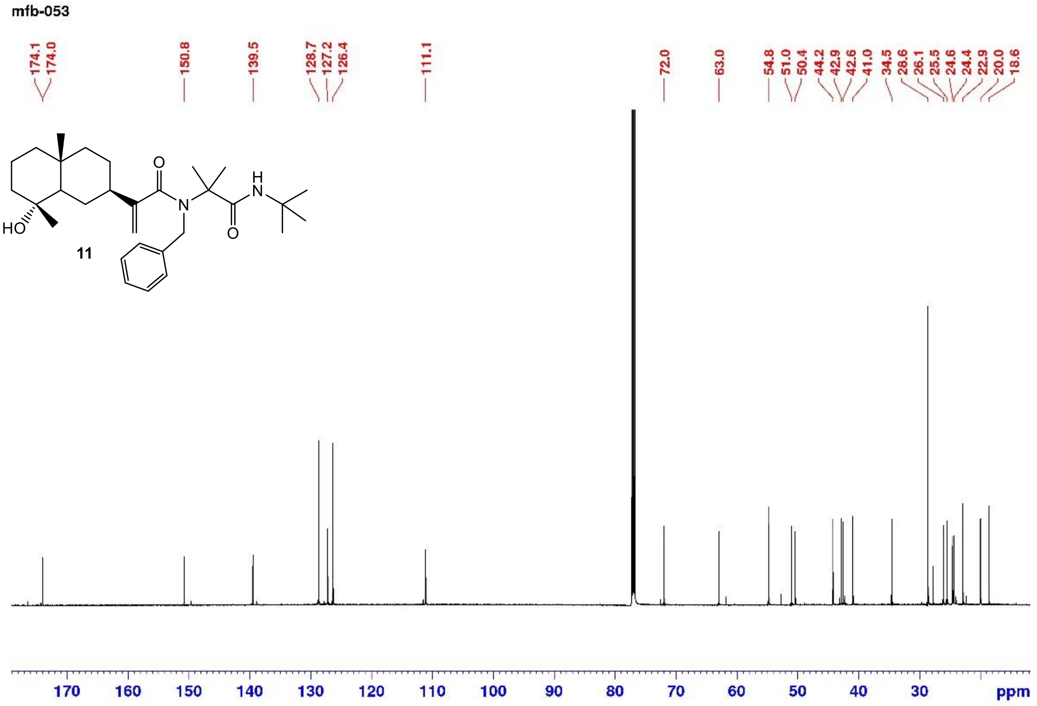

Figure S43. ^{13}C-NMR of **11.**

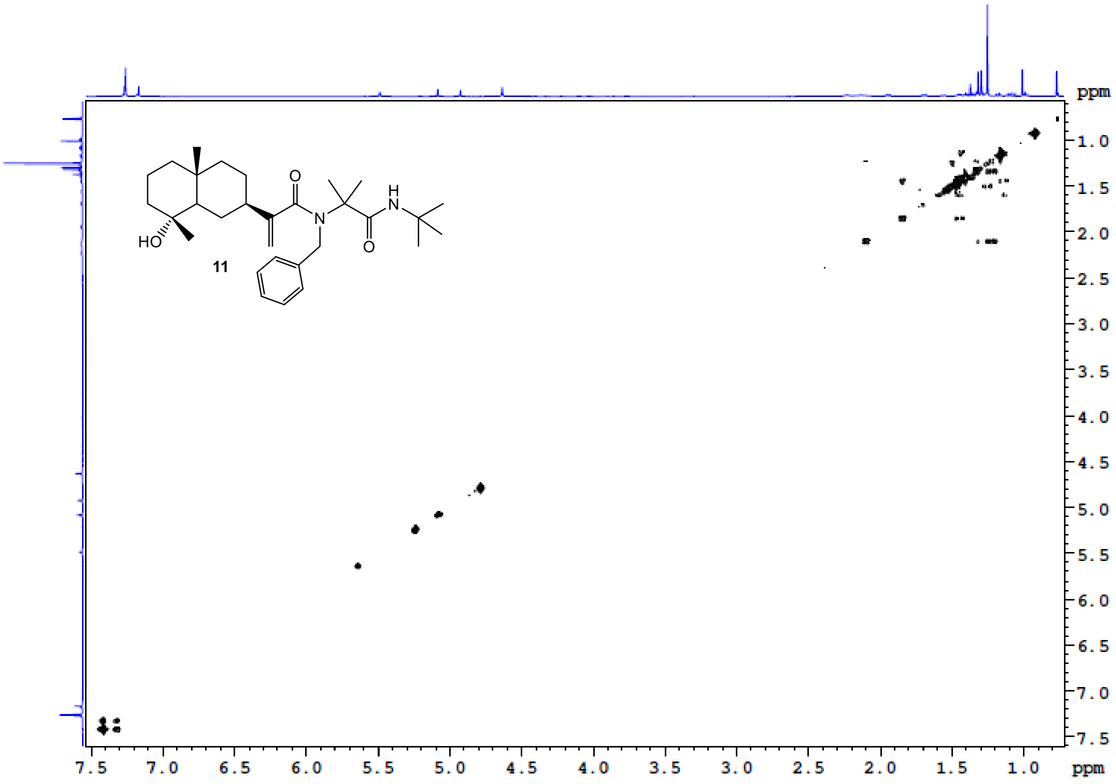

Figure S44. COSY of **11.**

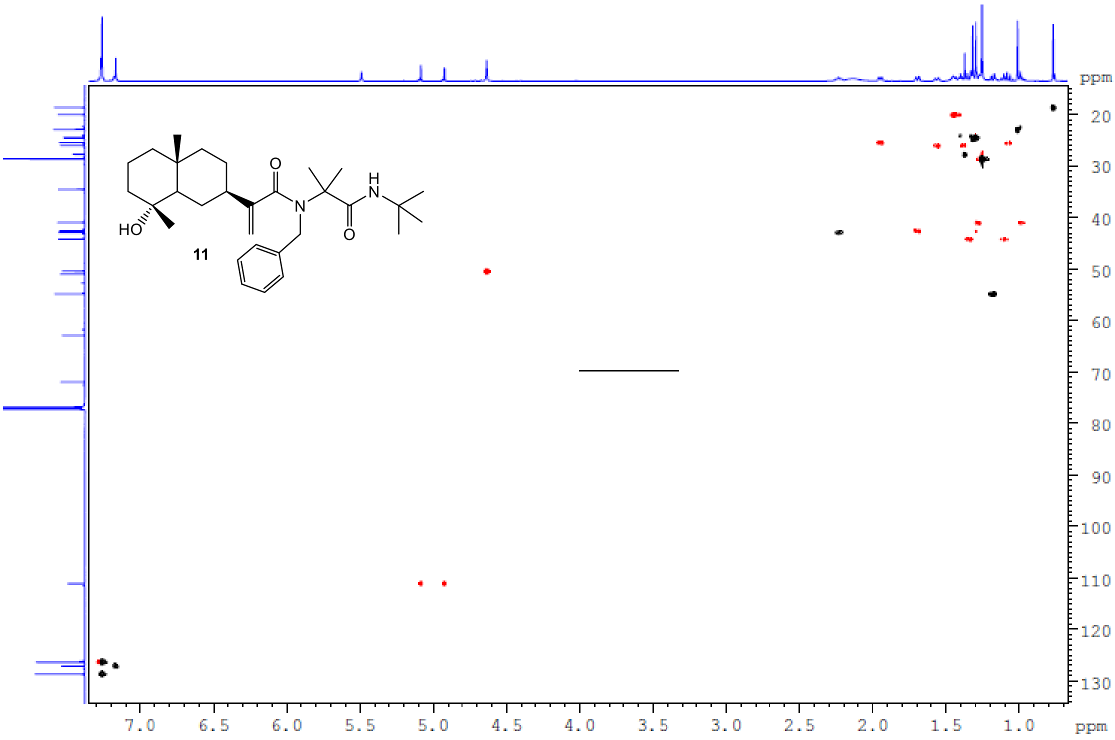

Figure S45. HSQC of **11.**

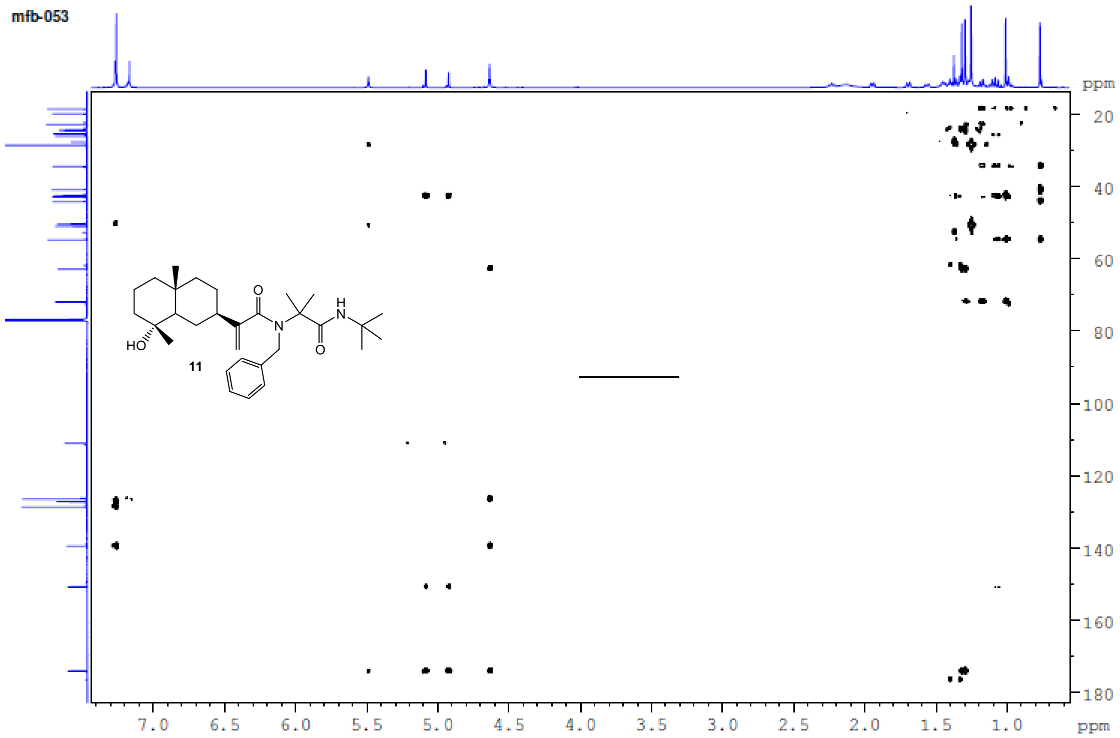

Figure S46. HMBC of **11.**

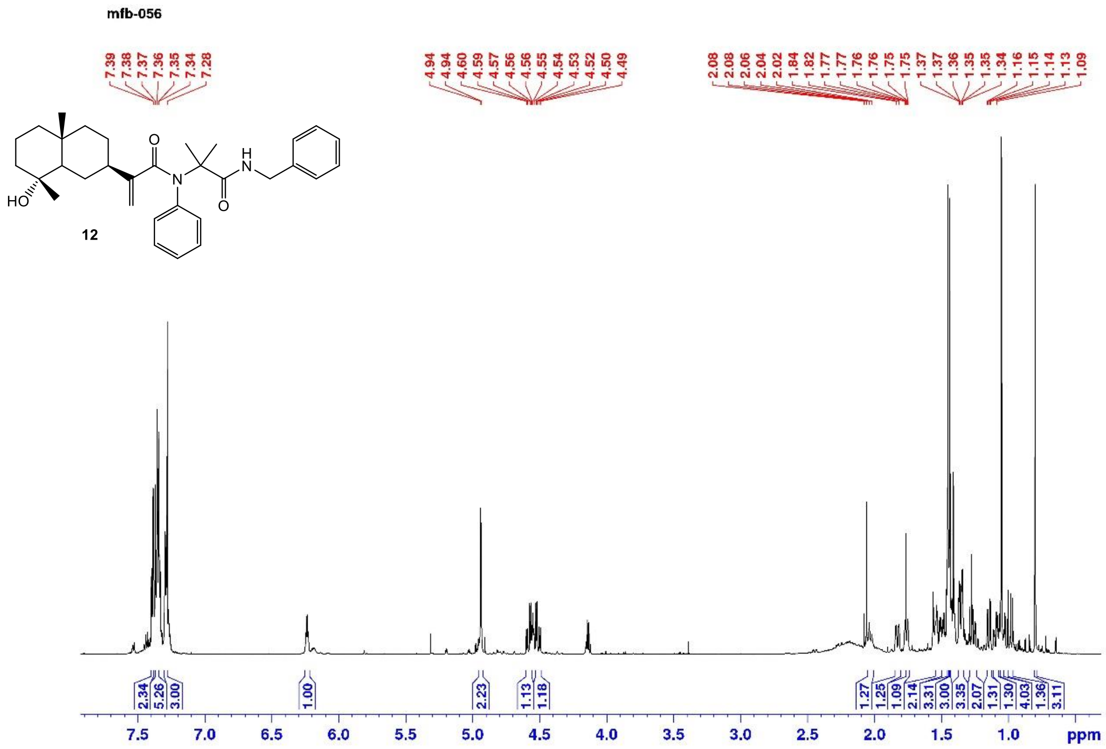

Figure S47. ^{1}H-NMR of **12.**

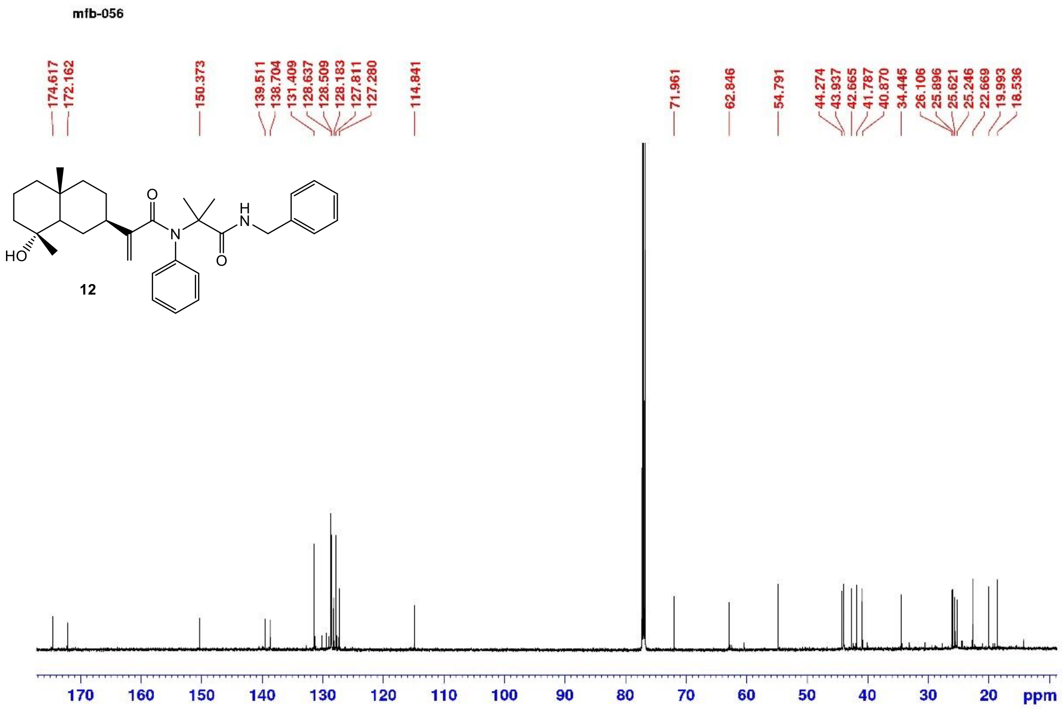

Figure S48. ^{13}C-NMR of **12.**

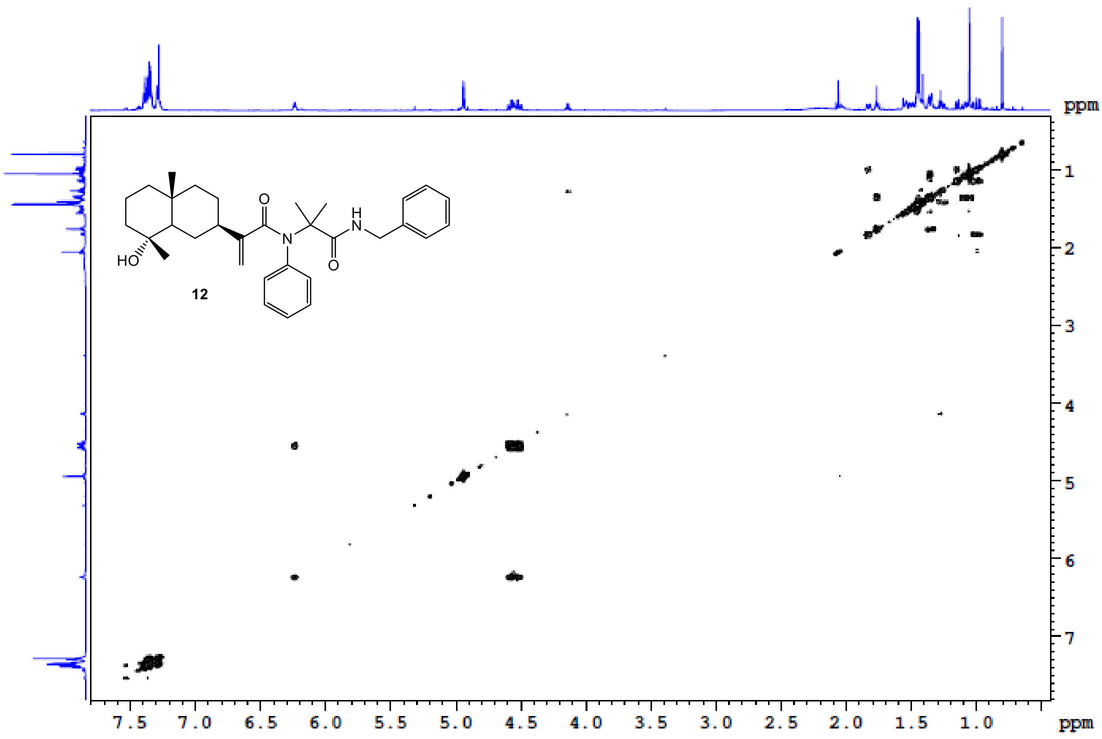

Figure S49. COSY of **12.**

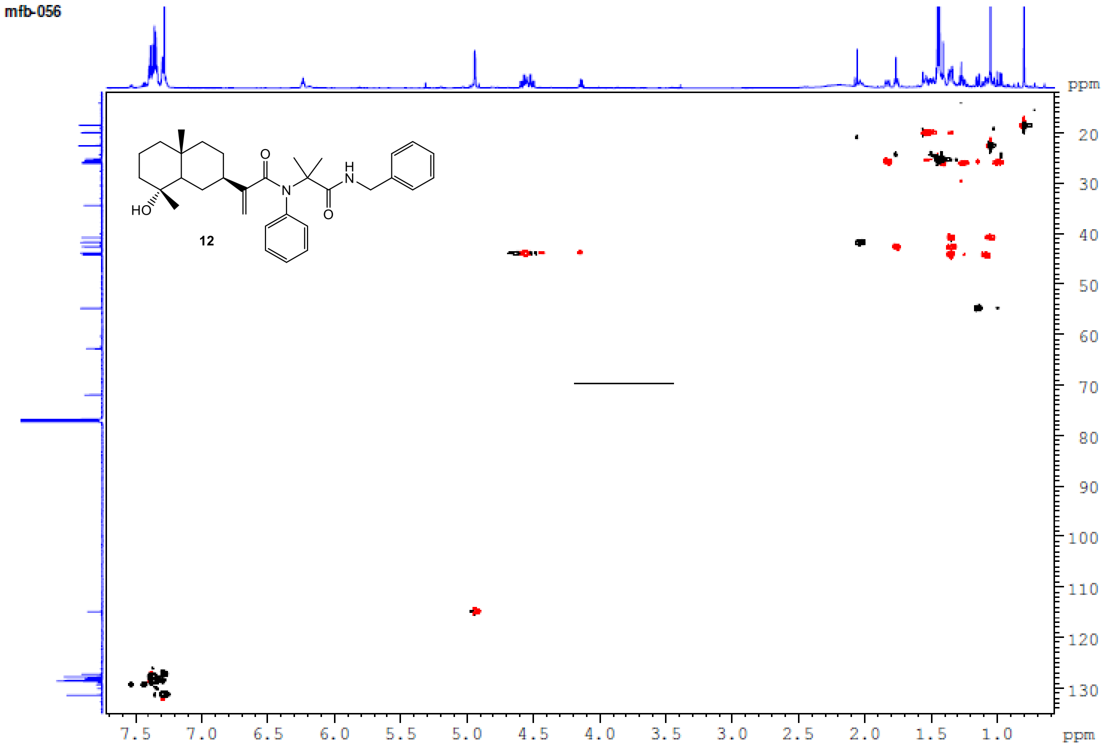

Figure S50. HSQC of **12.**

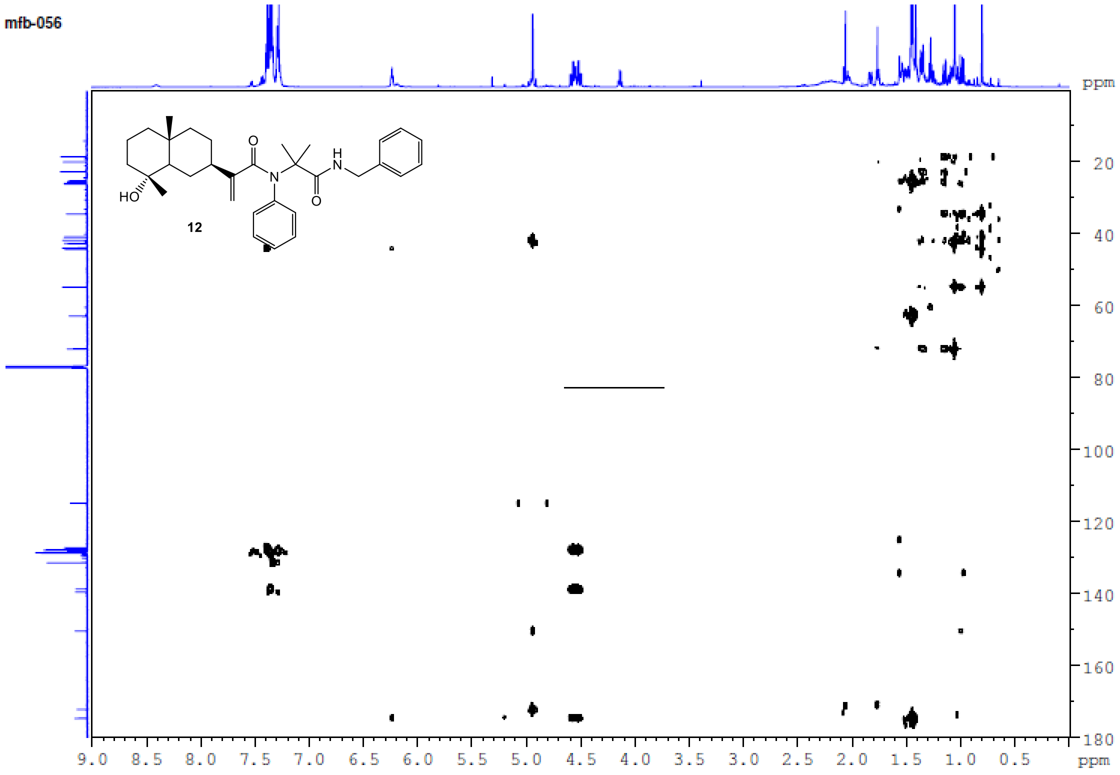

Figure S51. HMBC of **12.**

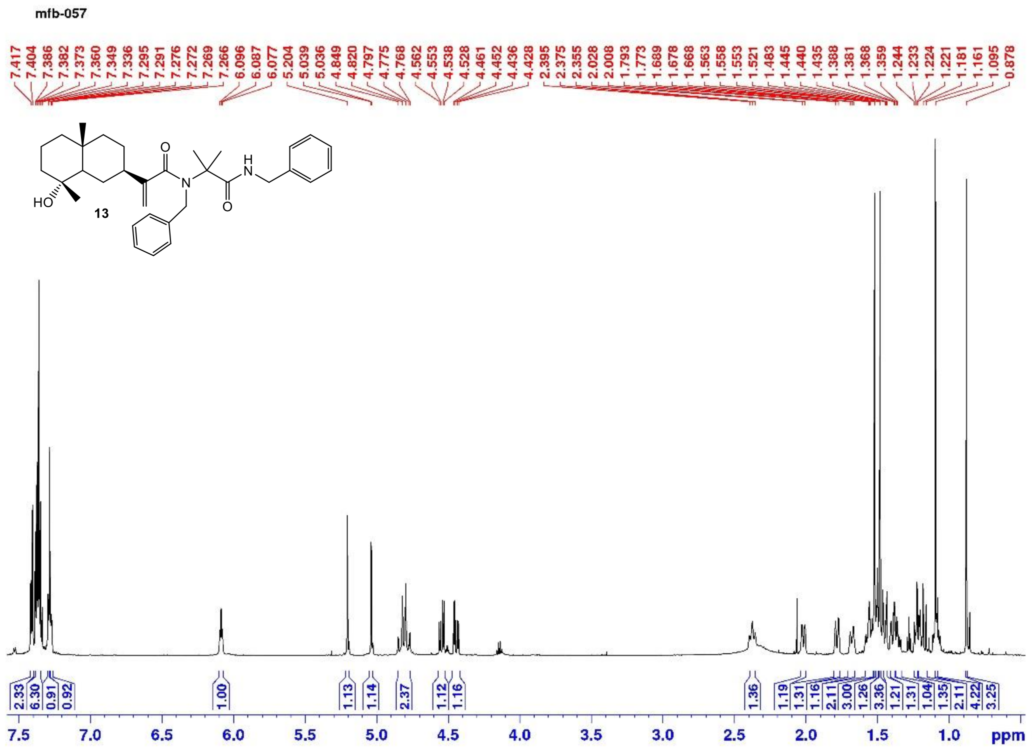

Figure S52. ^{1}H-NMR of **13.**

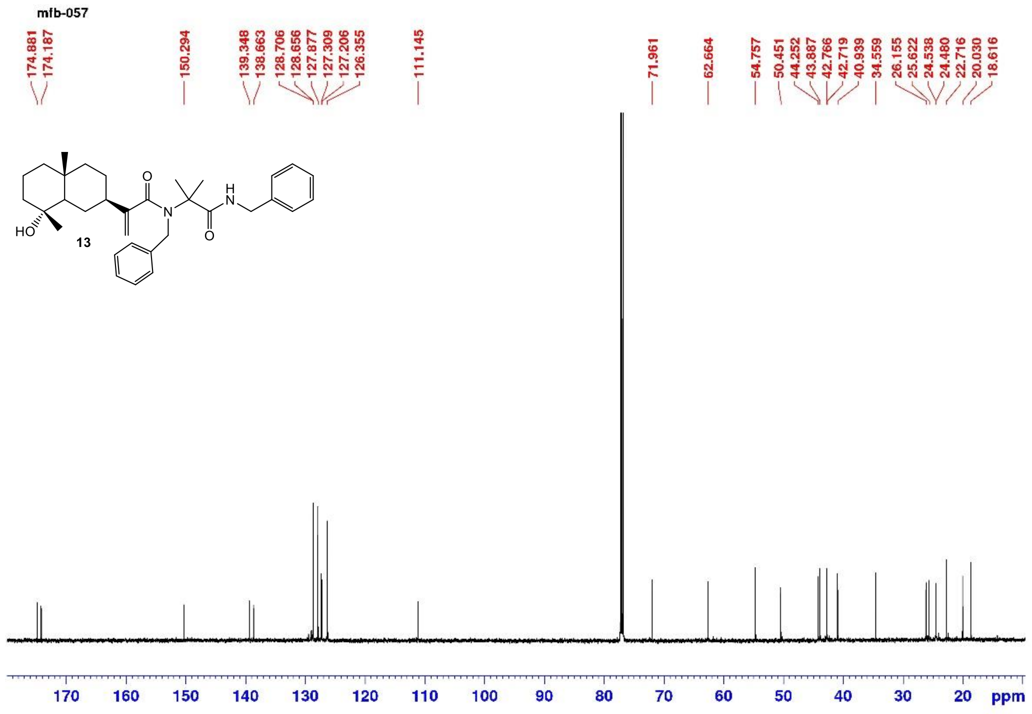

Figure S53. ^{13}C-NMR of **13.**

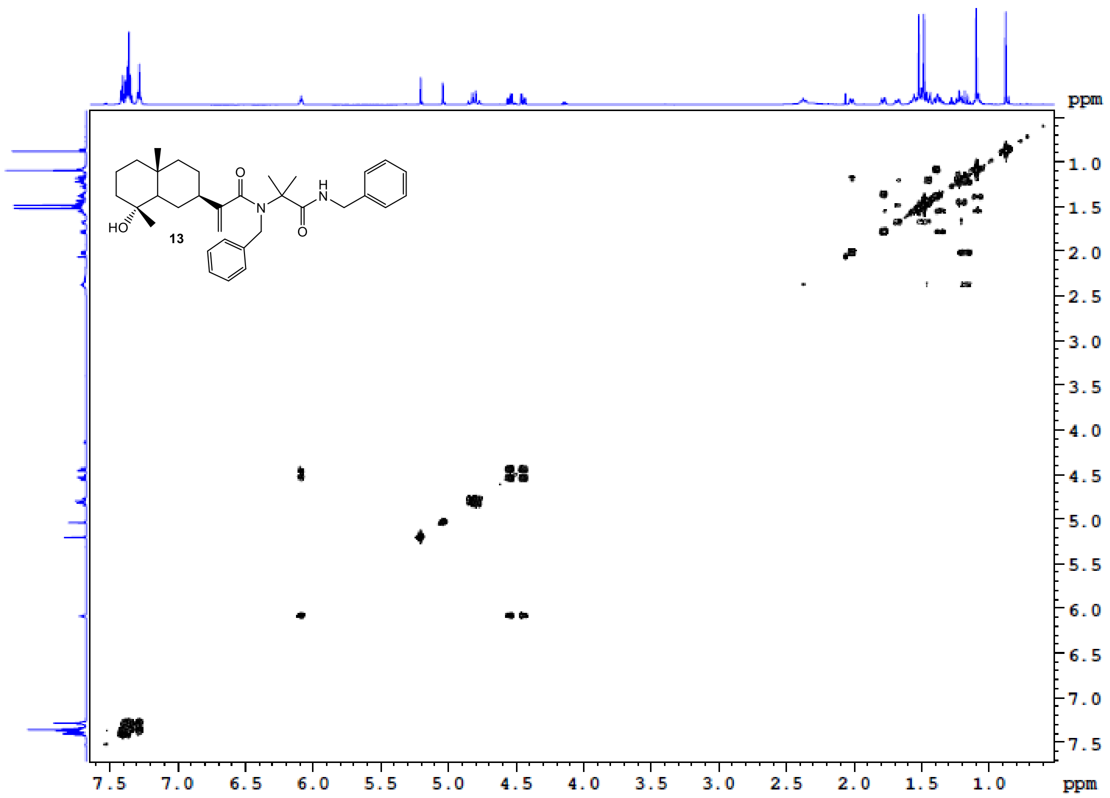

Figure S54. COSY of **13.**

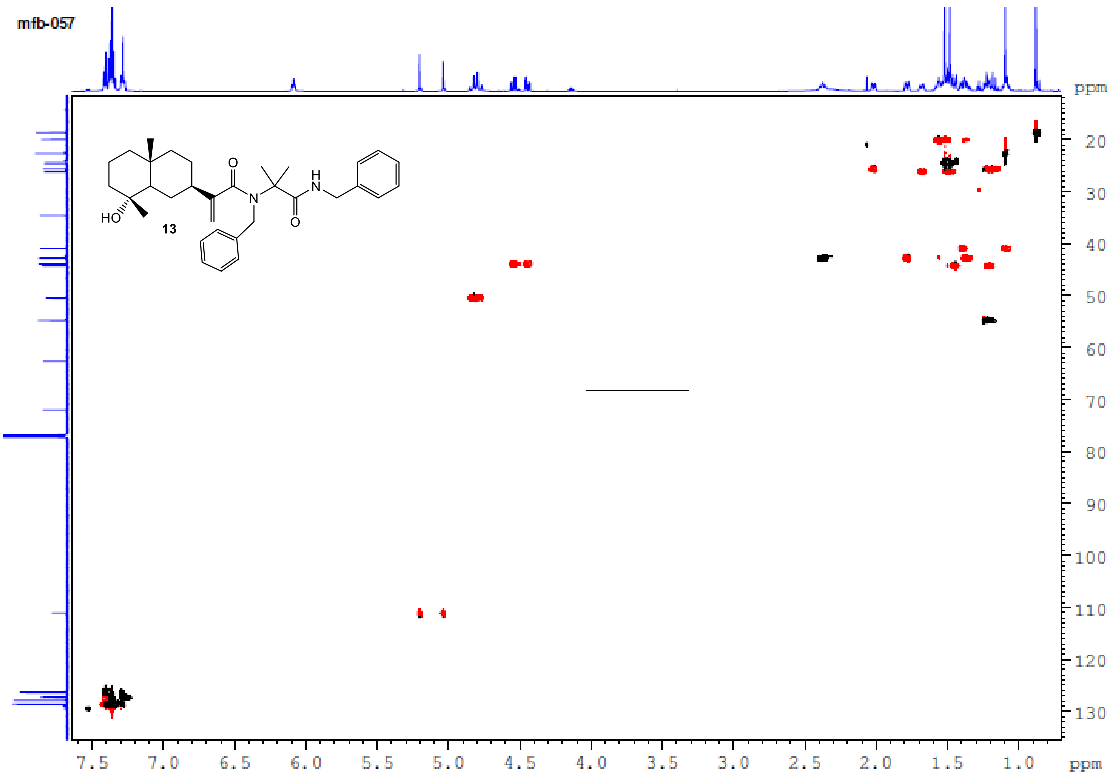

Figure S55. HSQC of **13.**

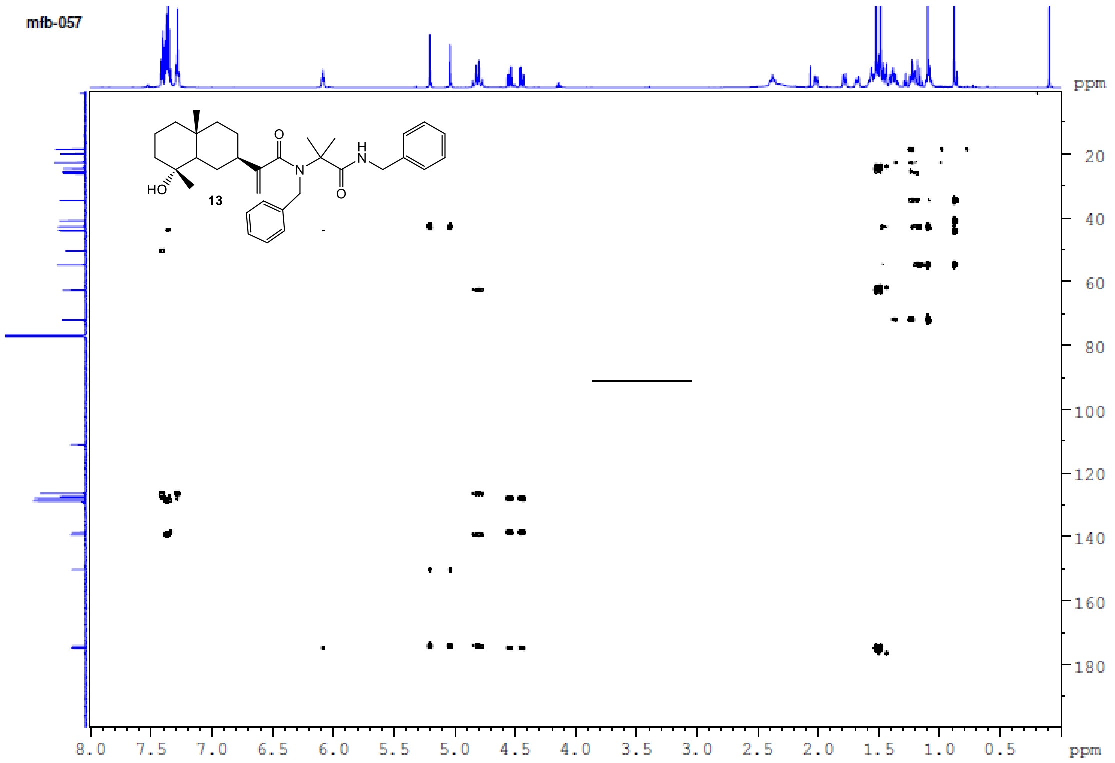

Figure S56. HMBC of **13.**

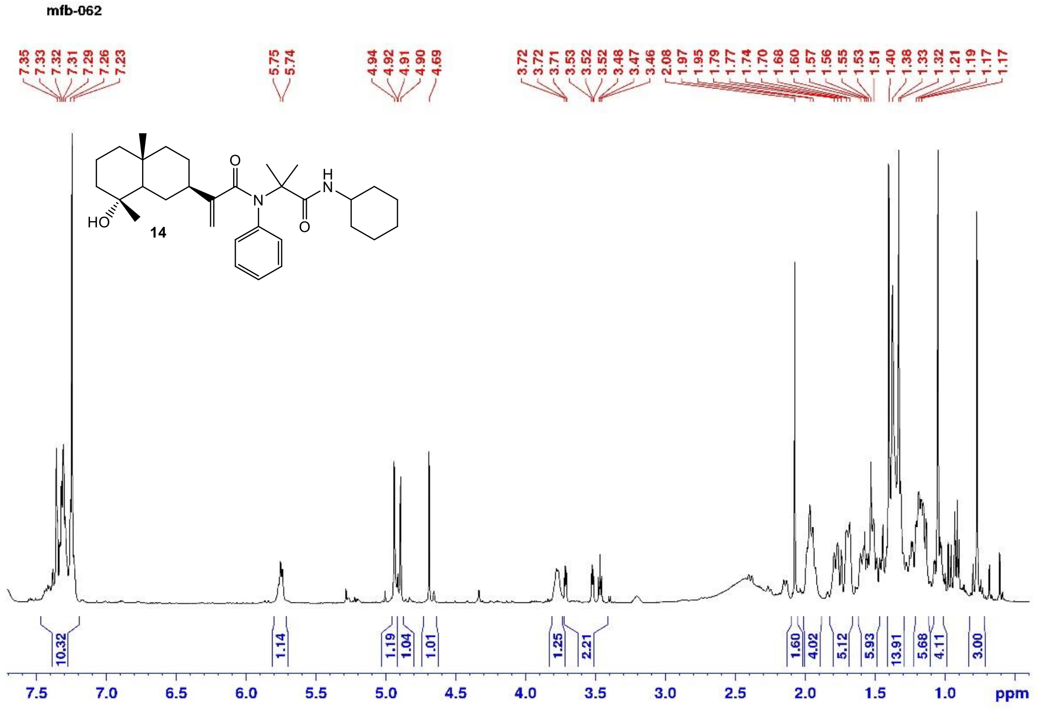

Figure S57. ^{1}H-NMR of **14.**

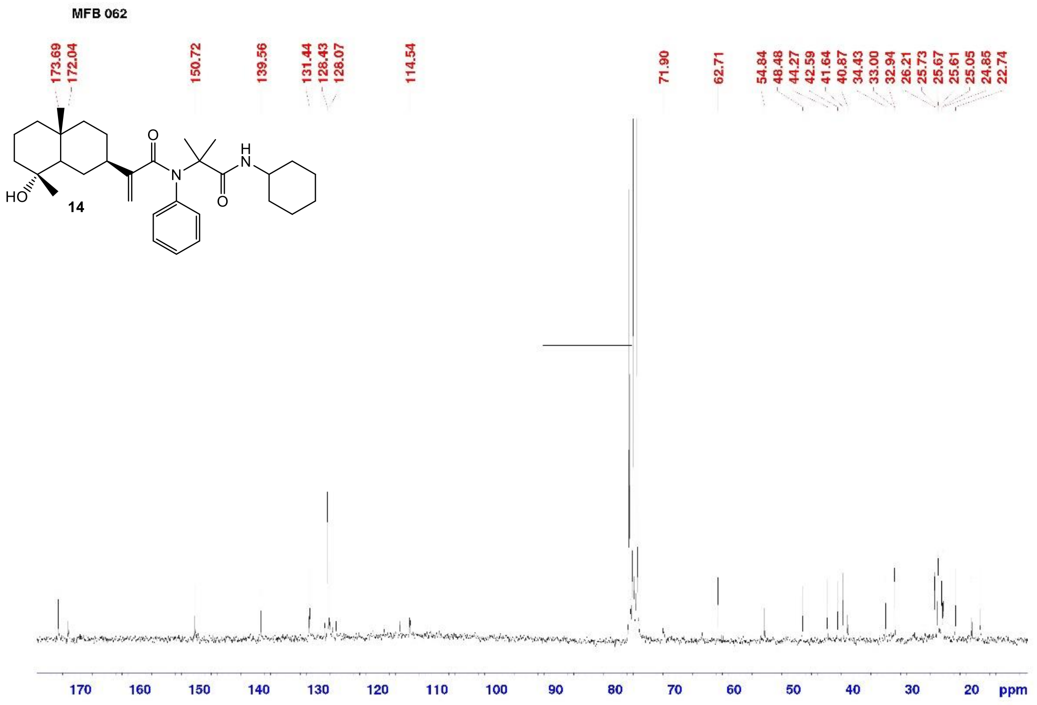

Figure S58. ^{13}C-NMR of **14.**

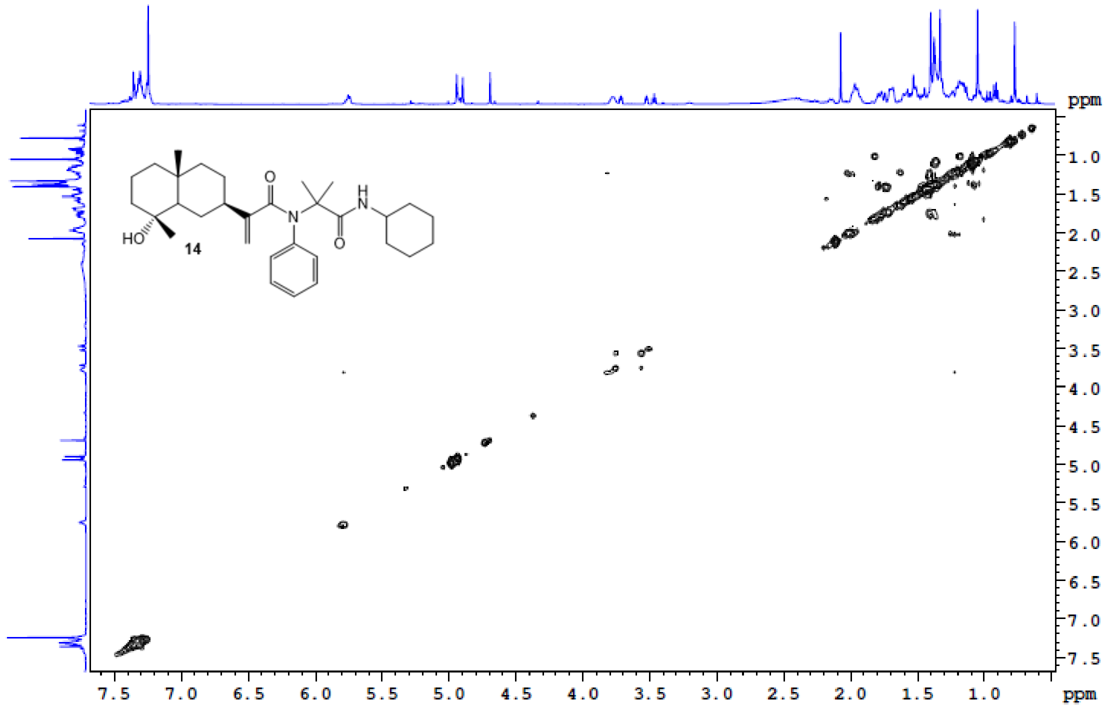

Figure S59. COSY of **14.**

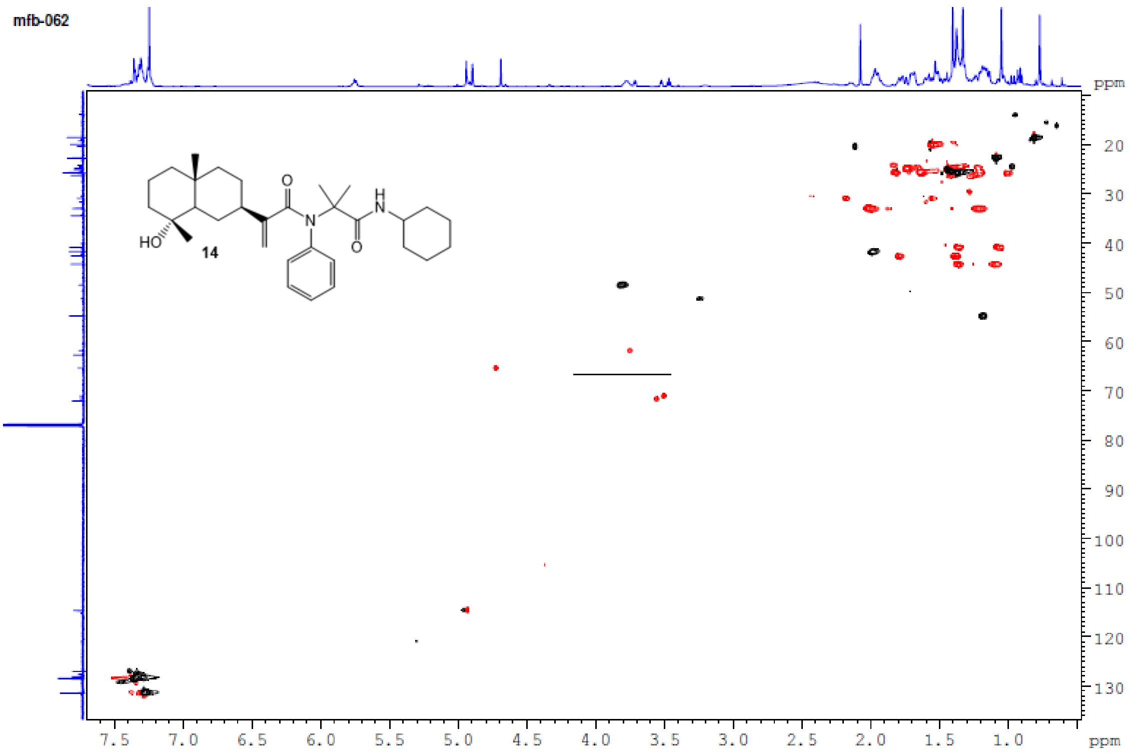

Figure S60. HSQC of **14.**

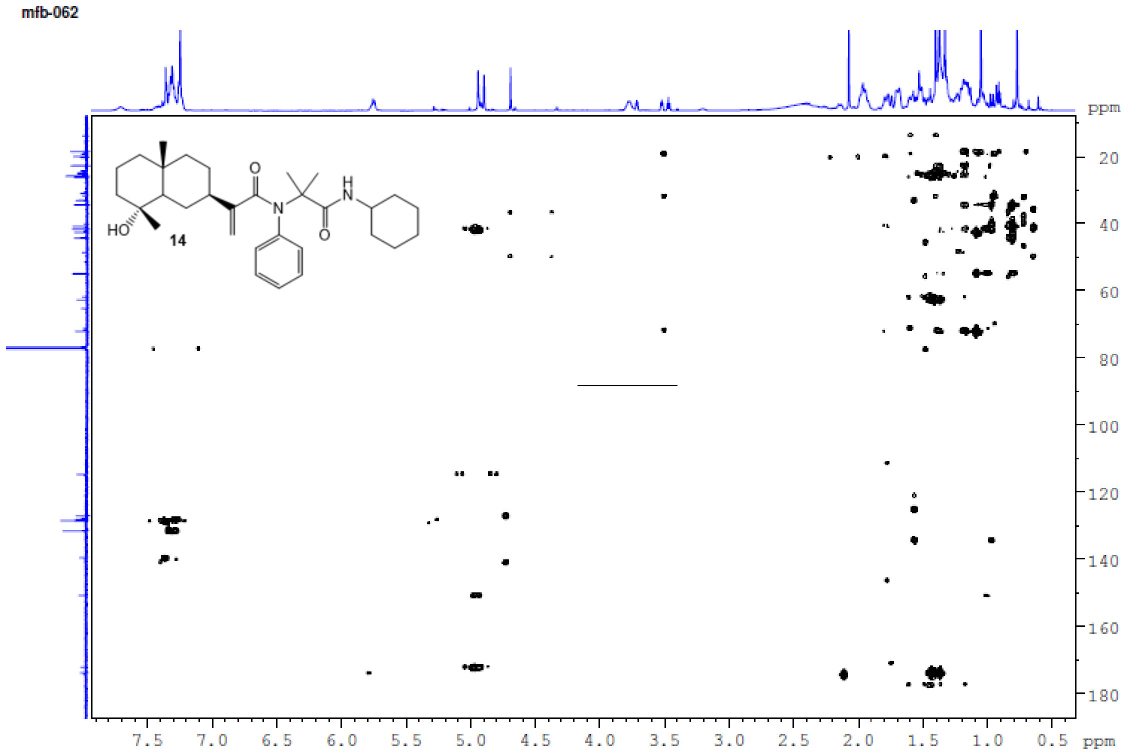

Figure S61. HMBC of **14.**

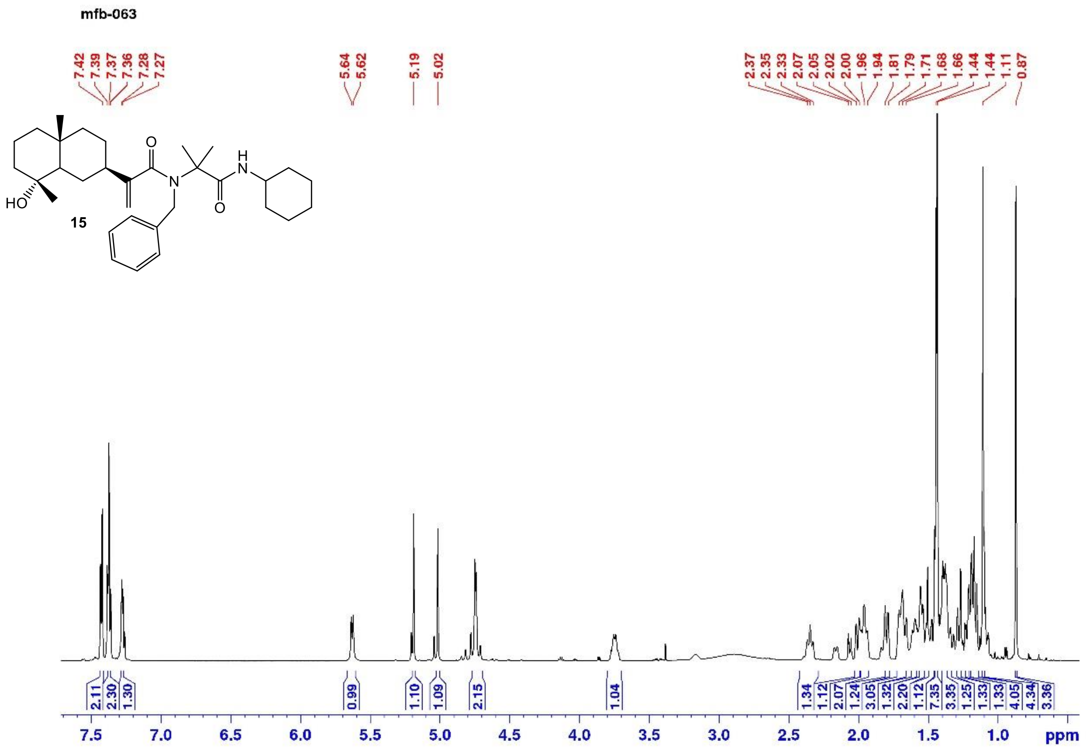

Figure S62. ^{1}H-NMR of **15.**

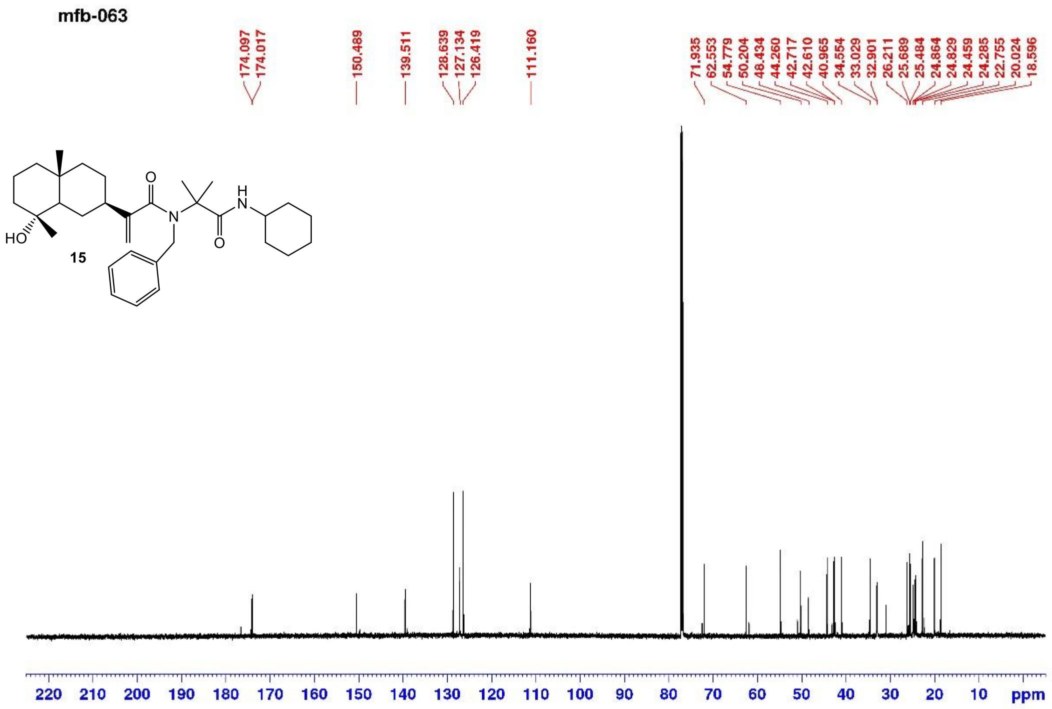

Figure S63. ^{13}C-NMR of **15.**

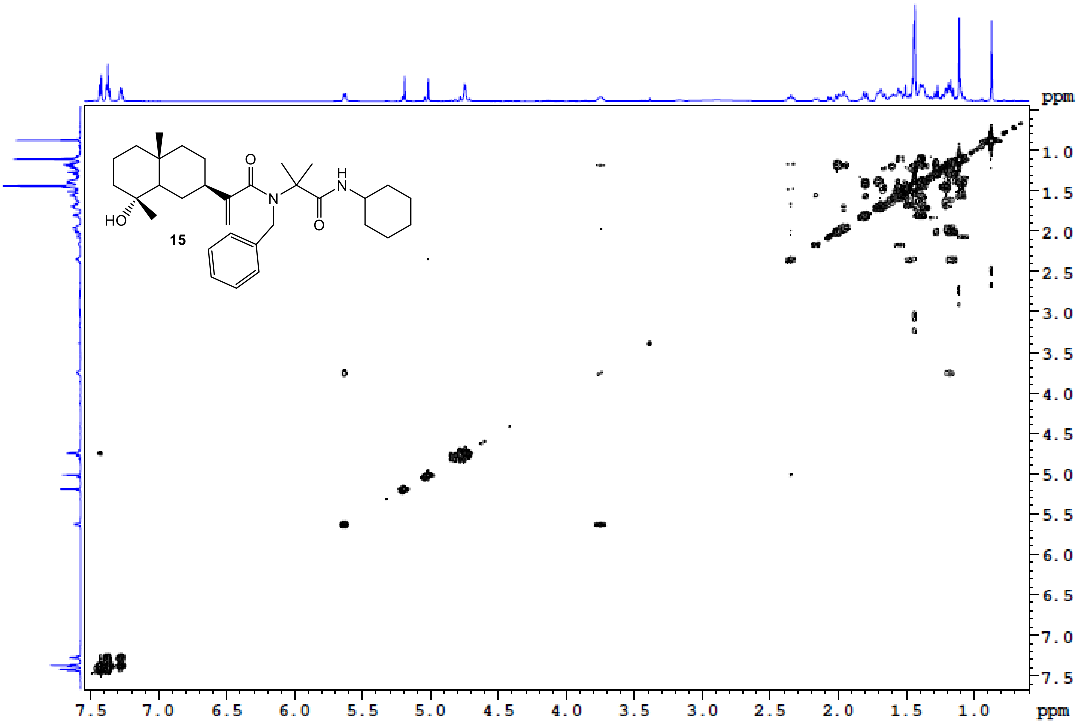

Figure S64. COSY of **15.**

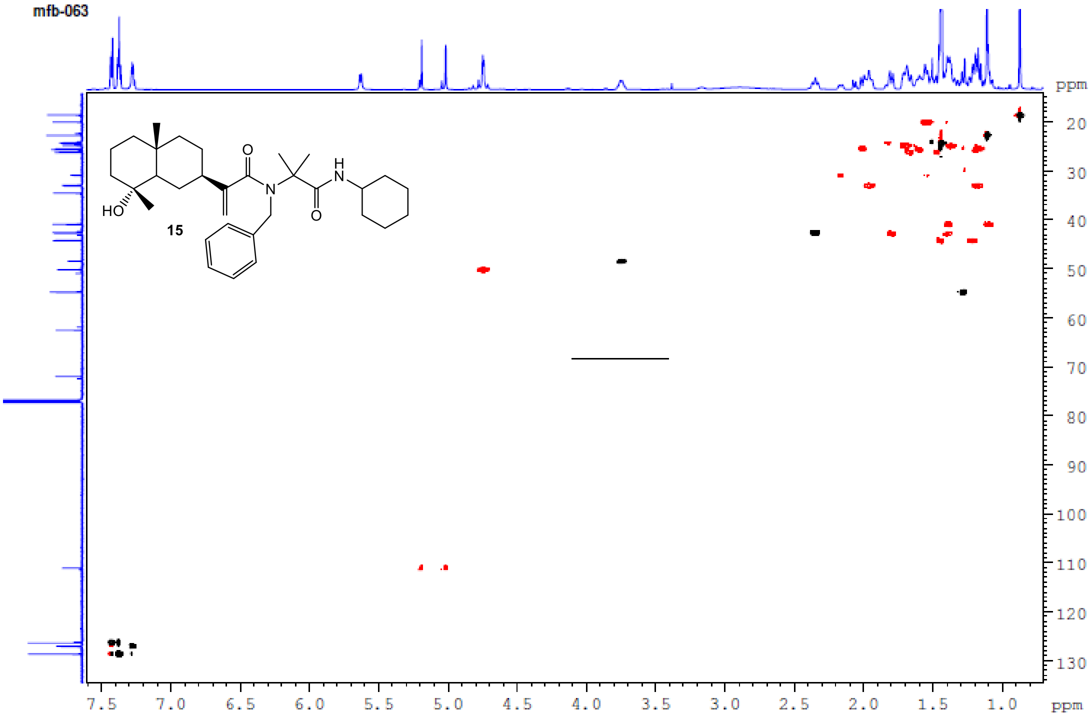

Figure S65. HSQC of **15.**

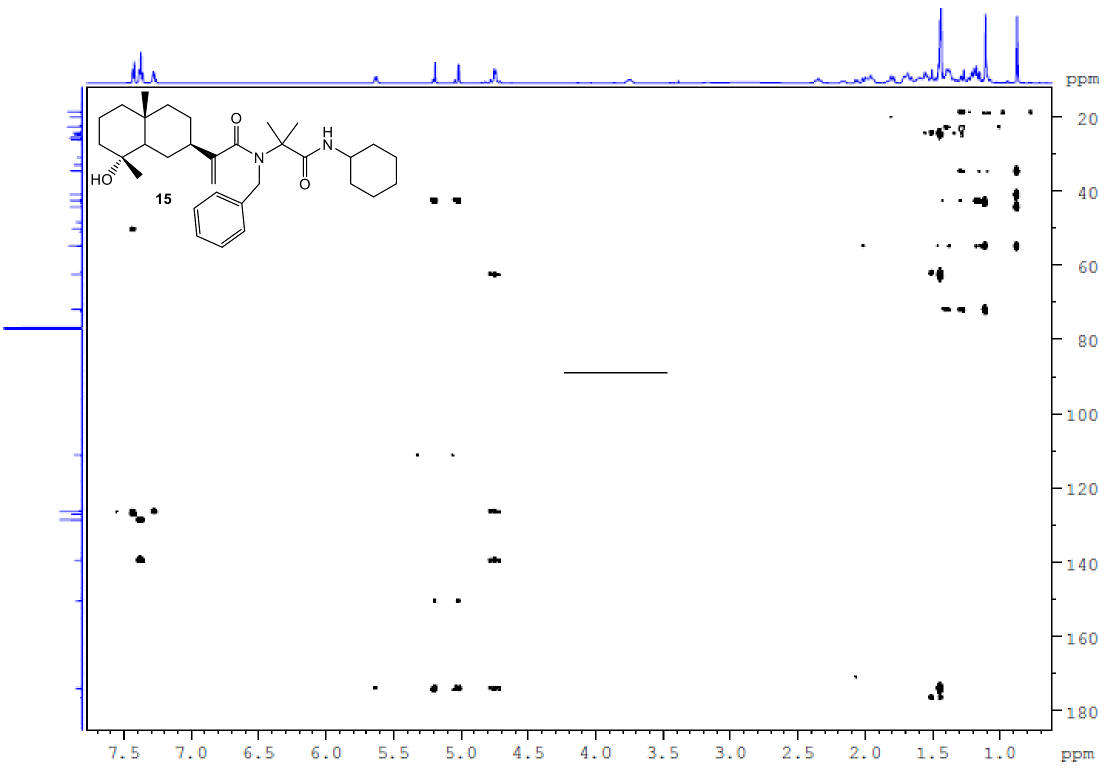

Figure S66. HMBC of **15.**

4. NMR spectra of Ilicic alcohol derivatives

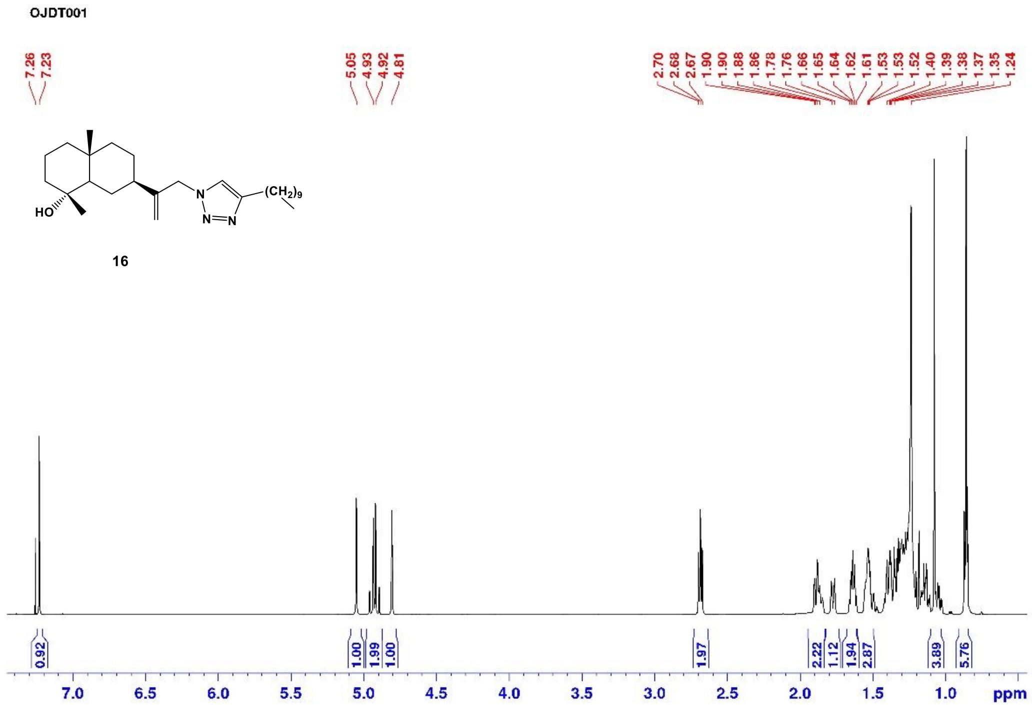

Figure S67. ^{1}H-NMR of **16.**

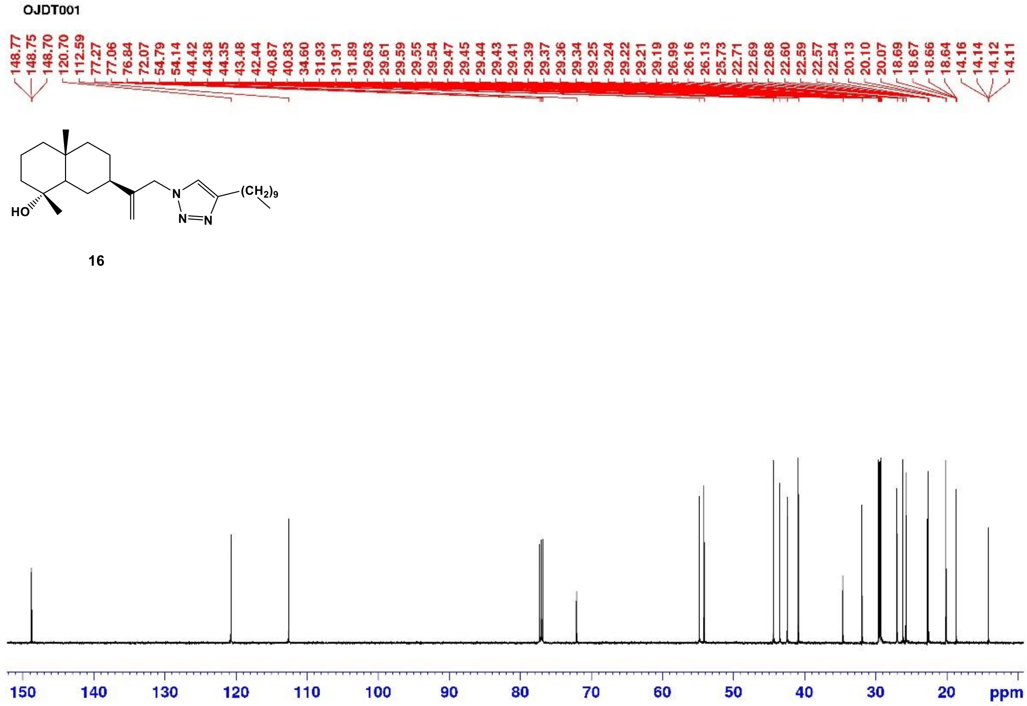

Figure S68. ^{13}C-NMR of **16.**

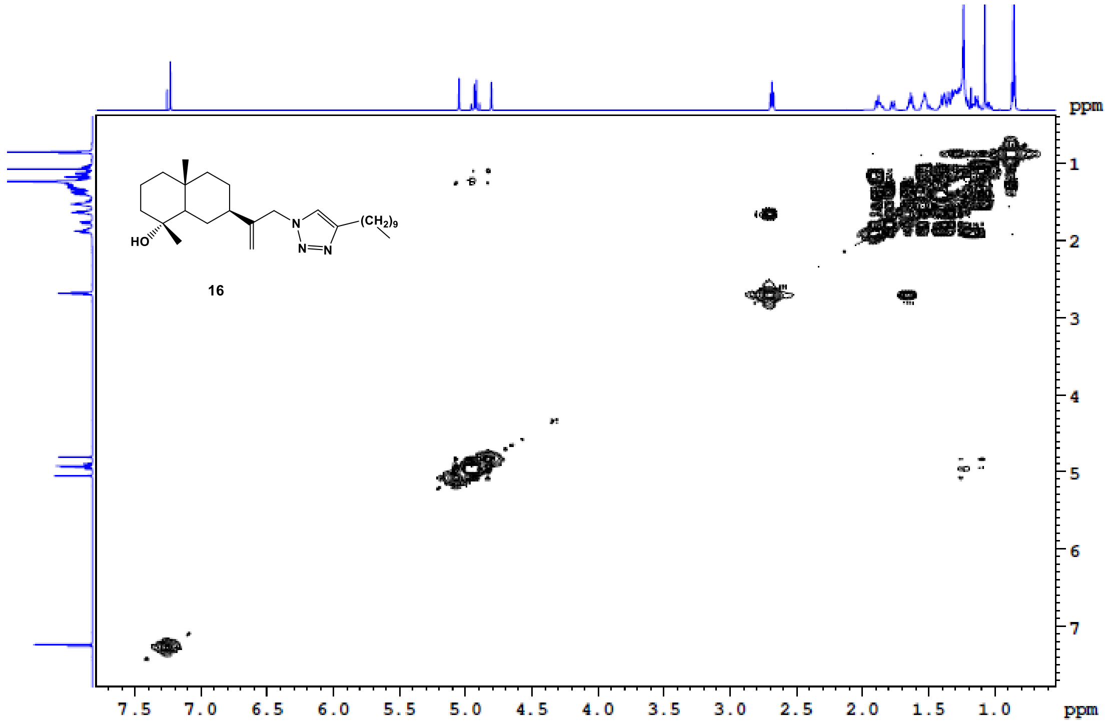

Figure S69. COSY of **16.**

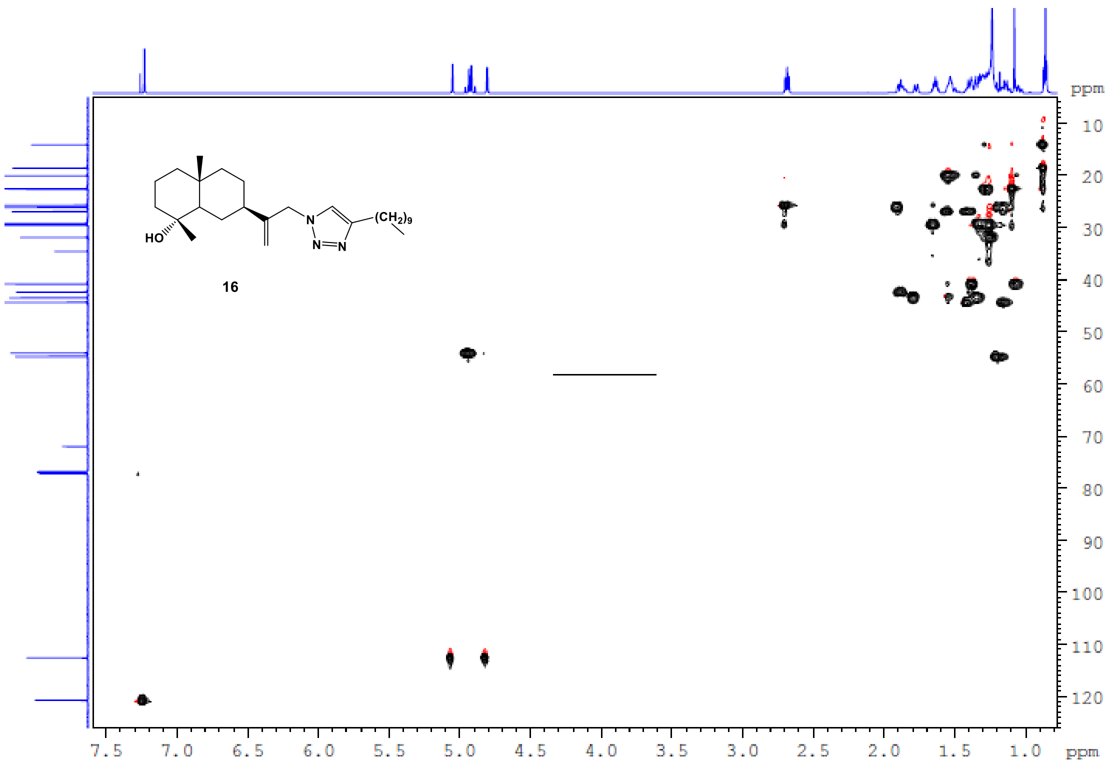

Figure S70. HSQC of **16.**

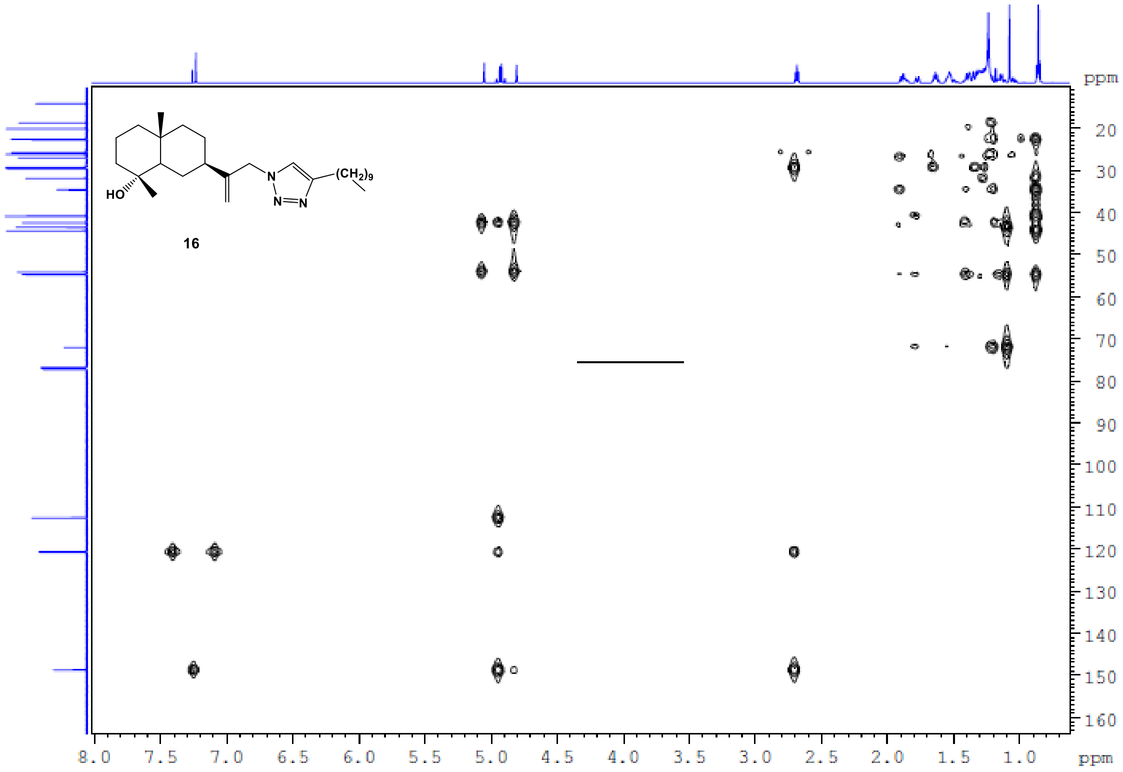

Figure S71. HMBC of **16.**

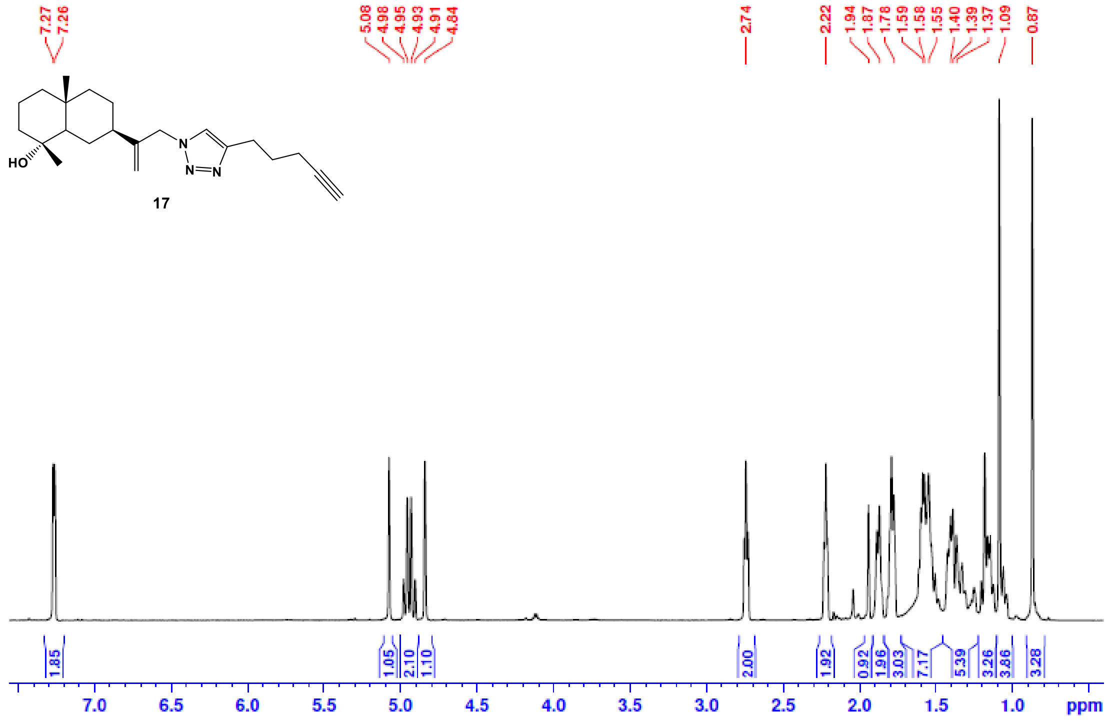

Figure S72. ^{1}H-NMR of **17.**

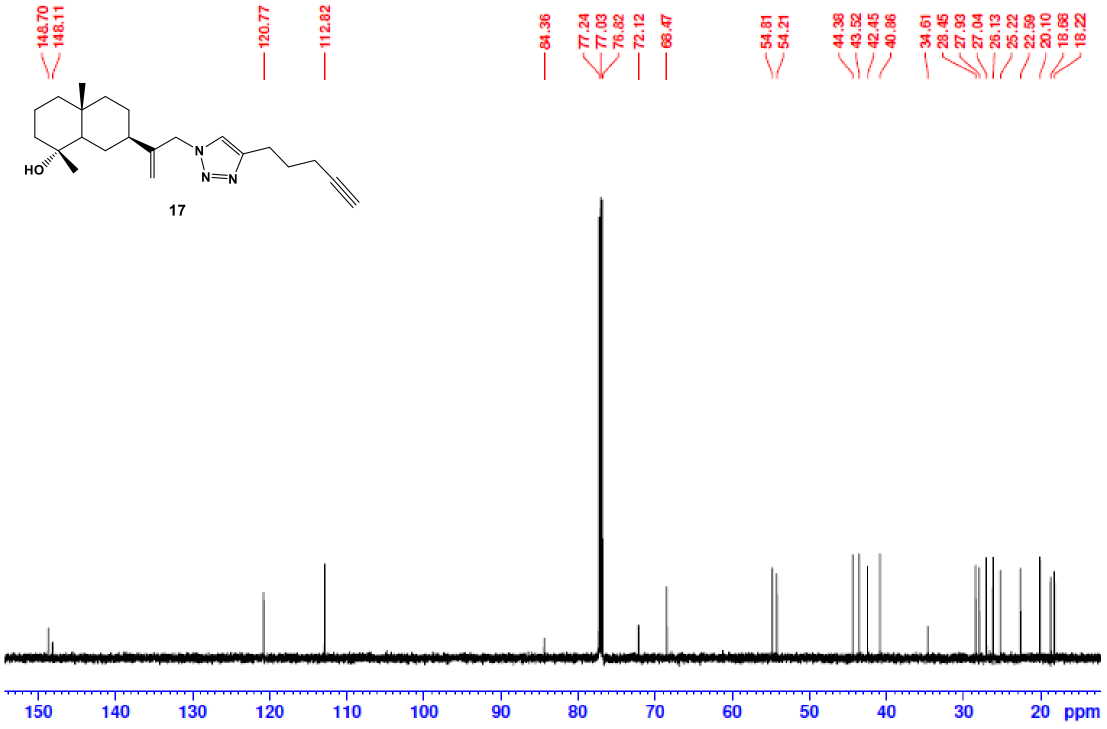

Figure S73. ^{13}C-NMR of **17.**

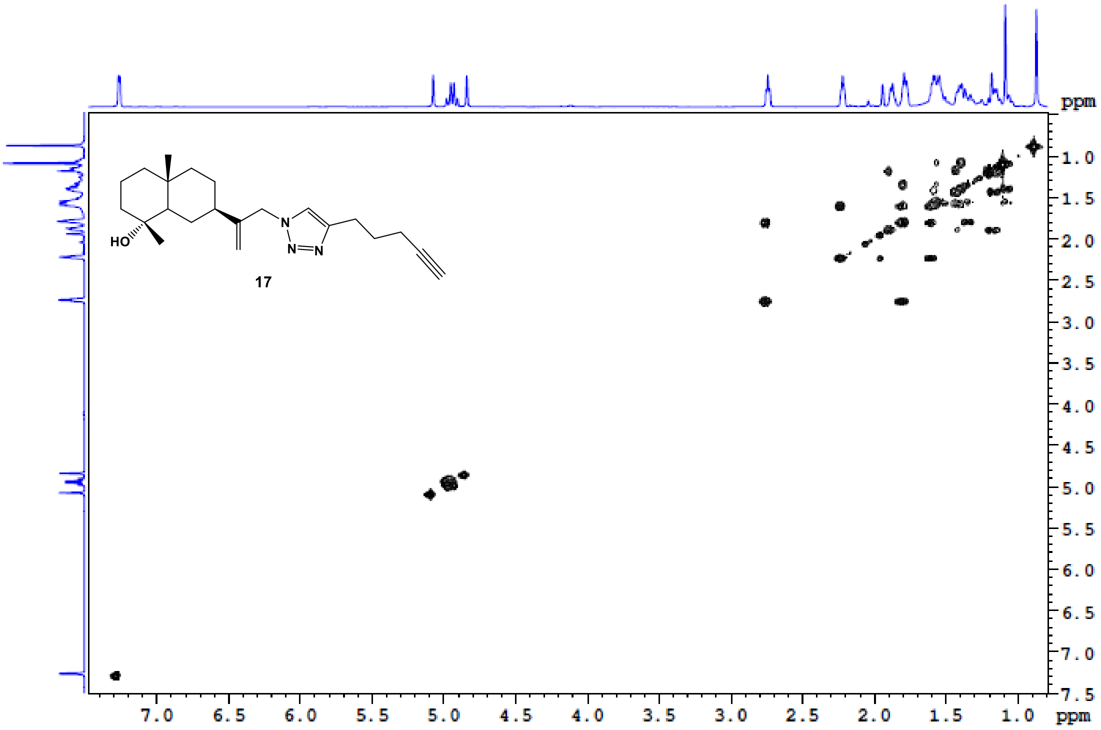

Figure S74. COSY of **17.**

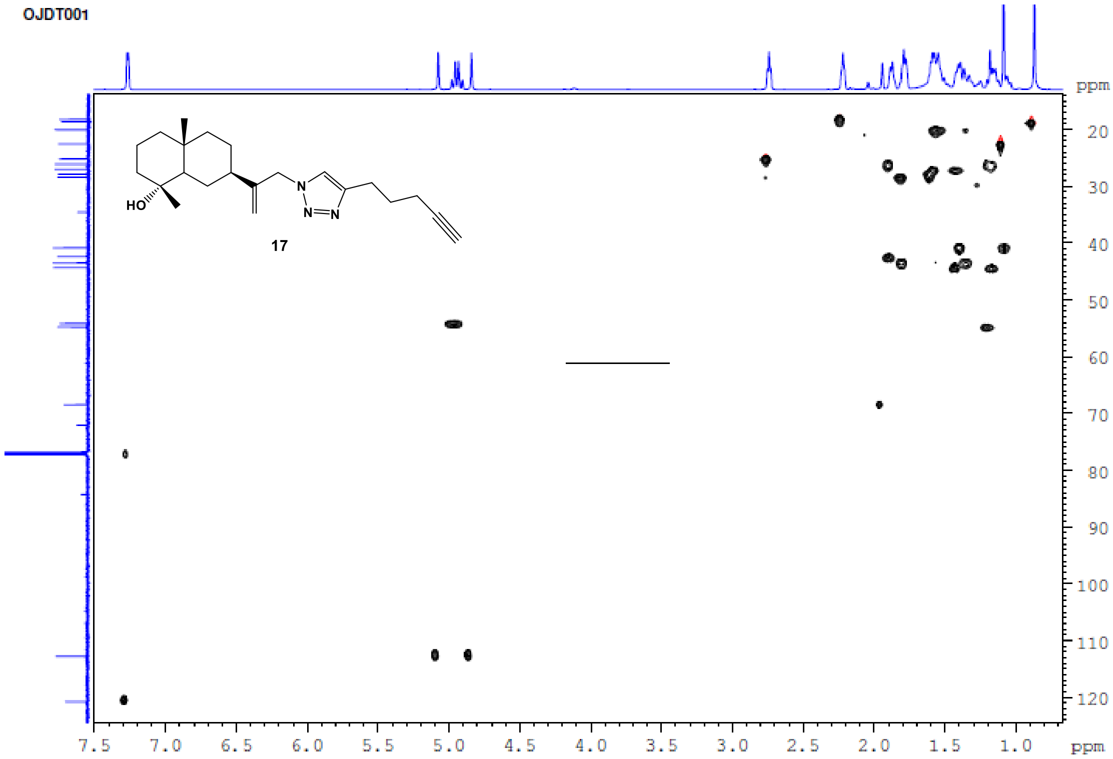

Figure S75. HSQC of **17.**

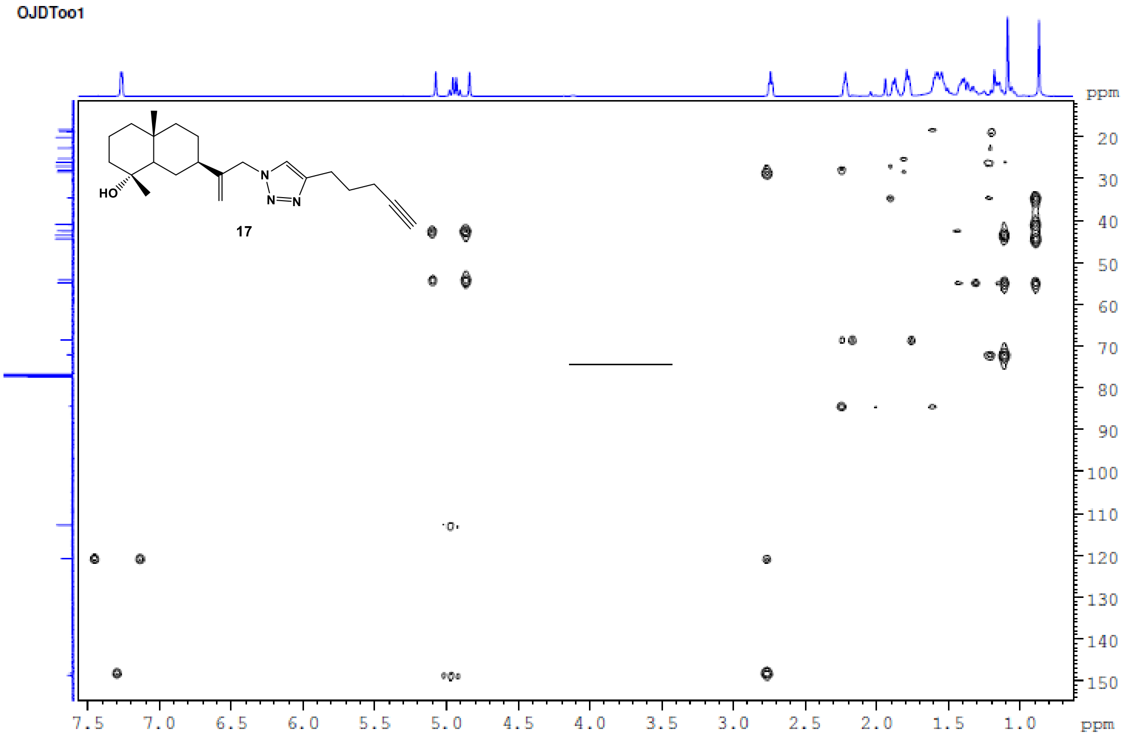

Figure S76. HMBC of **17.**

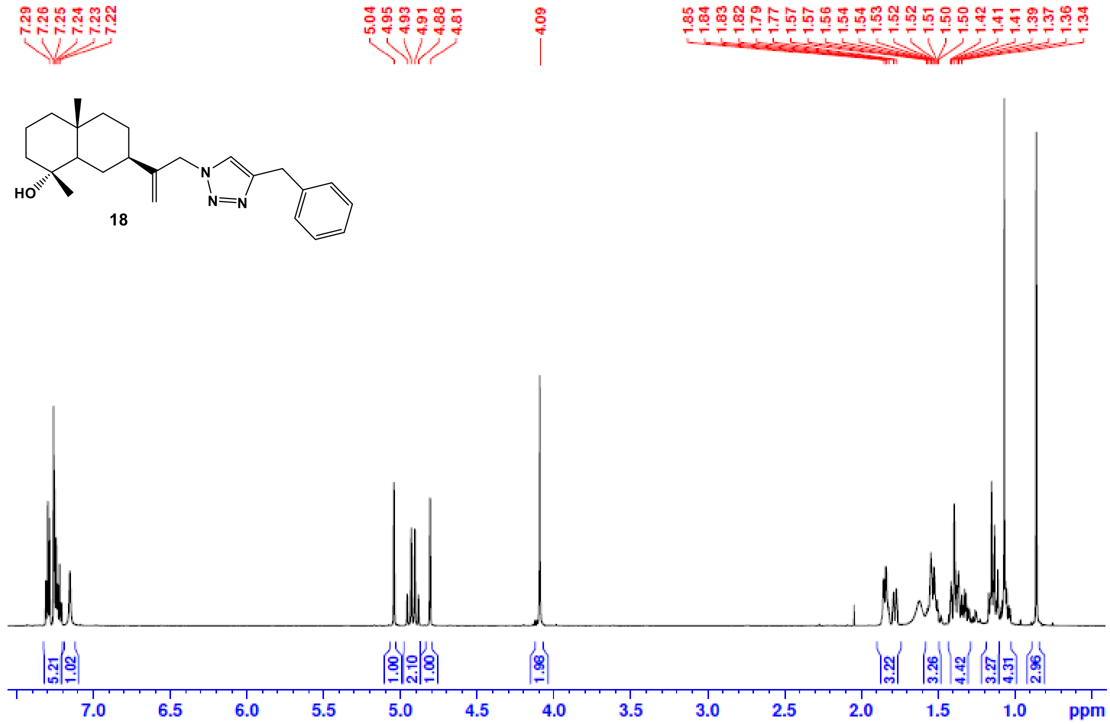

Figure S77. ¹H-NMR of **18.**

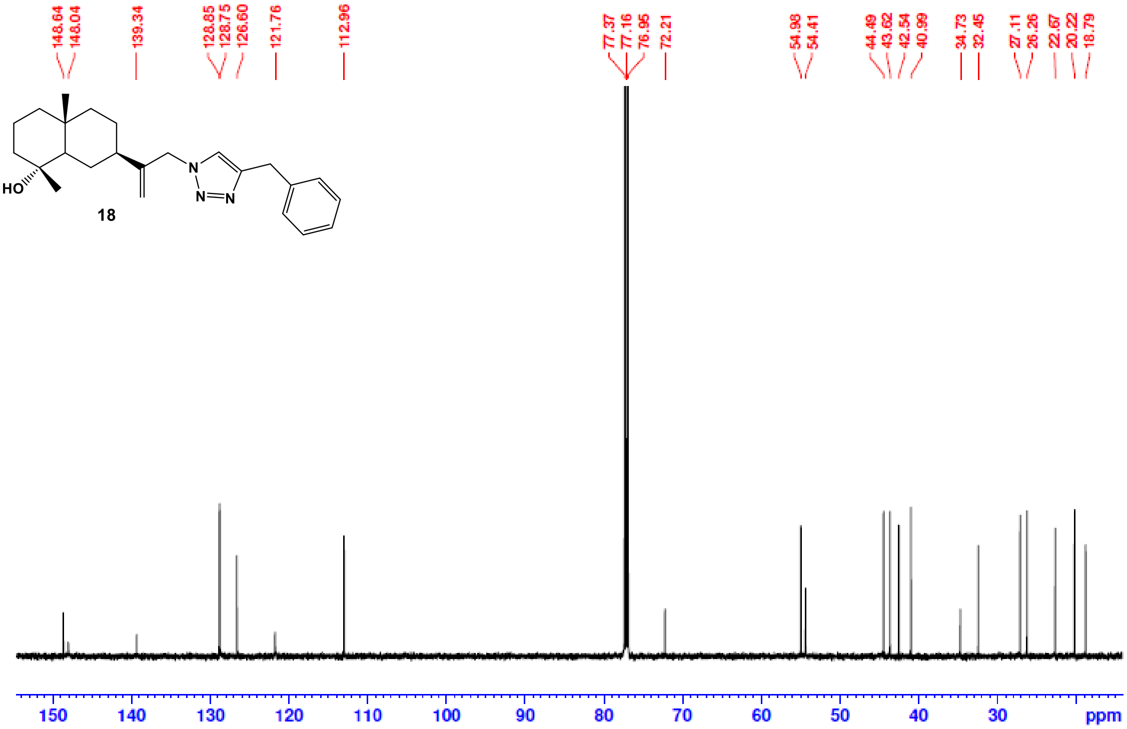

Figure S78. ^{13}C-NMR of **18.**

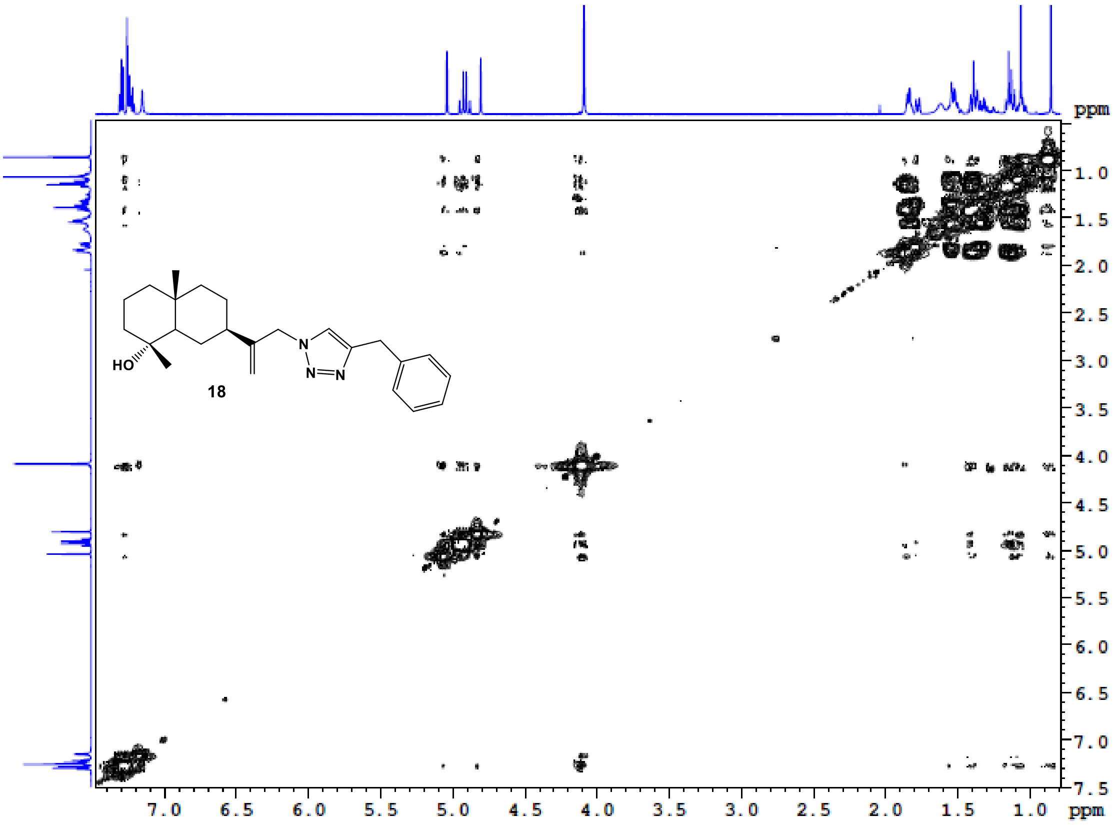

Figure S79. COSY of **18.**

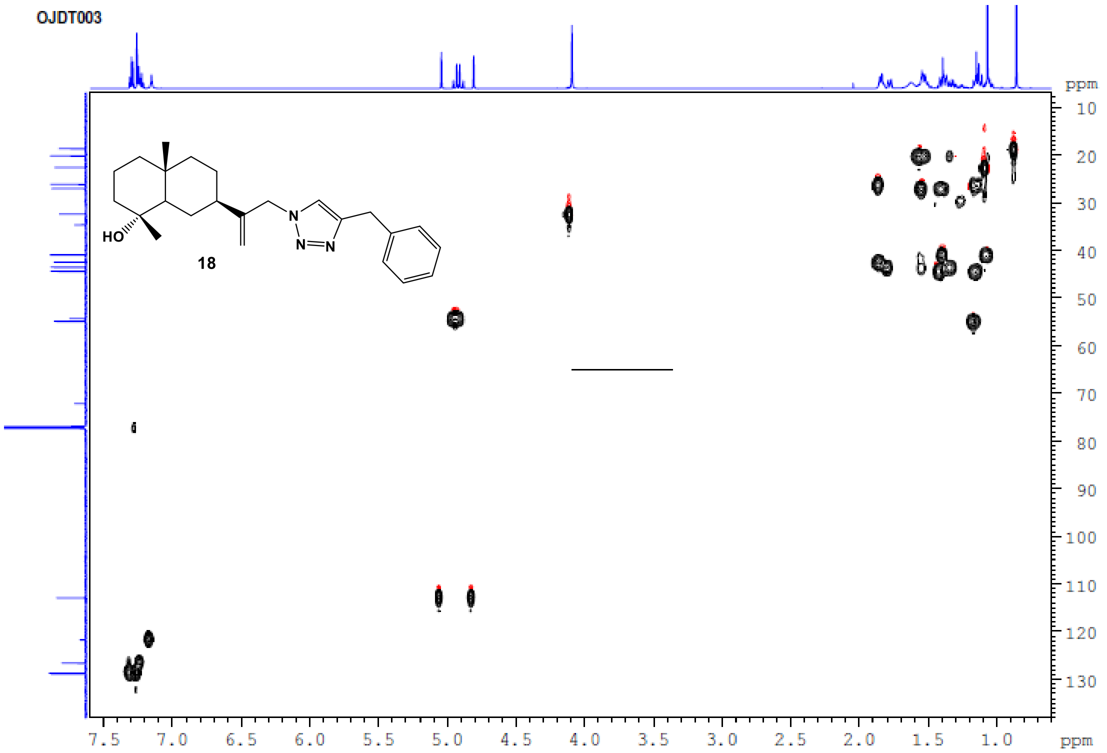

Figure S80. HSQC of **18.**

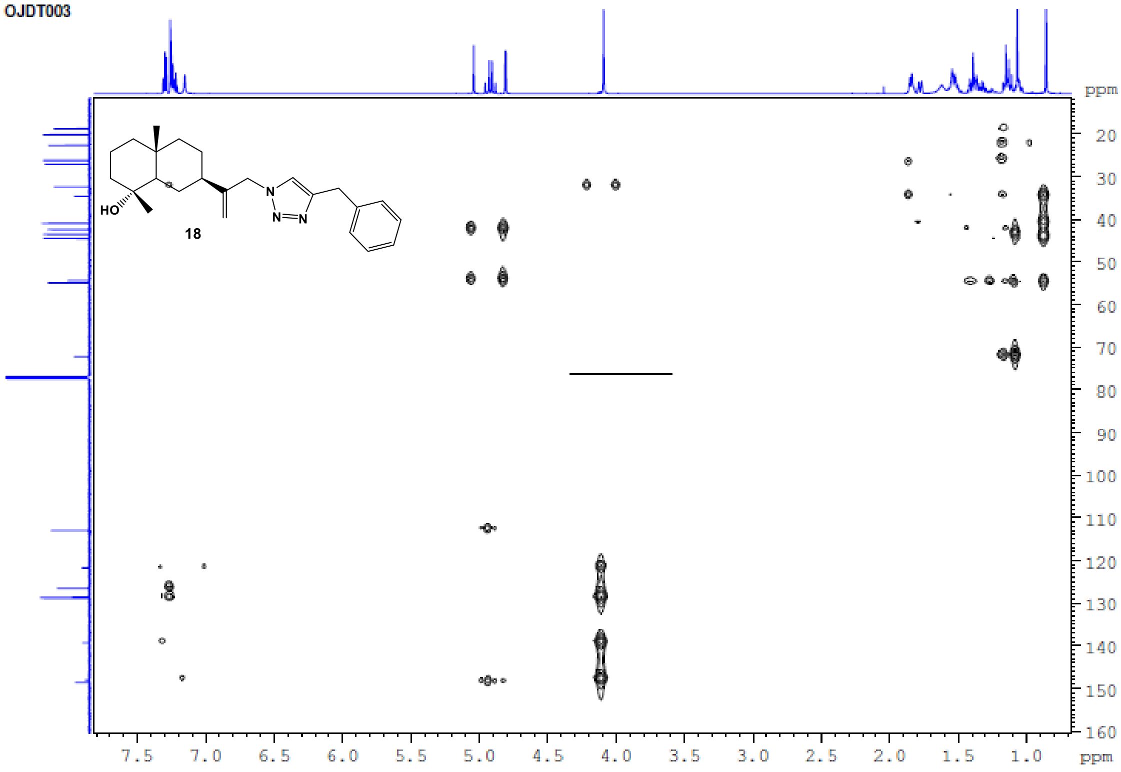

Figure S81. HMBC of **18.**

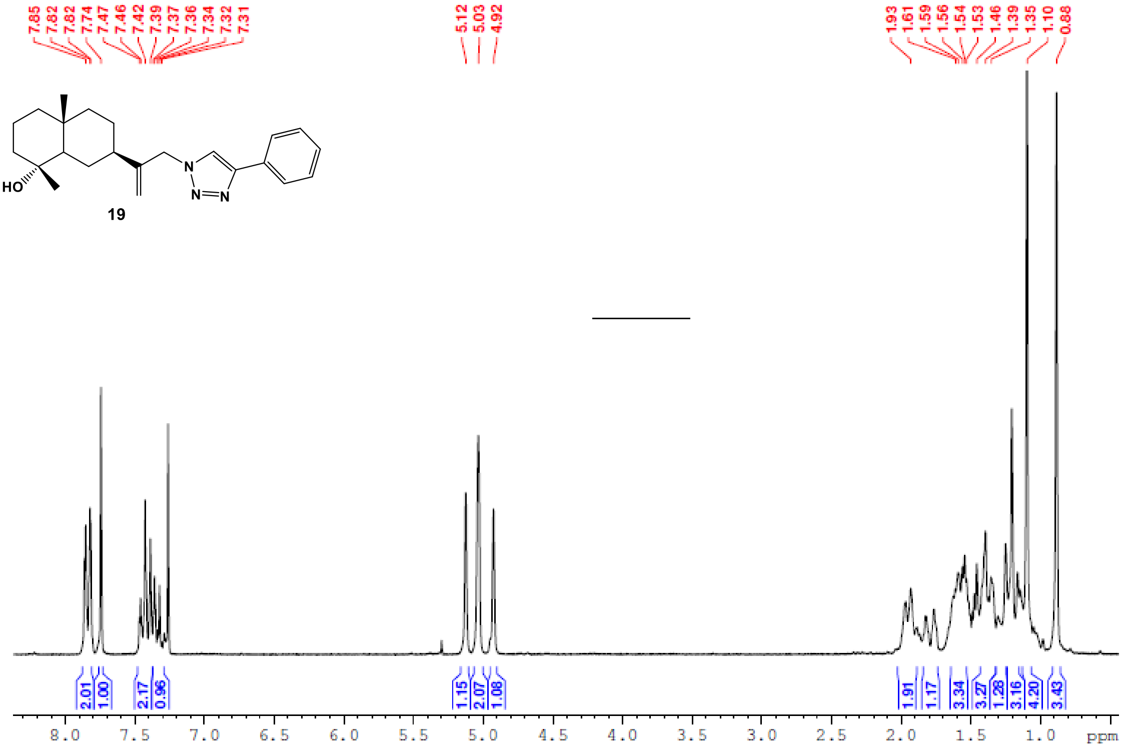

Figure S82. ^{1}H-NMR of **19.**

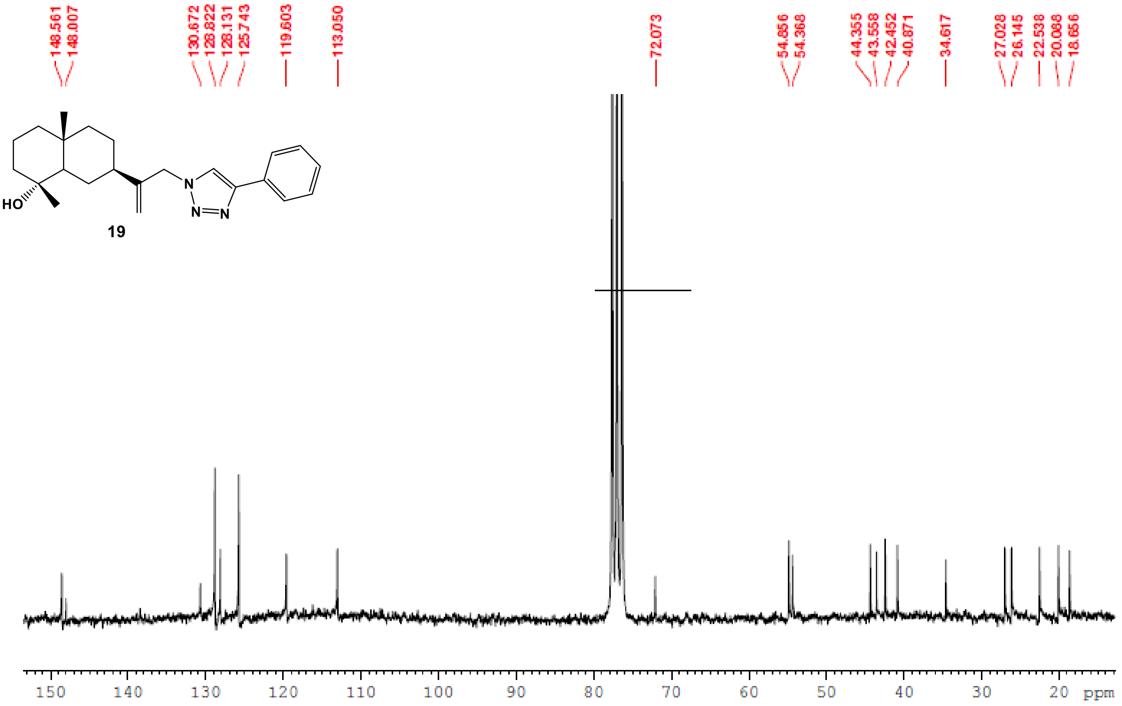

Figure S83. ^{13}C-NMR of **19.**

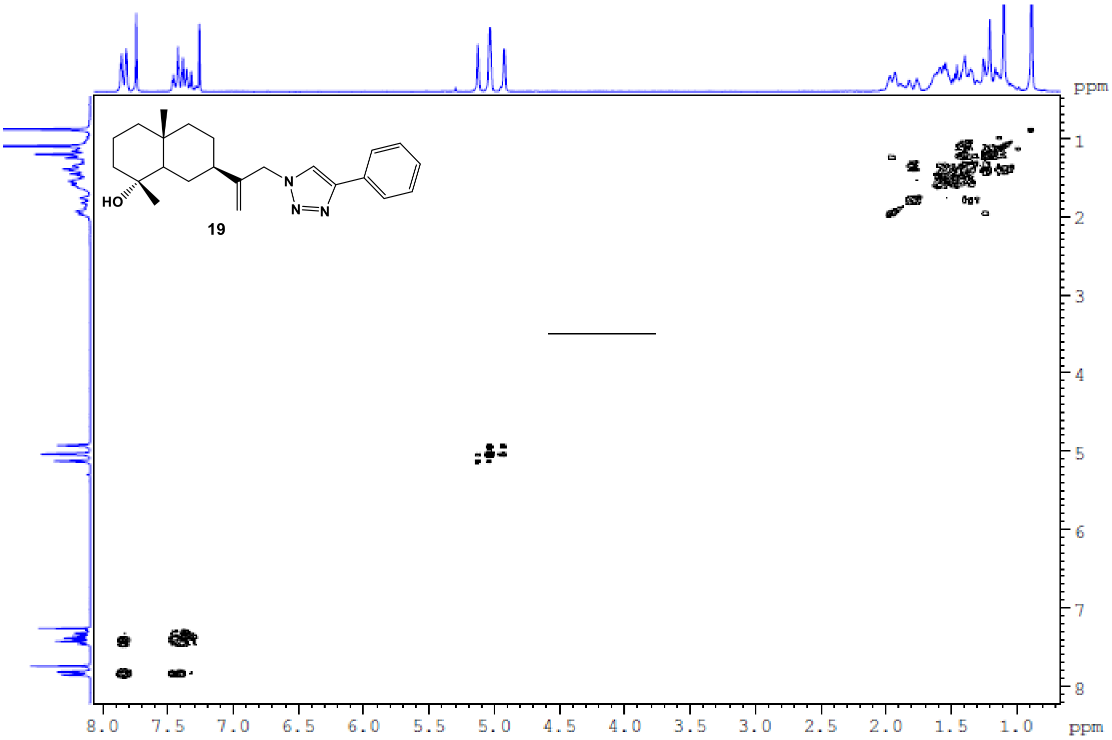

Figure S84. COSY of **19.**

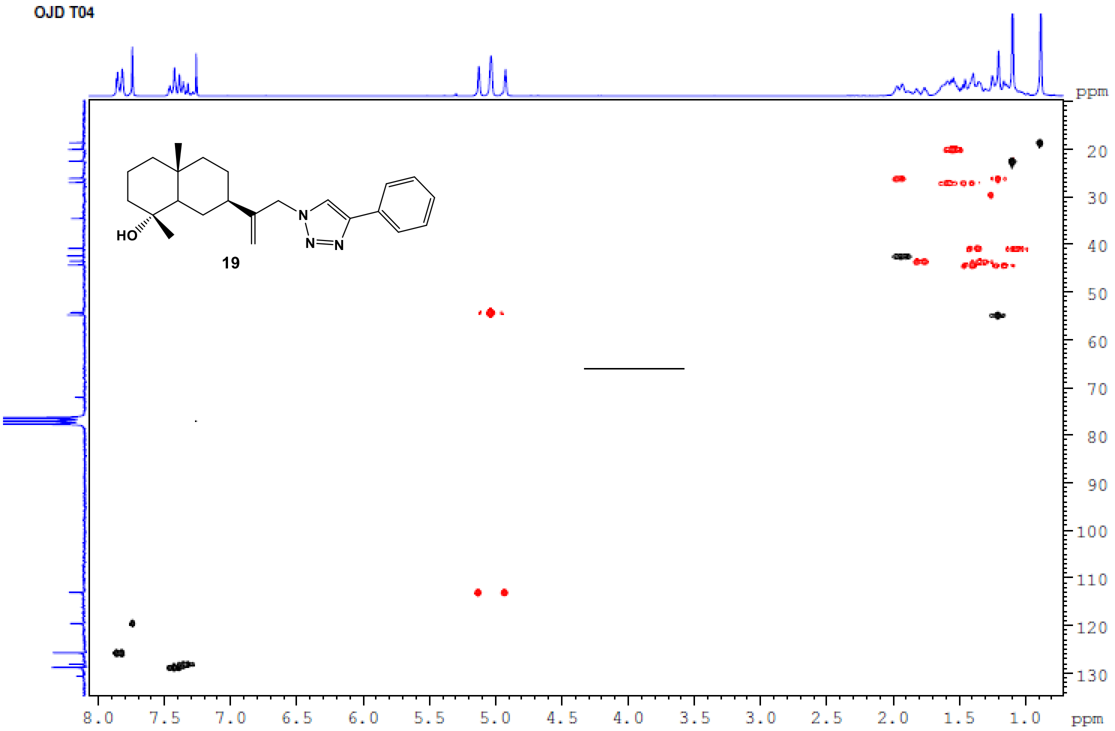

Figure S85. HSQC of **19.**

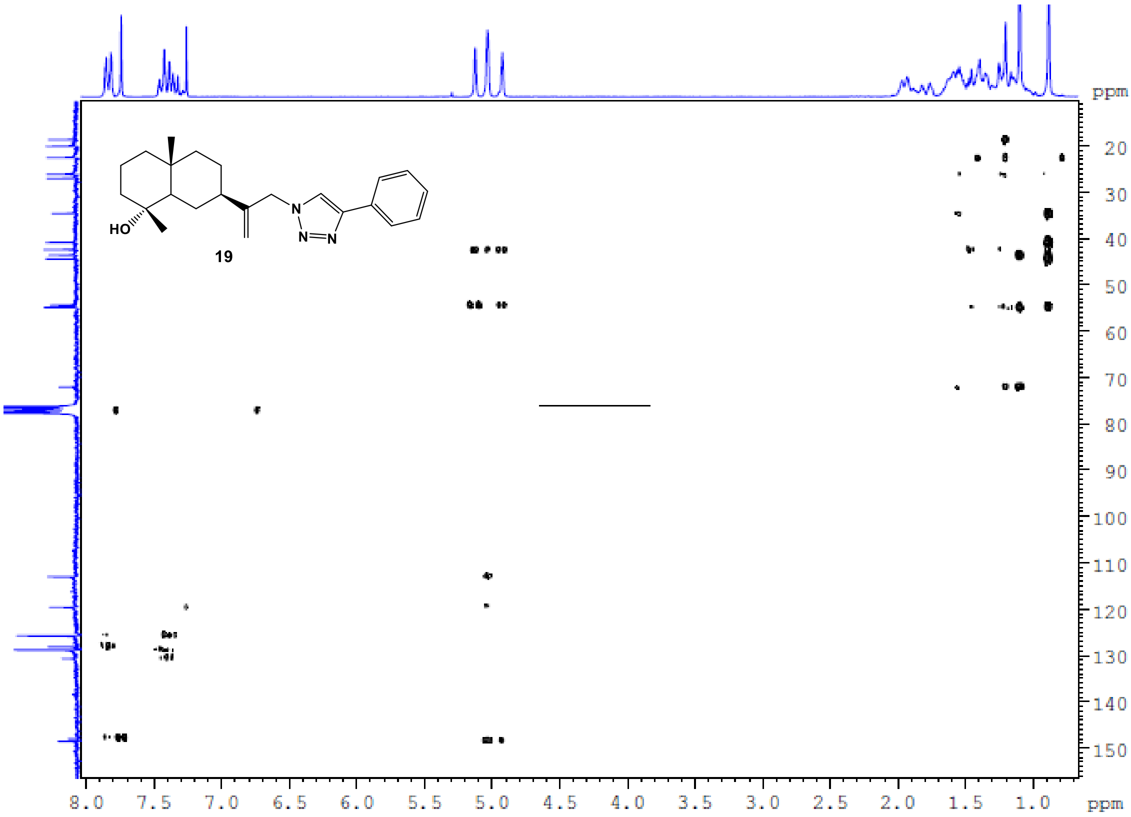

Figure S86. HMBC of **19.**

5. HRMS-ES of compounds 1-15

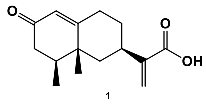

Elemental Composition Report **Page 1**

Multiple Mass Analysis: 3 mass(es) processed
Tolerance = 5.0 PPM / DBE: min = -1.5, max = 120.0
Element prediction: Off
Number of isotope peaks used for i-FIT = 3

Monoisotopic Mass, Even Electron Ions
1291 formula(e) evaluated with 7 results within limits (all results (up to 1000) for each mass)
Elements Used:
C: 0-120 H: 0-70 N: 0-5 O: 0-6 Na: 0-2
Osvaldo
(ESI-18) (206) Osvaldo Donadel (Acido Tessarico) 44 (1.479)

2: TOF MS ES+
2.51e+002

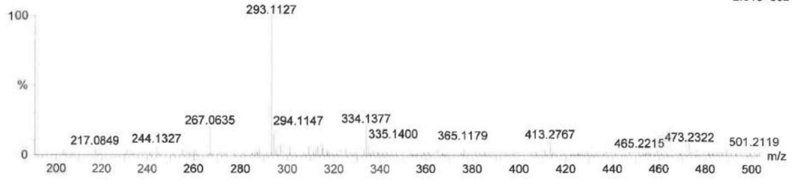

Mass	RA	Calc. Mass	mDa	PPM	DBE	i-FIT	i-FIT (Norm)	Formula
Minimum:	20.00				-1.5			
Maximum:	100.00		5.0	5.0	120.0			
267.0635	20.16	267.0633	0.2	0.7	8.5	12.7	0.9	C14 H12 O4 Na
		267.0623	1.2	4.5	10.5	13.0	1.2	C13 H9 N4 Na2
		267.0647	-1.2	-4.5	13.5	13.1	1.3	C15 H8 N4 Na
293.1127	100.00	293.1130	-0.3	-1.0	5.5	9.6	1.0	C15 H19 O3 Na2
		293.1137	-1.0	-3.4	7.5	9.7	1.1	C14 H17 N2 O5
		293.1113	1.4	4.8	4.5	9.8	1.2	C12 H18 N2 O5 Na
334.1377	21.34	334.1379	-0.2	-0.6	5.5	15.2	0.0	C14 H21 N3 O5 Na

Figure S87. HRMS-ES of **1.**

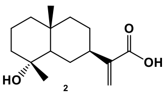

2

Elemental Composition Report **Page 1**

Multiple Mass Analysis: 2 mass(es) processed
Tolerance = 5.0 PPM / DBE: min = -1.5, max = 120.0
Element prediction: Off
Number of isotope peaks used for i-FIT = 3

Monoisotopic Mass, Even Electron Ions
824 formula(e) evaluated with 4 results within limits (all results (up to 1000) for each mass)
Elements Used:
C: 0-120 H: 0-70 N: 0-5 O: 0-6 Na: 0-2
Osvaldo
(ESI-18) (204) Osvaldo Donadel (Ac Ilicico)) 16 (0.661) 1: TOF MS ES+ 1.62e+004

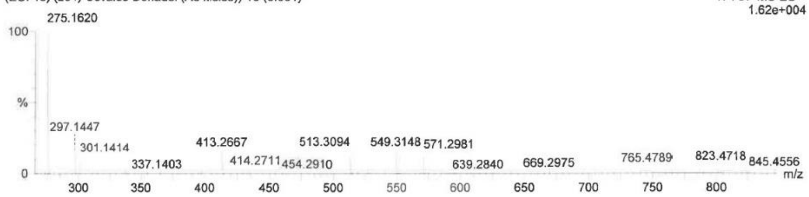

Minimum:	20.00				-1.5			
Maximum:	100.00		5.0	5.0	120.0			
Mass	RA	Calc. Mass	mDa	PPM	DBE	i-FIT	i-FIT (Norm)	Formula
275.1620	100.00	275.1623	-0.3	-1.1	3.5	17.5	0.2	C15 H24 O3 Na
		275.1607	1.3	4.7	2.5	18.9	1.6	C12 H23 N2 O5
297.1447	28.11	297.1443	0.4	1.3	3.5	23.0	0.6	C15 H23 O3 Na2
		297.1450	-0.3	-1.0	5.5	23.2	0.8	C14 H21 N2 O5

Figure S88. HRMS-ES of **2.**

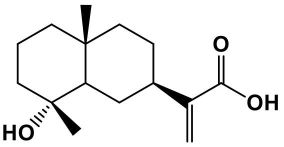

3

Elemental Composition Report **Page 1**

Tolerance = 5.0 PPM / DBE: min = -1.5, max = 120.0
Element prediction: Off
Number of isotope peaks used for i-FIT = 3

Monoisotopic Mass, Even Electron Ions
372 formula(e) evaluated with 1 results within limits (all results (up to 1000) for each mass)
Elements Used:
C: 0-120 H: 0-70 N: 0-5 O: 0-6 Na: 0-2
Osvaldo
(ESI-18) (205) Osvaldo Donadel (Alcohol Ilicico)) 19 (0.801) 1: TOF MS ES+ 5.69e+003

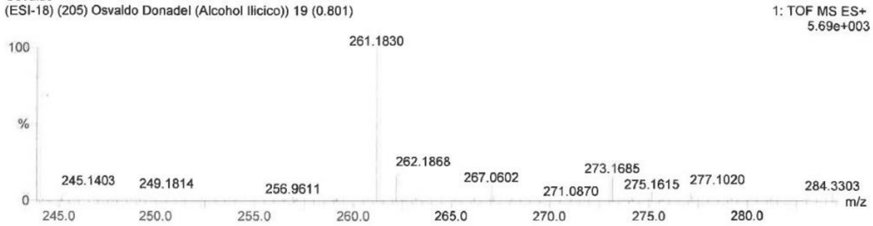

Minimum:	20.00				-1.5			
Maximum:	100.00		5.0	5.0	120.0			
Mass	RA	Calc. Mass	mDa	PPM	DBE	i-FIT	i-FIT (Norm)	Formula
261.1830	100.00	261.1831	-0.1	-0.4	2.5	14.5	0.0	C15 H26 O2 Na

Figure S89. HRMS-ES of **3.**

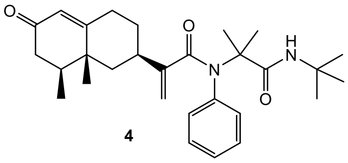

4

Elemental Composition Report **Page 1**

Tolerance = 5.0 PPM / DBE: min = -10.0, max = 1000.0
Element prediction: Off
Number of isotope peaks used for i-FIT = 2

Monoisotopic Mass, Even Electron Ions
1004 formula(e) evaluated with 4 results within limits (all results (up to 1000) for each mass)
Elements Used:
C: 0-70 H: 0-100 N: 0-4 O: 0-15 Na: 0-1
ESI (16-798) Maria F B (MFB -054) 116 (4.052) 2: TOF MS ES+ 6.58e+001

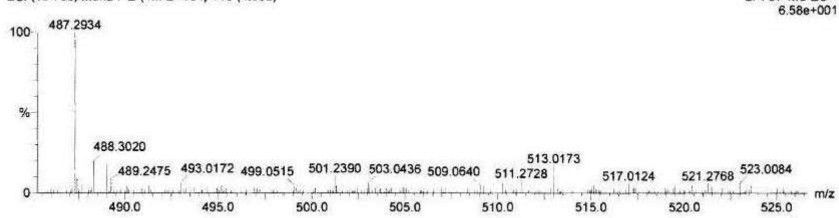

Mass	RA	Calc. Mass	mDa	PPM	DBE	i-FIT	Formula
Minimum:	50.00				-10.0		
Maximum:	100.00		100.0	5.0	1000.0		
487.2934	100.00	487.2937	-0.3	-0.6	10.5	2.0	C29 H40 N2 O3 Na
		487.2942	-0.8	-1.6	-7.5	0.0	C16 H48 O14 Na
		487.2920	1.4	2.9	9.5	1.4	C26 H39 N4 O5
		487.2955	-2.1	-4.3	-2.5	0.0	C17 H44 N4 O10 Na

Figure S90. HRMS-ES of **4.**

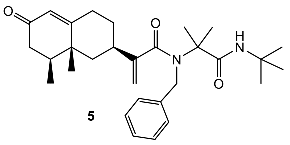

5

Elemental Composition Report **Page 1**

Multiple Mass Analysis: 2 mass(es) processed
Tolerance = 5.0 PPM / DBE: min = -10.0, max = 1000.0
Element prediction: Off
Number of isotope peaks used for i-FIT = 2

Monoisotopic Mass, Even Electron Ions
1304 formula(e) evaluated with 5 results within limits (all results (up to 1000) for each mass)
Elements Used:
C: 0-70 H: 0-100 N: 0-2 O: 0-16 Na: 0-1
ESI (16-867) Maria F (MFB-055A) 61 (2.128) 2: TOF MS ES+ 1.10e+003

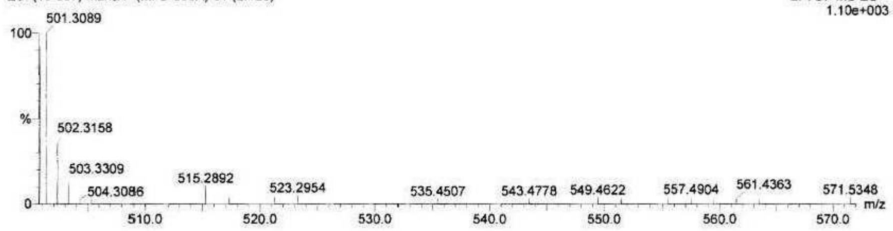

Mass	RA	Calc. Mass	mDa	PPM	DBE	i-FIT	Formula
Minimum:	20.00				-10.0		
Maximum:	100.00		100.0	5.0	1000.0		
501.3089	100.00	501.3093	-0.4	-0.8	10.5	0.2	C30 H42 N2 O3 Na
		501.3082	0.7	1.4	-8.5	47.4	C14 H49 N2 O16
		501.3098	-0.9	-1.8	-7.5	34.3	C17 H50 O14 Na
502.3158	36.02	502.3169	-1.1	-2.2	8.5	0.2	C29 H44 N O6
		502.3145	1.3	2.6	5.5	0.4	C27 H45 N O6 Na

Figure S91. HRMS-ES of **5.**

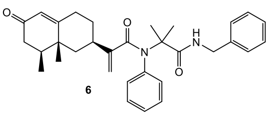

6

Elemental Composition Report **Page 1**

Multiple Mass Analysis: 2 mass(es) processed
Tolerance = 5.0 PPM / DBE: min = -10.0, max = 1000.0
Element prediction: Off
Number of isotope peaks used for i-FIT = 2

Monoisotopic Mass, Even Electron Ions
1355 formula(e) evaluated with 5 results within limits (all results (up to 1000) for each mass)
Elements Used:
C: 0-70 H: 0-100 N: 0-2 O: 0-16 Na: 0-1
ESI (16-866) Maria F (MFB-061) 130 (4.528) 2: TOF MS ES+ 5.11e+003

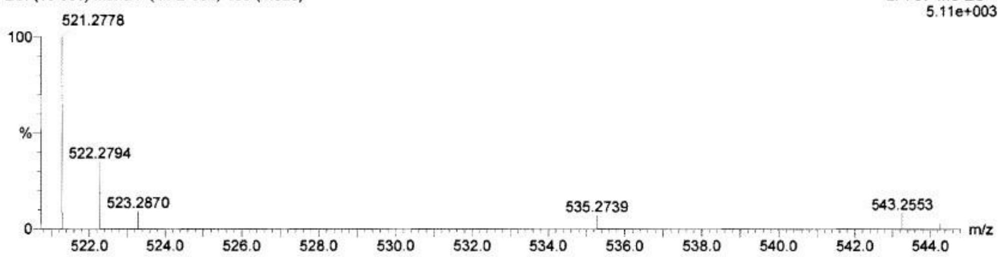

Minimum:	20.00				-10.0		
Maximum:	100.00		100.0	5.0	1000.0		
Mass	RA	Calc. Mass	mDa	PPM	DBE	i-FIT	Formula
521.2778	100.00	521.2780	-0.2	-0.4	14.5	2.3	C32 H38 N2 O3 Na
		521.2785	-0.7	-1.3	-3.5	101.7	C19 H46 O14 Na
		521.2769	0.9	1.7	-4.5	150.6	C16 H45 N2 O16
522.2794	34.95	522.2797	-0.3	-0.6	21.5	58.3	C38 H36 N O
		522.2773	2.1	4.0	18.5	46.4	C36 H37 N O Na

Figure S92. HRMS-ES of **6.**

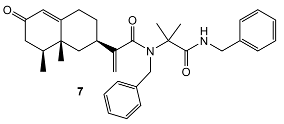

7

Elemental Composition Report **Page 1**

Multiple Mass Analysis: 2 mass(es) processed
Tolerance = 5.0 PPM / DBE: min = -10.0, max = 1000.0
Element prediction: Off
Number of isotope peaks used for i-FIT = 2

Monoisotopic Mass, Even Electron Ions
1393 formula(e) evaluated with 6 results within limits (all results (up to 1000) for each mass)
Elements Used:
C: 0-70 H: 0-100 N: 0-2 O: 0-16 Na: 0-1
ESI (16-863) Maria F (MFB-060) 76 (2.653) 2: TOF MS ES+ 1.08e+003

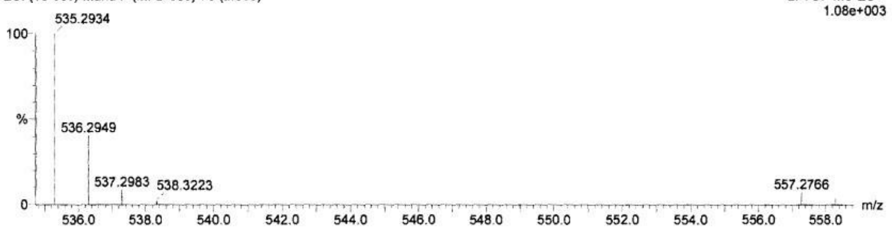

Minimum:	20.00				-10.0		
Maximum:	100.00		100.0	5.0	1000.0		
Mass	RA	Calc. Mass	mDa	PPM	DBE	i-FIT	Formula
535.2934	100.00	535.2937	-0.3	-0.6	14.5	0.6	C33 H40 N2 O3 Na
		535.2942	-0.8	-1.5	-3.5	34.3	C20 H48 O14 Na
		535.2926	0.8	1.5	-4.5	46.4	C17 H47 N2 O16
		535.2961	-2.7	-5.0	17.5	0.2	C35 H39 N2 O3
536.2949	40.62	536.2953	-0.4	-0.7	21.5	26.9	C39 H38 N O
		536.2929	2.0	3.7	18.5	22.9	C37 H39 N O Na

Figure S93. HRMS-ES of **7.**

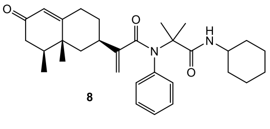

8

Elemental Composition Report **Page 1**

Multiple Mass Analysis: 2 mass(es) processed
Tolerance = 5.0 PPM / DBE: min = -1.5, max = 120.0
Element prediction: Off
Number of isotope peaks used for i-FIT = 3

Monoisotopic Mass, Even Electron Ions
1459 formula(e) evaluated with 7 results within limits (all results (up to 1000) for each mass)
Elements Used:
C: 0-120 H: 0-70 N: 0-5 O: 0-6 Na: 0-2
Osvaldo
(ESI-18) (202) Osvaldo Donadel (MFB 79) 50 (1.688) Cm (50:69)

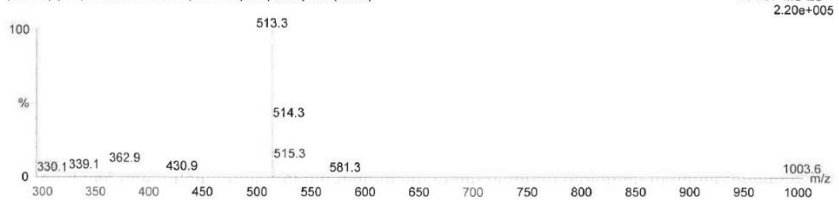

Mass	RA	Calc. Mass	mDa	PPM	DBE	i-FIT	i-FIT (Norm)	Formula
Minimum:	20.00				-1.5			
Maximum:	100.00		5.0	5.0	120.0			
513.3099	100.00	513.3077	2.2	4.3	10.5	20.7	0.1	C28 H41 N4 O5
		513.3093	0.6	1.2	11.5	22.6	2.0	C31 H42 N2 O3 Na
		513.3117	-1.8	-3.5	14.5	27.0	6.4	C33 H41 N2 O3
		513.3109	-1.0	-1.9	12.5	27.0	6.5	C34 H43 O Na2
514.3099	34.32	514.3086	1.3	2.5	15.5	34.5	0.6	C35 H41 N O Na
		514.3110	-1.1	-2.1	18.5	35.3	1.3	C37 H40 N O
		514.3121	-2.2	-4.3	3.5	35.8	1.8	C26 H46 N O6 Na2

Figure S94. HRMS-ES of **8.**

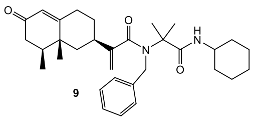

9

Elemental Composition Report **Page 1**

Multiple Mass Analysis: 2 mass(es) processed
Tolerance = 5.0 PPM / DBE: min = -1.5, max = 120.0
Element prediction: Off
Number of isotope peaks used for i-FIT = 3

Monoisotopic Mass, Even Electron Ions
1477 formula(e) evaluated with 9 results within limits (all results (up to 1000) for each mass)
Elements Used:
C: 0-120 H: 0-70 N: 0-5 O: 0-6 Na: 0-2
Osvaldo
(ESI-18) (203) Osvaldo Donadel (MFB 76) 36 (1.199)

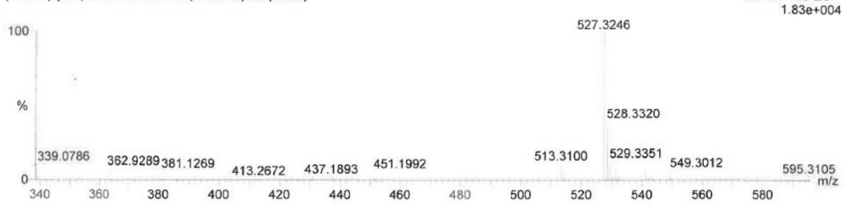

Mass	RA	Calc. Mass	mDa	PPM	DBE	i-FIT	i-FIT (Norm)	Formula
Minimum:	20.00				-1.5			
Maximum:	100.00		5.0	5.0	120.0			
527.3246	100.00	527.3250	-0.4	-0.8	11.5	18.2	0.6	C32 H44 N2 O3 Na
		527.3266	-2.0	-3.8	12.5	19.1	1.6	C35 H45 O Na2
		527.3233	1.3	2.5	10.5	19.3	1.7	C29 H43 N4 O5
		527.3226	2.0	3.8	8.5	20.0	2.5	C30 H45 N2 O3 Na2
528.3320	35.19	528.3314	0.6	1.1	11.5	16.1	1.1	C30 H43 N5 O2 Na
		528.3325	-0.5	-0.9	9.5	16.1	1.1	C31 H46 N O6
		528.3301	1.9	3.6	6.5	16.8	1.8	C29 H47 N O6 Na
		528.3331	-1.1	-2.1	12.5	17.0	2.0	C33 H44 N3 Na2
		528.3339	-1.9	-3.6	14.5	18.0	3.0	C32 H42 N5 O2

Figure S95. HRMS-ES of **9.**

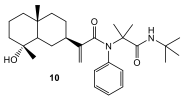

10

Elemental Composition Report **Page 1**

Tolerance = 5.0 PPM / DBE: min = -10.0, max = 1000.0
Element prediction: Off
Number of isotope peaks used for i-FIT = 2

Monoisotopic Mass, Even Electron Ions
1008 formula(e) evaluated with 5 results within limits (all results (up to 1000) for each mass)
Elements Used:
C: 0-70 H: 0-100 N: 0-4 O: 0-15 Na: 0-1
ESI (16-800) Maria F B (MFB -052) 81 (2.829) 2: TOF MS ES+ 1.02e+004

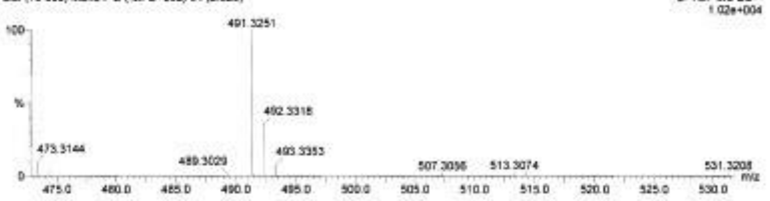

Minimum:	50.00				-10.0		
Maximum:	100.00		100.0	5.0	1000.0		

Mass	RA	Calc. Mass	mDa	PPM	DBE	i-FIT	Formula
491.3251	100.00	491.3250	0.1	0.2	8.5	5.0	C29 H44 N2 O3 Na
		491.3255	-0.4	-0.8	-9.5	359.8	C16 H52 O14 Na
		491.3268	-1.7	-3.5	-4.5	258.8	C17 H48 N4 O10 Na
		491.3233	1.8	3.7	7.5	24.9	C26 H43 N4 O5
		491.3274	-2.3	-4.7	11.5	0.7	C31 H43 N2 O3

Figure S96. HRMS-ES of **10.**

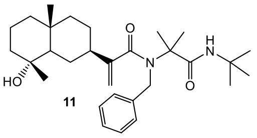

11

Elemental Composition Report **Page 1**

Tolerance = 5.0 PPM / DBE: min = -10.0, max = 1000.0
Element prediction: Off
Number of isotope peaks used for i-FIT = 2

Monoisotopic Mass, Even Electron Ions
1030 formula(e) evaluated with 5 results within limits (all results (up to 1000) for each mass)
Elements Used:
C: 0-70 H: 0-100 N: 0-4 O: 0-15 Na: 0-1
ESI (16-801) Maria F B (MFB -053) 100 (3.492) 2: TOF MS ES+ 4.61e+002

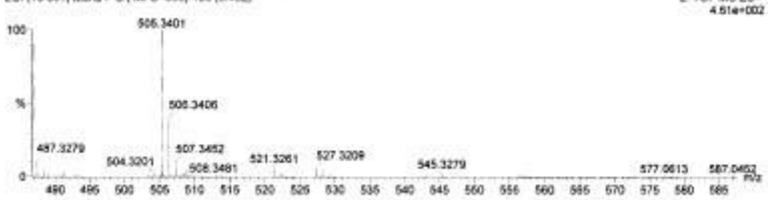

Minimum:	50.00				-10.0		
Maximum:	100.00		100.0	5.0	1000.0		

Mass	RA	Calc. Mass	mDa	PPM	DBE	i-FIT	Formula
505.3401	100.00	505.3406	-0.5	-1.0	8.5	1.3	C30 H46 N2 O3 Na
		505.3411	-1.0	-2.0	-9.5	21.1	C17 H54 O14 Na
		505.3390	1.1	2.2	7.5	2.9	C27 H45 N4 O5
		505.3377	2.4	4.7	2.5	5.1	C26 H49 O9
		505.3425	-2.4	-4.7	-4.5	16.1	C18 H50 N4 O10 Na

Figure S97. HRMS-ES of **11.**

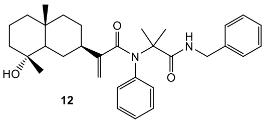

12

Elemental Composition Report Page [illegible]

Multiple Mass Analysis: 2 mass(es) processed
Tolerance = 5.0 PPM / DBE: min = -10.0, max = 1000.0
Element prediction: Off
Number of isotope peaks used for i-FIT = 2

Monoisotopic Mass, Even Electron Ions
1360 formula(e) evaluated with 5 results within limits (all results (up to 1000) for each mass)
Elements Used:
C: 0-70 H: 0-100 N: 0-2 O: 0-16 Na: 0-1
ESI (16-865) Maria F (MFB-056) 70 (3.051) 1: TOF MS ES+ 4.95e+001

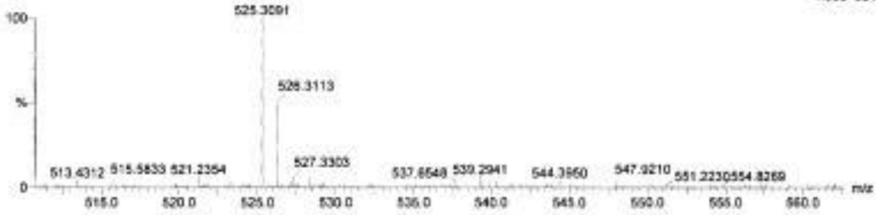

Minimum:	20.00				-10.0		
Maximum:	100.00		100.0	5.0	1000.0		

Mass	RA	Calc. Mass	mDa	PPM	DBE	i-FIT	Formula
525.3091	100.00	525.3093	-0.2	-0.4	12.5	0.8	C32 H42 N2 O3 Na
		525.3098	-0.7	-1.3	-5.5	3.4	C19 H50 O14 Na
		525.3082	0.9	1.7	-6.5	4.4	C16 H49 N2 O16
		525.3117	-2.6	-4.9	15.5	0.5	C34 H41 N2 O3
526.3113	51.17	526.3110	0.3	0.6	19.5	4.0	C38 H40 N O

Figure S98. HRMS-ES of **12.**

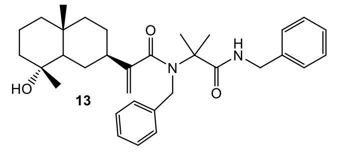

Elemental Composition Report Page 1

Multiple Mass Analysis: 2 mass(es) processed
Tolerance = 5.0 PPM / DBE: min = -10.0, max = 1000.0
Element prediction: Off
Number of isotope peaks used for i-FIT = 2

Monoisotopic Mass, Even Electron Ions
1389 formula(e) evaluated with 7 results within limits (all results (up to 1000) for each mass)
Elements Used:
C: 0-70 H: 0-100 N: 0-2 O: 0-16 Na: 0-1
ESI (16-868) Maria F (MFB-057) 43 (1.898) 1: TOF MS ES+ 5.55e+003

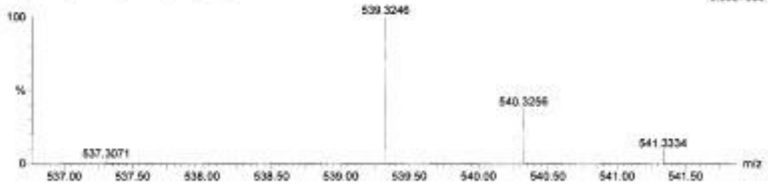

Minimum:	20.00				-10.0		
Maximum:	100.00		100.0	5.0	1000.0		

Mass	RA	Calc. Mass	mDa	PPM	DBE	i-FIT	Formula
539.3246	100.00	539.3250	-0.4	-0.7	12.5	0.3	C33 H44 N2 O3 Na
		539.3239	0.7	1.3	-6.5	183.3	C17 H51 N2 O16
		539.3255	-0.9	-1.7	-5.5	128.3	C20 H52 O14 Na
		539.3220	2.6	4.8	6.5	11.2	C29 H47 O9
540.3256	37.68	540.3266	-1.0	-1.9	19.5	91.8	C39 H42 N O
		540.3242	1.4	2.6	16.5	75.7	C37 H43 N O Na
		540.3231	2.5	4.6	-2.5	0.4	C21 H50 N O14

Figure S99. HRMS-ES of **13.**

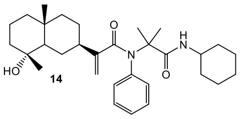

Elemental Composition Report

Page 1

Multiple Mass Analysis: 2 mass(es) processed

Tolerance = 5.0 PPM / DBE: min = -10.0, max = 1000.0
Element prediction: Off
Number of isotope peaks used for i-FIT = 2

Monoisotopic Mass, Even Electron Ions
792 formula(e) evaluated with 4 results within limits (all results (up to 1000) for each mass)
Elements Used:
C: 0-120 H: 0-70 N: 0-2 O: 0-10 Na: 0-1
(ESI 17-854) Victor (MFB-062) 1 (0.053)

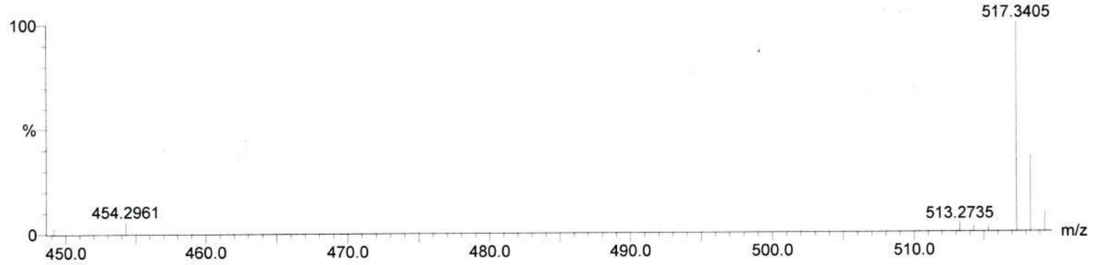

Minimum:	20.00				-10.0		
Maximum:	100.00		100.0	5.0	1000.0		
Mass	RA	Calc. Mass	mDa	PPM	DBE	i-FIT	Formula
517.3405	100.00	517.3406	-0.1	-0.2	9.5	0.0	C31 H46 N2 O3 Na
		517.3430	-2.5	-4.8	12.5	3.8	C33 H45 N2 O3
518.3444	35.81	518.3458	-1.4	-2.7	4.5	10.9	C28 H49 N O6 Na
		518.3423	2.1	4.1	16.5	59.9	C37 H44 N O

Figure S100. HRMS-ES of **14.**

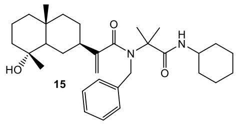

Elemental Composition Report

Page 1

Multiple Mass Analysis: 2 mass(es) processed

Tolerance = 5.0 PPM / DBE: min = -10.0, max = 1000.0
Element prediction: Off
Number of isotope peaks used for i-FIT = 2

Monoisotopic Mass, Even Electron Ions
792 formula(e) evaluated with 4 results within limits (all results (up to 1000) for each mass)
Elements Used:
C: 0-120 H: 0-70 N: 0-2 O: 0-10 Na: 0-1
(ESI 17-855) Victor (MFB-063) 52 (1.816)

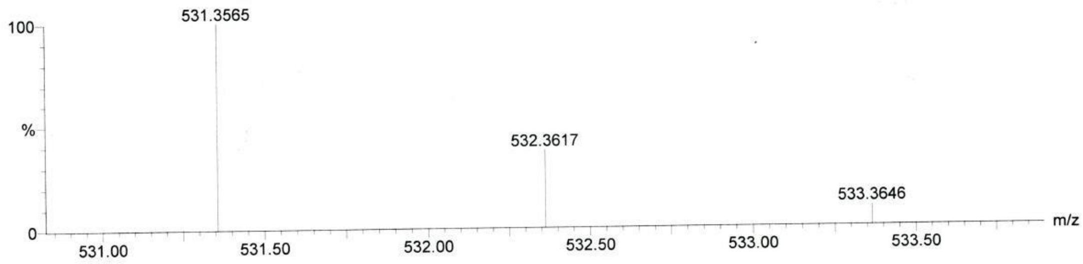

Minimum:	20.00				-10.0		
Maximum:	100.00		100.0	5.0	1000.0		
Mass	RA	Calc. Mass	mDa	PPM	DBE	i-FIT	Formula
531.3565	100.00	531.3563	0.2	0.4	9.5	0.1	C32 H48 N2 O3 Na
		531.3587	-2.2	-4.1	12.5	3.1	C34 H47 N2 O3
532.3617	37.18	532.3614	0.3	0.6	4.5	18.0	C29 H51 N O6 Na
		532.3638	-2.1	-3.9	7.5	28.9	C31 H50 N O6

Figure S101. HRMS-ES of **15.**

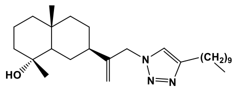

16

Elemental Composition Report

Page 1

Multiple Mass Analysis: 304 mass(es) processed - displaying only valid results
Tolerance = 20.0 PPM / DBE: min = -1.5, max = 50.0
Selected filters: None

Monoisotopic Mass, Odd and Even Electron Ions
4798 formula(e) evaluated with 92 results within limits (all results (up to 1000) for each mass)
Elements Used:
C: 1-27 H: 1-47 N: 0-3 O: 0-1
23-Oct-14-A 118 (3.003) Cn (Cen,2, 50.00, Ht)

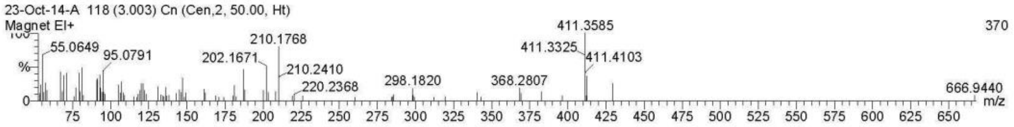

Minimum:	0.10				-1.5					
Maximum:	100.00		5.0	20.0	50.0					
Mass	RA	Calc. Mass	mDa	PPM	DBE	i-FIT	Formula			
429.3716	25.06	429.3719	-0.3	-0.7	6.0	5546064.0	C27	H47	N3	O
412.3660	36.05	412.3692	-3.2	-7.8	6.5	5546084.0	C27	H46	N3	
412.3305	13.25	412.3328	-2.3	-5.6	7.5	5546042.5	C26	H42	N3	O
411.3585	100.00	411.3613	-2.8	-6.8	7.0	2773105.0	C27	H45	N3	
411.3325	67.87	411.3375	-5.0	-12.2	7.5	2773053.5	C27	H43	N2	O
		411.3250	7.5	18.2	8.0	2773050.8	C26	H41	N3	O
396.3452	7.20	396.3504	-5.2	-13.1	7.0	5546032.5	C27	H44	N2	
		396.3379	7.3	18.4	7.5	5546032.5	C26	H42	N3	

Figure S102. HRMS-ES of **16.**

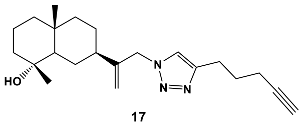

17

Elemental Composition Report

Page 1

Multiple Mass Analysis: 560 mass(es) processed - displaying only valid results
Tolerance = 20.0 PPM / DBE: min = -1.5, max = 50.0
Selected filters: None

Monoisotopic Mass, Odd and Even Electron Ions
8562 formula(e) evaluated with 251 results within limits (all results (up to 1000) for each mass)
Elements Used:
C: 1-23 H: 2-35 N: 0-3 O: 0-1
23-Oct-14-CAFAMMA 82 (2.087)

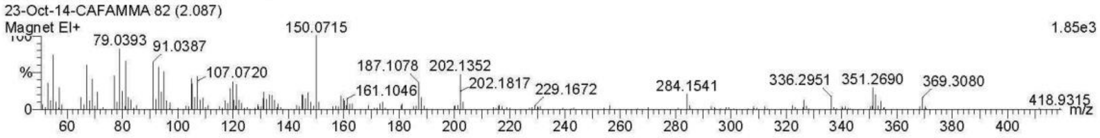

Minimum:	0.10				-1.5					
Maximum:	100.00		5.0	20.0	50.0					
Mass	RA	Calc. Mass	mDa	PPM	DBE	i-FIT	Formula			
369.2774	12.50	369.2780	-0.6	-1.6	8.0	2773034.8	C23	H35	N3	O
355.2646	1.72	355.2624	2.2	6.2	8.0	5546033.5	C22	H33	N3	O
354.2615	9.32	354.2671	-5.6	-15.8	8.0	2773024.8	C23	H34	N2	O
		354.2545	7.0	19.8	8.5	2773023.5	C22	H32	N3	O
353.2482	4.16	353.2467	1.5	4.2	9.0	78.6	C22	H31	N3	O
351.2690	29.33	351.2674	1.6	4.6	9.0	43.4	C23	H33	N3	
351.2496	29.12	351.2436	6.0	17.1	9.5	44.1	C23	H31	N2	O
350.2256	1.59	350.2232	2.4	6.9	10.5	401.7	C22	H28	N3	O
342.2616	0.85	342.2671	-5.5	-16.1	7.0	5546027.5	C22	H34	N2	O
341.2777	2.61	341.2831	-5.4	-15.8	7.0	2773013.3	C22	H35	N3	
		341.2719	5.8	17.0	7.0	2773013.0	C23	H35	N	O

Figure S103. HRMS-ES of **17.**

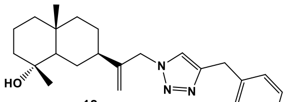

18

Elemental Composition Report

Page 1

Multiple Mass Analysis: 453 mass(es) processed - displaying only valid results
Tolerance = 20.0 PPM / DBE: min = -1.5, max = 50.0
Selected filters: None

Monoisotopic Mass, Odd and Even Electron Ions
6666 formula(e) evaluated with 165 results within limits (all results (up to 1000) for each mass)
Elements Used:
C: 1-24 H: 2-33 N: 0-3 O: 0-1
27-Oct-14-AAFAMMA 123 (3.131)
Magnet EI+

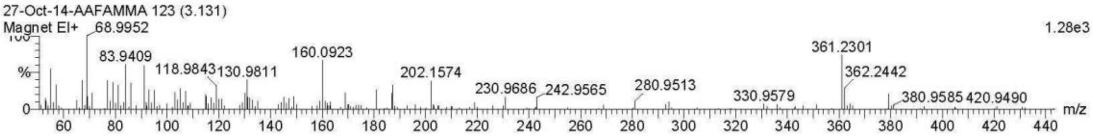

Minimum:	0.10				-1.5		
Maximum:	100.00		5.0	20.0	50.0		

Mass	RA	Calc. Mass	mDa	PPM	DBE	i-FIT	Formula
379.2619	17.70	379.2624	-0.5	-1.3	10.0	2773037.5	C24 H33 N3 O
364.2541	1.44	364.2515	2.6	7.1	10.0	2773040.0	C24 H32 N2 O
364.1466	0.89	364.1450	1.6	4.4	17.5	2773045.0	C24 H18 N3 O
363.2478	4.71	363.2436	4.2	11.6	10.5	30.2	C24 H31 N2 O
363.2277	4.16	363.2311	-3.4	-9.4	11.0	61.7	C23 H29 N3 O
362.2208	21.02	362.2232	-2.4	-6.6	11.5	40.8	C23 H28 N3 O
361.2301	73.59	361.2280	2.1	5.8	11.5	3.1	C24 H29 N2 O
351.2405	5.52	351.2436	-3.1	-8.8	9.5	5546051.5	C23 H31 N2 O
346.2078	2.67	346.2045	3.3	9.5	12.0	5546035.0	C23 H26 N2 O
346.1854	1.80	346.1919	-6.5	-18.8	12.5	5546030.0	C22 H24 N3 O

Figure S104. HRMS-ES of **18.**

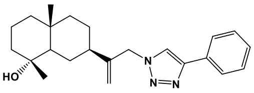

19

Elemental Composition Report

Page 1

Tolerance = 5.0 PPM / DBE: min = -10.0, max = 1000.0
Element prediction: Off
Number of isotope peaks used for i-FIT = 2

Monoisotopic Mass, Even Electron Ions
808 formula(e) evaluated with 3 results within limits (all results (up to 1000) for each mass)
Elements Used:
C: 0-70 H: 0-100 N: 0-4 O: 0-15 Na: 0-1
ESI (16-799) Maria F B (MFB -TO(V) 71 (2.484)

2: TOF MS ES+
6.77e+001

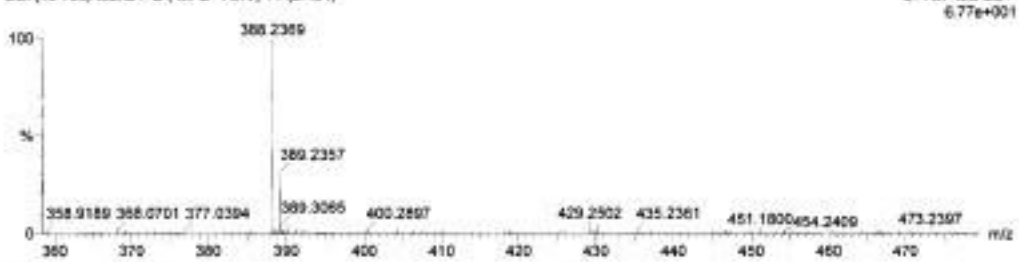

Minimum:	50.00				-10.0		
Maximum:	100.00		100.0	5.0	1000.0		

Mass	RA	Calc. Mass	mDa	PPM	DBE	i-FIT	Formula
388.2369	100.00	388.2370	-0.1	-0.3	-8.5	3.7	C10 H39 N O12 Na
		388.2365	0.4	1.0	9.5	0.2	C23 H31 N3 O Na
		388.2354	1.5	3.9	-9.5	4.8	C7 H38 N3 O14

Figure S105. HRMS-ES of **19.**
